# Facile preparation of fluorine-containing 2,3-epoxypropanoates and their epoxy ring-opening reactions with various nucleophiles

**DOI:** 10.3762/bjoc.20.206

**Published:** 2024-09-25

**Authors:** Yutaro Miyashita, Sae Someya, Tomoko Kawasaki-Takasuka, Tomohiro Agou, Takashi Yamazaki

**Affiliations:** 1 Division of Applied Chemistry, Institute of Engineering, Tokyo University of Agriculture and Technology, 2-24-16 Nakamachi, Koganei 184-8588, Japan; 2 Department of Material Science, Graduate School of Science, University of Hyogo, 3-2-1 Koto, Kamigori-cho, Ako-gun, Hyogo 678-1297, Japan

**Keywords:** α,β-unsaturated esters, epoxyesters, fluorine, Grignard-based cuprates, nucleophiles

## Abstract

We describe herein a facile method to access 2,3-epoxyesters with fluorine-containing substituents at their 3-position starting from the corresponding enoates by utilization of the low-costed and easy-to-handle reagent, NaOCl·5H_2_O. Because very little has been disclosed about the reactivity of such 2,3-epoxyesters, their epoxy ring opening by a variety of nucleophiles was carried out and we succeeded in clarifying these chemo- as well as regioselective processes proceeding via the S_N_2 mechanism to mainly afford 2-substituted 3-hydroxyesters usually in a highly *anti* selective manner.

## Introduction

**Scheme 1:**
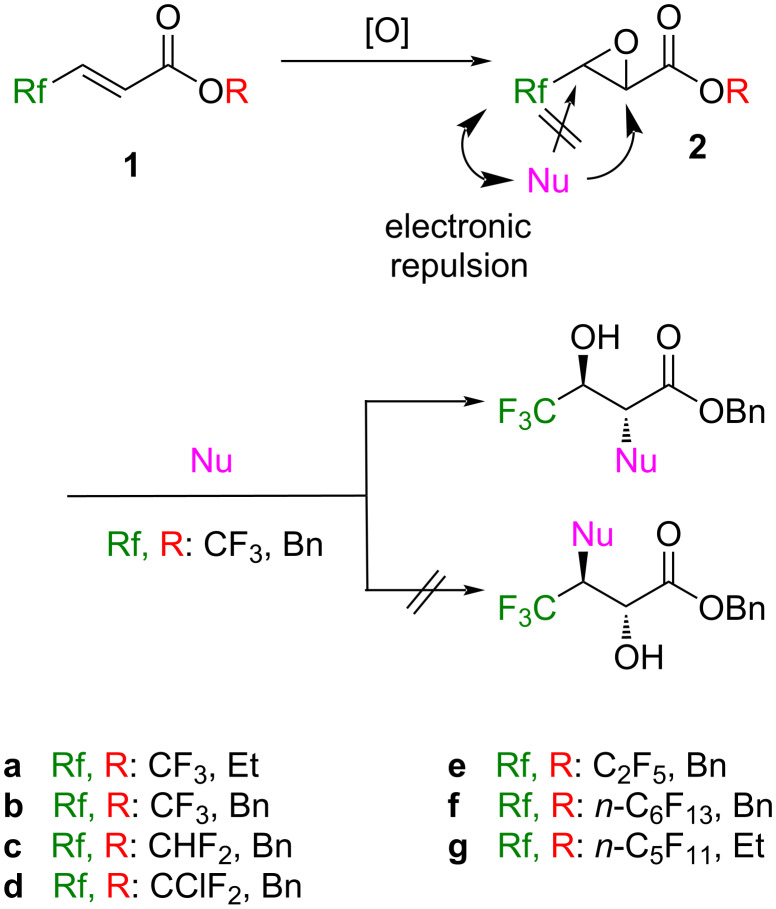
Expectation of the regio- as well as stereoselective reactions of **2**.

Fluorine-containing compounds have been utilized in diverse fields due to their special character originating from unique fluorine atoms or fluorinated groups [1–7]. During our study in this area, ethyl 4,4,4-trifluorobut-2-enoate (**1a**) has been frequently employed as a potent and convenient Michael acceptor towards a variety of enolates [8–15] as well as organometallic species [16–19]. At least in part, its high reactivity was considered to be due to the significantly lower-lying LUMO energy level by the attachment of electron-withdrawing trifluoromethyl (CF_3_) and ethoxycarbonyl groups [20]. As we previously pointed out [10,21], the effective intramolecular interaction between fluorine and metals would also facilitate the smooth progress of these reactions. Such high potential of **1a** allowed us to apply it to nucleophilic epoxidation because the resultant epoxyester **2a** is recognized as an intriguing building block (Scheme 1).

Another expectation to **2a** is the high regio- and stereoselectivities of its epoxy ring opening specifically occurring at the 2 position in an S_N_2 manner, when it is treated with appropriate nucleophiles (Nu), leading to the formation of the 2-substituted 3-hydroxyesters with 2,3-*anti* stereochemistry. These characteristic outcomes would stem from a result of the electronically repulsive interaction between the incoming nucleophiles and an electronically strongly negative CF_3_ group, and the anticipated clean S_N_2 mechanism of epoxides in general, respectively. This is interestingly compared with the case of **2a** with nonfluorinated Rf groups which sometimes suffered from the contamination of the regioisomers as a consequence of the regiorandom attack of nucleophiles [22–26].

Despite such significant advantage, compound **2a** was previously prepared only by 1) the LDA-mediated iodination-intramolecular ring closure sequence from the corresponding chiral 4,4,4-trifluoro-3-hydroxybutyrate at low temperature [27–30], and 2) *t*-BuO_2_Li-mediated transformation of the enoates like **1g** at −78 °C [31–32] and, to the best of our knowledge, no report has appeared on the convenient methods applicable to the larger scale synthesis to get access to the synthetically quite useful compounds like **2a** [33–34].

Under such situations, we envisaged that the high electrophilicity of compound **1a** would permit the usage of the extraordinarily convenient and mild reagent NaOCl [35–38] which opens the promising route for the preparation of **2a**. Moreover, the fact that only very limited examples are known for their synthetic application except for the synthesis of 4,4,4-trifluorothreonine [29,33], stereoselective ring opening with organometallic species [29], and so on [32] also stimulated our interest. In this article, we would like to describe in detail the results of the preparation of epoxyesters **2** with various Rf groups as well as their reactivity with diverse nucleophiles [39].

## Results and Discussion

### Preparation of (*E*)-2,3-epoxypropanoates **2** with Rf groups at the 3 position

Because the urea·H_2_O_2_ complex proved its usefulness for the epoxidation of the β-CF_3_-α,β-unsaturated ketones [40], we applied this method at first for the epoxidation of **1b**. However, contrary to our anticipation, only a total recovery of the substrate was observed, and further search for an oxidant reached the usage of a NaClO aqueous solution with its convenient handling and availability at a low cost. Following to the reported protocol [41], although a catalytic amount of Al_2_O_3_ and MgO worked nicely (entries 1 and 2 in Table 1), it was clarified that these additives were not necessary for the attainment of the same level of chemical yields (entries 3 vs 1 and 2). The drawback of this sequence was the isolated yield of **2b** no more than 70% which was, at least in part, due to the production of the undesired hydrolyzed products from **1b** and/or **2b** under the alkaline conditions of this epoxidation reagent. This was experimentally proved by the detection of benzaldehyde which was considered to be formed by the NaClO-mediated oxidation of benzyl alcohol generated by hydrolysis. Changing the oxidizing reagent to crystalline NaClO·5H_2_O nicely solved the problem with the realization of 86% isolated yield of **2b** by the utilization of this oxidant (2 equiv) at 0 °C with 6 h stirring (entry 8 in Table 1). We also tried to apply these conditions to other fluorine-containing substrates **1c**–**f** and successfully obtained good to high yields of the desired products **2c**–**f**, respectively (entries 10–13 in Table 1). The requirement of longer reaction time and higher temperature especially in the case of compounds **1e** and **1f** as well as the high loading of the oxidant in the latter might be due to their higher oleophobicity by possessing longer Rf chains. For all instances, epoxyesters **2** were obtained as single *E*-isomers, and based on the result obtained by the *t*-BuO_2_Li reagent [31], we speculated that NaClO·5H_2_O would similarly work for the corresponding *Z*-**1** with retention of stereochemistry.

**Table 1:** Optimization of epoxidation conditions of **1**.

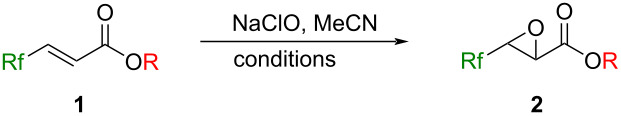					

Entry	Sub.	NaClO^a^	(equiv)	Conditions	Isolated yield^b^ (%)

1^c^	**1b**	AQ	1.0	25 °C, 6 h	59 (67)
2^d^	**1b**	AQ	1.0	25 °C, 5 h	(69)
3	**1b**	AQ	1.0	25 °C, 4.5 h	60 (63)
4	**1b**	S	1.0	20 °C, 3 h	(65)
5	**1b**	S	1.5	20 °C, 3 h	(83)
6	**1b**	S	1.5	20 °C, 6 h	(84)
7	**1b**	S	1.5	0 °C, 6 h	(89)
8	**1b**	S	2.0	0 °C, 6 h	86 (94)
9	**1b**	S	3.0	0 °C, 6 h	(83)
10	**1c**	S	2.0	0 °C, 6 h	79
11	**1d**	S	2.0	0 °C, 6 h	78
12	**1e**	S	2.0	0 °C, 6 h: 20 °C, 12 h	73
13	**1f**	S	5.0	20 °C, 48 h	61

^a^AQ: a 5% aqueous solution, S: solid of NaClO·5H_2_O; ^b^the yields determined by ^19^F NMR were described in the parentheses; ^c^10 mol % of Al_2_O_3_ was added; ^d^20 mol % of MgO was added.

**Scheme 2:**
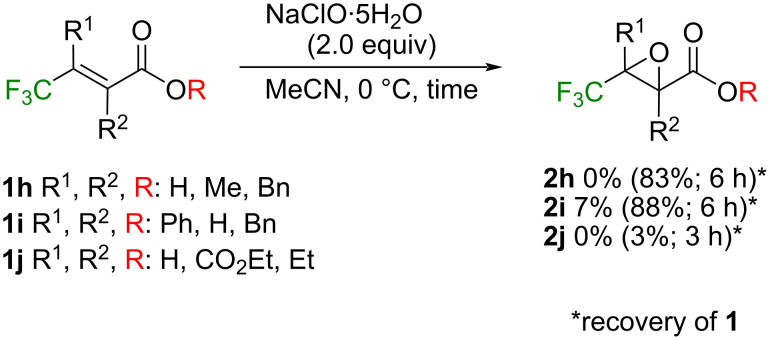
Attempts of the present epoxidation to other α,β-unsaturated esters, **1h**–**j**.

The procedure found here was also applied to the three representative CF_3_-containing α,β-unsaturated esters,**1h**–**j** [42] with different substitution patterns (Scheme 2).

The subjection of the compounds **1h** and **1i** to the standard conditions described above resulted in high recovery of the substrates, which could be explained by their higher LUMO + 1 energy levels responsible for the epoxidation [43]. Extensive decomposition was observed in the case of **1j** even in a shorter period possibly because of its significantly high electrophilicity by the attachment of three strongly electron-withdrawing moieties.

### Reactions of (*E*)-3-Rf-2,3-epoxypropanoates **2** with amines, thiols, and metal halides

Because the epoxide ring opening is known to occur in an S_N_2 fashion, compounds **2** were recognized as versatile building blocks for the construction of 2-amino-3-hydroxypropanoates with 2,3-*anti* stereochemistry, if appropriate amines work nicely in a nucleophilic manner [44].

After the brief optimization of the conditions for the reaction of **2b** and *p*-anisidine, good yields with high stereoselectivity were similarly recorded for the other substrates **2c** and **2d** possessing different Rf groups at the 3 position (Table 2, entries 1–3). Mixing of **2b** with different primary (entries 4–7 in Table 2) and secondary (entries 8 and 9) amines led to the formation of the respective products in high to excellent yields without detection of any regio- as well as stereoisomers. The chirality contained in amines did not work efficiently for the stereochemical induction of the products (entries 6 and 7 in Table 2). In the case of secondary amines, the sterically demanding dibenzylamine failed in this transformation and recovery of **2b** was observed (Table 2, entry 10). As was pointed out in the introductory section, the highly regioselective epoxy ring opening is well compared with the case when the nonfluorinated substrate (Ph instead of CF_3_ in **2b**) was employed [25–26].

**Table 2:** Reactions of **2** with a variety of amines.

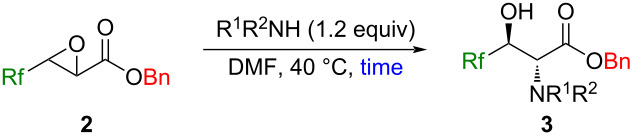					

Entry	Rf	R^1^	R^2^	Time (h)	Isolated yield (%)

1^a^	**CF** **_3_**	4-MeOC_6_H_4_	H	19	78 (**3ba**)
2^a^	**CHF** **_2_**	4-MeOC_6_H_4_	H	19	59 (**3ca**)
3^a^	**CClF** **_2_**	4-MeOC_6_H_4_	H	19	76 (**3da**)
4	**CF** **_3_**	PhCH_2_	H	7	86 (**3bb**)
5	**CF** **_3_**	*n*-Bu	H	7	48 (**3bc**)
6	**CF** **_3_**	PhCH(CH_3_)	H	18	77^c^ (**3bd**)
7^b^	**CF** **_3_**	EtCH(Me)CH(CO_2_Bn)	H	24	72^c^ (**3be**)
8	**CF** **_3_**	Et	Et	7	83 (**3bf**)
9	**CF** **_3_**	(CH_2_)_4_		7	56 (**3bg**)
10	**CF** **_3_**	Bn	Bn	7	–^d^

^a^EtOH was used as the solvent and the reaction temperature was 50 °C; ^b^reaction was performed with 2.5 equiv of benzyl isoleucinate·TsOH and Et_3_N; ^c^consisted of 53:47 diastereomers in both cases; ^d^no reaction was observed.

With the successful employment of amines as nucleophiles for the epoxy ring opening in a highly stereoselective fashion, we next turned our attention to thiols. Optimization of the reaction conditions based on the ones for amines clarified the tendency that the longer reaction time and the higher temperature decreased the chemical yields as well as the diastereomeric ratios (Table 3, entries 1–4). The higher p*K*_a_ values of the carbonyl α-proton of **4** (for example, the p*K*a values of the protons of X-C*H*_2_C(O)Ph in DMSO were reported to be 17.1 (X: PhS) [45] and 20.3 (X: Ph_2_N) [46]) would result in the contamination of the stereoisomers when compared with the case of the compounds **3** [47–48]. Because control of the amount of PhCH_2_SH to 1.0 equiv did not give a positive effect, the conditions in entry 4 (Table 3) were eventually determined as the best.

**Table 3:** Reactions of **2** with a variety of thiols.

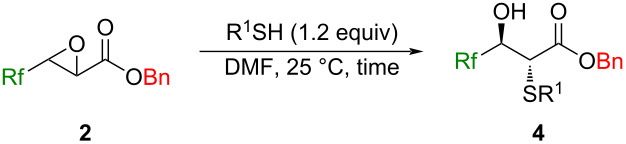					

Entry	Rf	R^1^	Time (h)	Isolated yield (%)	dr^a^

1^b^	**CF** **_3_**	PhCH_2_	3	92 (**4ba**)	87:13
2^b^	**CF** **_3_**	PhCH_2_	12	75 (**4ba**)	75:25
3^c^	**CF** **_3_**	PhCH_2_	3	80 (**4ba**)	61:39
4	**CF** **_3_**	PhCH_2_	5	90 (**4ba**)	94:6
5^d^	**CF** **_3_**	PhCH_2_	5	90 (**4ba**)	94:6
6	**CHF** **_2_**	PhCH_2_	48	76 (**4ca**)	>99:1
7	**CClF** **_2_**	PhCH_2_	24	87 (**4da**)	90:10
8	**C** **_2_** **F** **_5_**	PhCH_2_	81	72 (**4ea**)	69:31
9	**CF** **_3_**	CH_3_(CH_2_)_9_	10	59*^e^* (**4bb**)	95:5
10	**CF** **_3_**	Ph	5	92 (**4bc**)	93:7
11	**CF** **_3_**	CH_3_OC(O)CH_2_	5	94 (**4bd**)	95:5

^a^Determined by ^19^F NMR; ^b^reaction at 40 °C; ^c^reaction at 60 °C; ^d^utilization of 1.0 equiv of PhCH_2_SH resulted in the observation of 9% recovery of **2b** by ^19^F NMR; ^e^7% recovery of **2a** was observed by ^19^F NMR.

The different epoxyesters **2c**–**e** were also applied for this ring-opening reaction with the same thiol (entries 6–8 in Table 3). It is interesting to note that a longer reaction time was required for these substrates which would be the major reason for the relatively low diastereomeric ratio (especially in the case of entry 8 in Table 3) while the CHF_2_-possessing epoxyester **2b** furnished a single stereoisomer (entry 6) whose reason was not clear yet. Other thiols like decanethiol, thiophenol, and thioglycolate all worked nicely to furnish the corresponding products **4bb**–**bd** in good to excellent chemical yields with high stereoselectivities (Table 3, entries 9–11).

**Figure 1:**
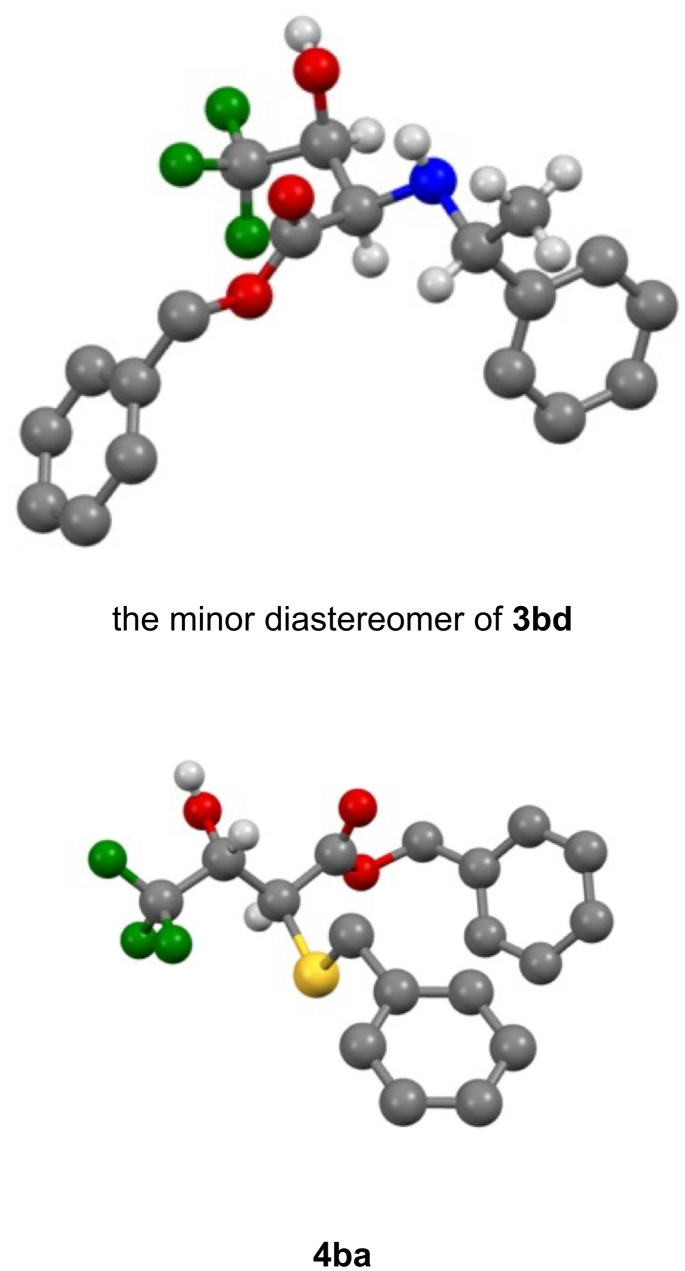
Crystallographic structure of the epoxy ring-opening products by PhCH(NH_2_)Me (**3bd**) and PhCH_2_SH (**4ba**).

The stereostructure of the products was confirmed by X-ray crystallographic analysis using the minor diastereomer of **3bd**, nicely separated from the major isomer by recrystallization, and the major product **4ba**. As was our expectation, these compounds [49] possess the *anti* relationship between the 2 and 3 positions which clearly proved the epoxy ring opening taking place at the 2 position in an S_N_2 fashion (Figure 1).

**Scheme 3:**
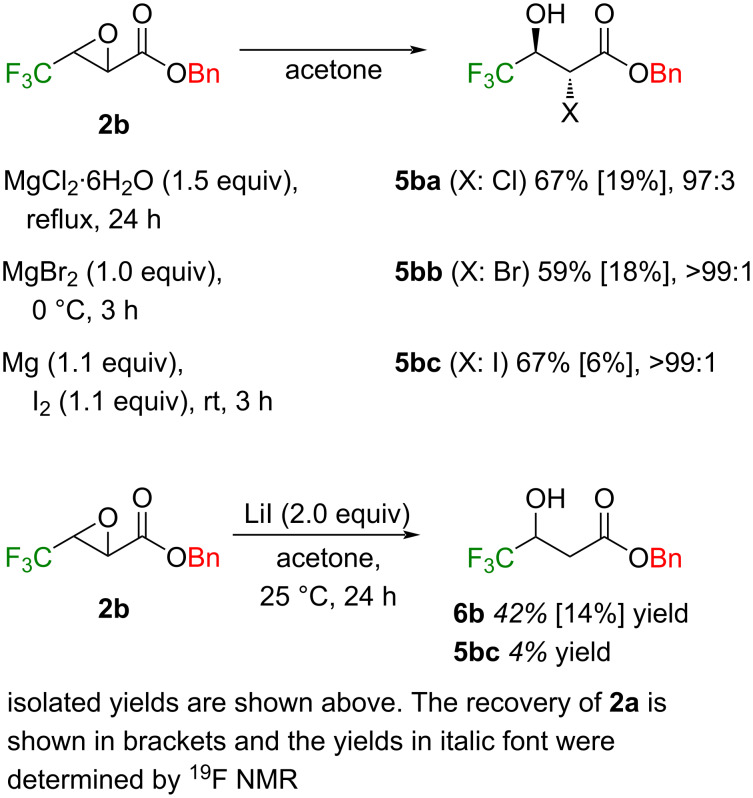
Introduction of additional halogen atoms at the 2-position of the compound **2b**.

The introduction of an additional halogen atom was considered to be possible by treatment of **2b** with an appropriate metal salt, and actually, similar results to the case of amines and thiols were obtained by using the corresponding MgX_2_ [23–24]. It was proved that a larger amount of nucleophiles, higher temperature, and longer time all led to a decrease in the diastereomeric ratio of the products **5** (ca 10%) like the case of thiols described above. This is the reason why the three examples shown in Scheme 3 stopped before completion, and, for example, 24 h stirring in the case of the Cl atom entry furnished 67% yield of **5ba** [32,34] and 19% recovery of **2b** with the diastereomeric ratio of the former of 97:3. Contamination by the deiodinated 3-hydroxyester [50] was noticed during the synthesis of **5bc** using LiI.

### Reactions of (*E*)-4,4,4-trifluoro-2,3-epoxybutanoate **2b** with compounds possessing an acidic proton

It was very interesting to know that there were scarce examples in the literature [51] on the ring opening of 2,3-epoxyesters in general by the stabilized anionic species from, for example, malonate. One reason could be because of the formation of the less stable alkoxide by the progress of the nucleophilic addition. If this is really the case, the presence of the strongly electron-withdrawing fluorine-containing groups in our instance should nicely affect the characteristics of the resultant intermediate which could lead to the realization of the addition of such nucleophilic species.

**Scheme 4:**
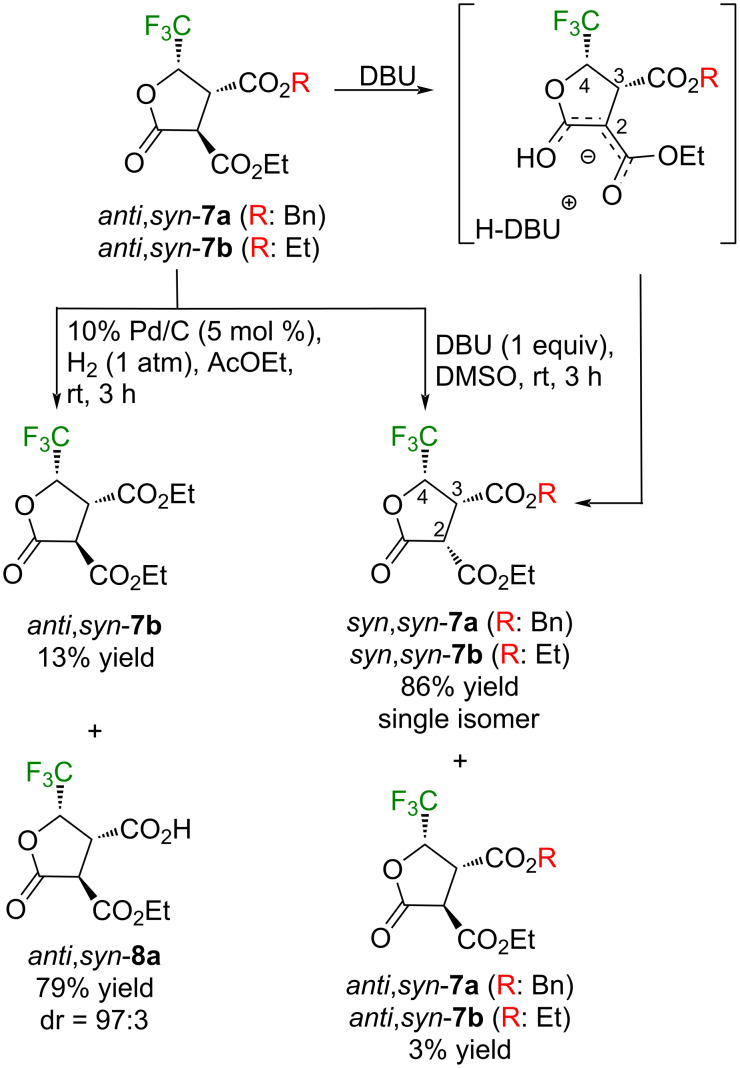
Clarification of the stereochemistry of *anti,syn*-**8a** and -**7b**.

**Figure 2:**
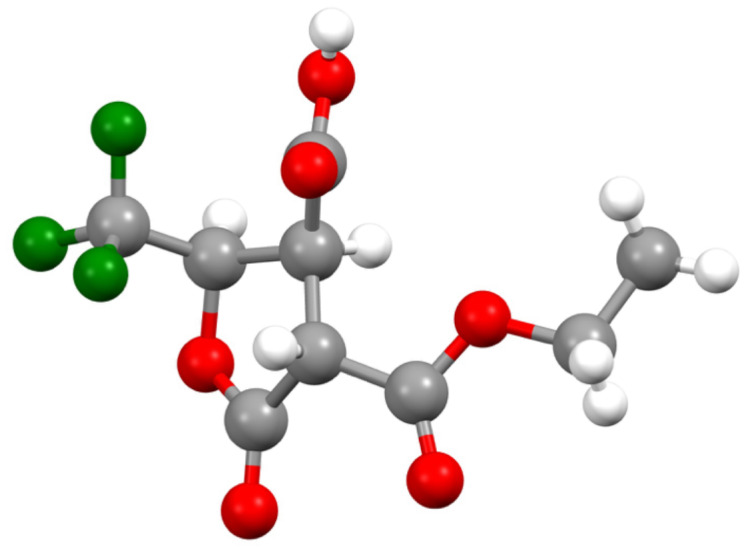
Crystallographic structure of *anti*,*syn*-**8a**.

First of all, as shown in Table 4, we started to investigate the reactivity of **2b** toward sodium malonate as the representative nucleophile. Because a brief solvent search indicated DMSO as the best for the attainment of high yields and diastereoselectivity (entries 1–5 vs 6 in Table 4), we further examined bases in this solvent to find out that *t*-BuOK behaved nicely, and the reaction of **2b** with 2.0 equiv of diethyl malonate for 0.5 h at room temperature furnished 93% yield of the product (Table 4, entry 15). During this optimization process, the obtained product was uncovered not to be a single component but a mixture of two compounds, *anti*,*syn*-**7a** and *anti*,*syn*-**7b**, the latter of which seemed to be produced from the former by the attack of the ethoxide ion released during the lactone-forming process. Their close structural resemblance led to a significant peak overlap both in the ^1^H and ^19^F NMR spectra which made it difficult to obtain their exact ratio and thus, the combined ^19^F NMR yields were shown in Table 4. Separation of these two compounds was eventually succeeded by the usual hydrogenolysis to furnish the carboxylic acid *anti*,*syn*-**8a** in 79% isolated yield and the lactone *anti*,*syn*-**7b** was recovered in 13% yield (Scheme 4) which was considered to be the reflection of the original composition of *anti*,*syn*-**7a** and -**7b**. The relative stereochemistry of *anti*,*syn*-**8a** was confirmed as 2,3-*anti*-3,4-*syn* by its X-ray crystallographic analysis [49] (Figure 2) whose construction could be readily understood as the result of a highly stereoselective S_N_2-type epoxy ring opening of **2a**, followed by the intramolecular lactone formation with the pro-*R* ethoxycarbonyl group possibly due to the higher steric congestion by the selection of the other CO_2_Et moiety.

**Table 4:** Reactions of **2b** with the anionic species from diethyl malonate.

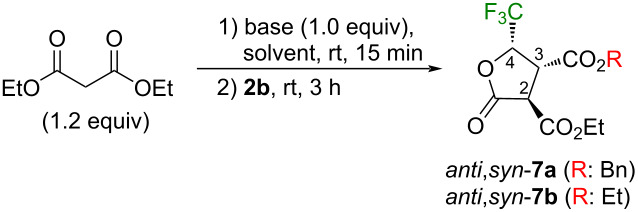					

Entry	Base	Solvent	Yield^a^ (%)	dr	Recovery (%)

1^b^	NaH	THF	20	>99:1	0
2^b^	NaH	Toluene	6	>99:1	13
3^b^	NaH	Et_2_O	12	>99:1	13
4^b^	NaH	MeCN	45	98:2	7
5^b^	NaH	DMF	75	96:4	0
6	NaH	DMSO	78	99:1	0
7	Et_3_N	DMSO	0	–	83
8	TMG	DMSO	22	14:86	3
9	DBU	DMSO	13	23:77	2
10	CsF	DMSO	34	91:9	21
11	K_2_CO_3_	DMSO	50	98:2	13
12	*t*-BuOK	DMSO	85	98:2	0
13^c^	*t*-BuOK	DMSO	91	99:1	0
14^d^	*t*-BuOK	DMSO	94	98:2	0
15^c,e^	*t*-BuOK	DMSO	93	99:1	0
16^b,c,e^	*t*-BuONa	DMF	56	98:2	5
17^b,c,e^	*t*-BuOLi	DMF	trace	–	32

^a^Combined yields of *anti*,*syn*-**7a** and -**7b** were determined by ^19^F NMR and isolated yield of *anti*,*syn*-**7a** was shown in parentheses; ^b^0 °C for 30 min were employed for the step 1 instead of rt, 15 min; ^c^2.0 equiv of malonate was used; ^d^3.0 equiv of malonate was used; ^e^stirring for 0.5 h for step 2.

As shown in entries 8 or 9 in Table 4, it was proved that the usage of tetramethylguanidine (TMG) or DBU as the base provided a different stereoisomer as the major component. For the confirmation of its stereostructure, the isolated inseparable mixture of *anti*,*syn*-**7a** and -**7b** by the reaction of **2b** and diethyl malonate was treated with an equimolar amount of DBU in DMSO (rt, 3 h) to furnish products which were identical to the ones obtained in entries 8 or 9 (Table 4). The relative stereochemistry of the isomerized products was concluded by the observed NOESY cross peaks between H^2^-H^4^ and H^3^-H^4^, clearly demonstrating the relationship between these three hydrogen atoms as *cis*. Formation of *syn*,*syn*-**7a** and -**7b** by the above tertiary amines would be mechanistically elucidated by the deprotonation of the most acidic H^2^ from the initially formed *anti*,*syn*-**7a** and -**7b**, followed by the re-protonation by the sterically bulky [H·amine]^+^ from the less congested top side.

**Scheme 5:**
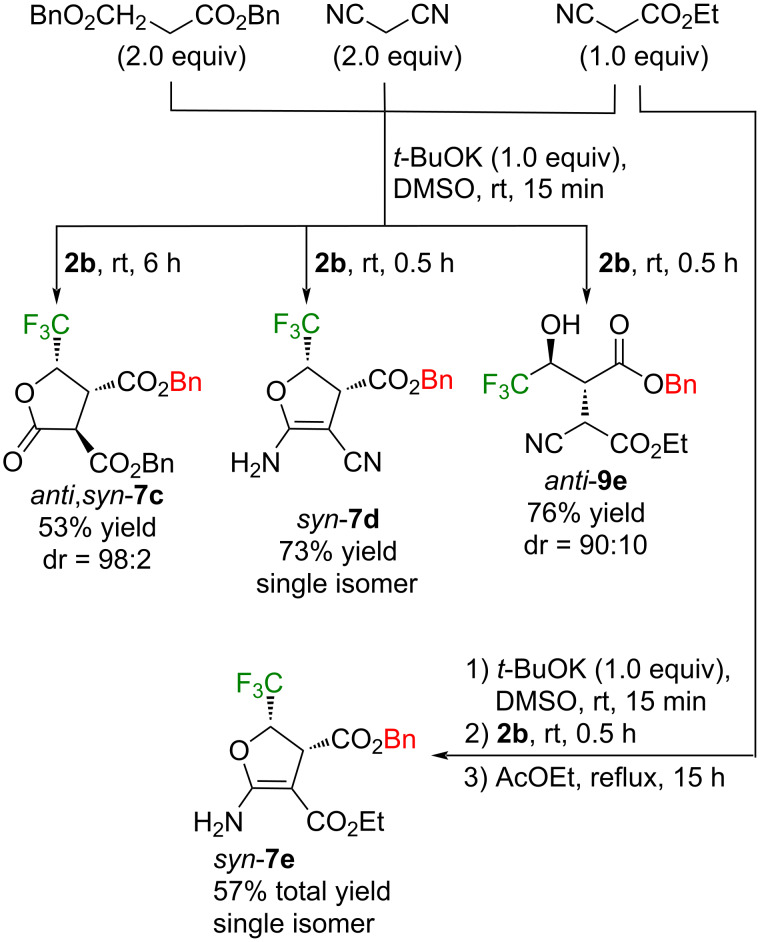
Reaction of **2b** with other stabilized nucleophiles.

These results prompted us to further investigate the ring opening of **2b** by other nucleophiles with active hydrogen whose results are summarized in Scheme 5. If the in situ conversion of *anti*,*syn*-**7a** to *anti*,*syn*-**7b** follows the above ester alcohol exchange mechanism, employment of dibenzyl malonate should afford a single compound. This is actually the case and the expected dihydrofuran *anti,syn*-**7c** was obtained in 53% yield as a 98:2 diastereomer mixture, and stereochemistry of the major isomer was deduced from the above result as *anti*,*syn*. Although malononitrile also furnished the dihydrofuran *syn*-**7d** in good yield as a sole stereoisomer, a sharp contrast to these results was observed when **2b** was subjected to the anionic species from cyanoacetate, allowing to isolate the acyclic hydroxyester *anti*-**9e** in 76% yield. Smooth conversion to the dihydrofuran *syn*-**7e** was observed from this intermediary compound *anti*-**9e** by refluxing the crude solution in AcOEt.

**Scheme 6:**
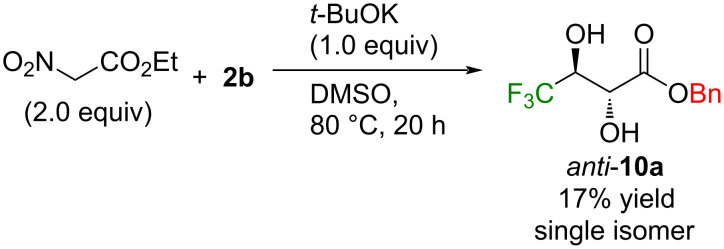
Production of 4,4,4-trifluoro-2,3-dihydroxybutanoate *anti*-**10a**.

Different from these outcomes, other possible nucleophilic candidates like acetylacetone (p*K*_a_ value of the active hydrogen in DMSO: 13.3 [52]), nitromethane (17.2 [53]), ethyl (diethylphosphono)acetate (18.6 [52]), malononitrile (11.1 [53]), ethyl 2-nitroacetate (9.1 [54]), ethyl 2-cyanoacetate (13.1 [55]), and diethyl malonate (16.4 [56]) all failed to afford the desired addition products. From the complex mixture after mixing **2b** with *t*-BuOK and nitroacetate in DMSO at 80 °C, the unexpected compound 2,3-dihydroxybutyrate *anti*-**10a** was isolated as a single isomer. Its production was also detected by ^19^F NMR from the reaction mixture when nitromethane (16%) and ethyl (diethylphosphono)acetate (17%) were employed instead of nitroacetate, while no other compound was separated from these mixtures due to their complexity (Scheme 6).

### Reactions of (*E*)-4,4,4-trifluoro-2,3-epoxybutanoate **2b** with Grignard-based copper reagents

Despite the previous report by the Seebach group on the intriguing reactivity of the CF_3_-containing ethyl 2,3-epoxybutanoate **2a** towards a variety of organometallic species [27–29], because relatively readily accessible Grignard-based cuprates were not involved, their applicability to **2b** as the representative partner was investigated here (Table 5).

**Table 5:** Optimization of the reaction conditions of **2b** with the *n*-C_10_H_21_MgBr-based cuprate.

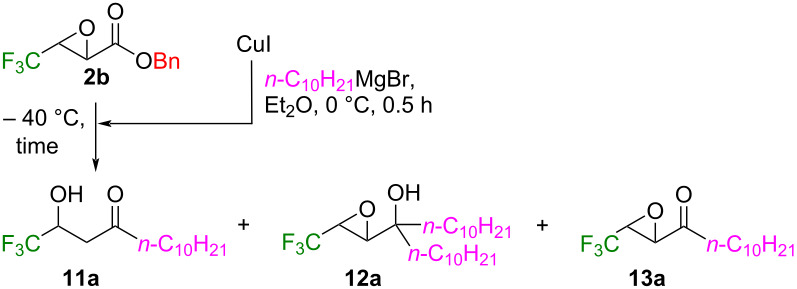					

	Amount (equiv)			^19^F NMR yield^a^ (%)	
			
Entry	CuI	*n*-C_10_H_21_MgBr	Time (h)	**11a**	**12a**

1^b^	2.0	2.0	1	47	4
2	2.0	4.0	1	30	40
3	2.0	4.0	4	64	12
4	1.6	3.2	4	66	16
5	1.2	2.4	4	49	19
6^c^	1.6	3.2	1	56	7
7	–	3.2	0.5	0	84 (73)
8^d^	1.6	3.2	3	3	13
9^e^	1.6	3.2	3	7	14
10^f^	1.6	3.2	3	(79)	6

^a^Isolated yields are described in parentheses; ^b^reaction at 0 °C when the cuprate was added to **2b**; ^c^19% of the epoxy ketone **13a** was detected by ^19^F NMR; ^d^the reaction was carried out in THF; ^e^CuCN was employed instead of CuI; ^f^the cuprate was prepared at −40 °C.

The 1:2 ratio of CuI and *n*-C_10_H_21_MgBr was selected due to the better material balance than the case of 1:1 (entries 1 and 2 in Table 5), the latter of which afforded an almost equimolar amount of the hydroxyketone **11a** and epoxyalcohol **12a**. A decrease of the temperature to −40 °C resulted in the better preference of **11a** (Table 5, entry 3), and 1.6 and 3.2 equiv of CuI and *n*-C_10_H_21_MgBr, respectively, were concluded as the best amounts for the synthesis of the nucleophilic species (entries 3–5). The shorter reaction time led to a slightly better ratio of **11a** to **12a** with a lower combined yield along with the detection of the epoxyketone **13a** at the same instance (entry 6 in Table 5). We recognized compound **13a** as the precursor for the formation of **11a** and **12a**. The conditions in the absence of CuI afforded **12a** as the sole product (entry 7 in Table 5) whose result was nicely compared with the one previously reported [29]. Changing the solvent to THF (Table 5, entry 8) or the Cu species to CuCN (entry 9) both did not have a positive effect on the present reaction, and we eventually found out that the temperature for the preparation of the cuprate was important and lowering it to −40 °C nicely allowed to record 79% isolated yield of **11a** with only 6% of the byproduct **12a** (entry 10).

The conditions described in entry 10 in Table 5 were applied to the reactions of **2b** with other Grignard reagents in the presence of CuI (Table 6).

**Table 6:** Reactions of Grignard-based cuprates with **2b**.

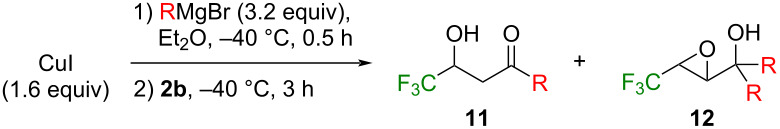		

	Isolated yield (%)	
	
R	**11**	**12**

*n*-C_10_H_21_- (**a**)	79	6^a^
PhCH_2_CH_2_- (**b**)	57	13^a^
*c*-Hex- (**c**)	55	4^a^
Ph- (**d**)	trace^a^	77
4-MeOC_6_H_4_- (e)	0^a^	80

^a^Determined by ^19^F NMR.

PhCH_2_CH_2_MgBr and *c*-C_6_H_11_MgBr produced the β-hydroxyketones **11b** and **11c** in 57% and 55% yields, respectively, along with small amounts of the corresponding epoxyalcohols **12b** and **12c**. On the other hand, **12d** and **12e** were substantially formed by ArMgBr (Ar: Ph and 4-MeOC_6_H_4_, respectively), the former of which was reported to be obtained by the action of PhLi alone [29]. It was intriguing to note that the present method yielded the unprecedented products **11** by the reaction of the epoxyester **2b** with other organometallic species.

**Scheme 7:**
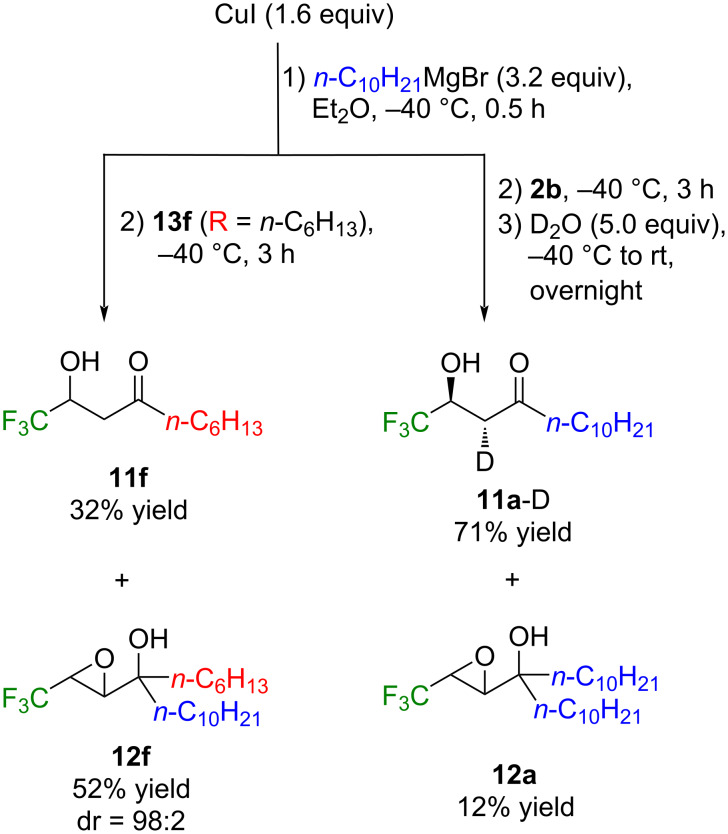
Reactions of *n*-C_10_H_21_MgBr-based cuprate with **13f** as well as **2b** with/without D_2_O quenching.

For the mechanistic clarification of the present reactions, two additional experiments were executed which are shown in Scheme 7. Employment of the epoxyketone **13f** (R: *n*-C_6_H_13_), structurally analogous to **13a**, to the reaction with (*n*-C_10_H_21_)_2_CuMgBr furnished a mixture of the hydroxyketone **11f** and epoxyalcohol **12f** in 32% and 52% yields, respectively. This experimental result clearly indicated that the conversion of **13a** to **11a** is one of the possible routes.

**Figure 3:**
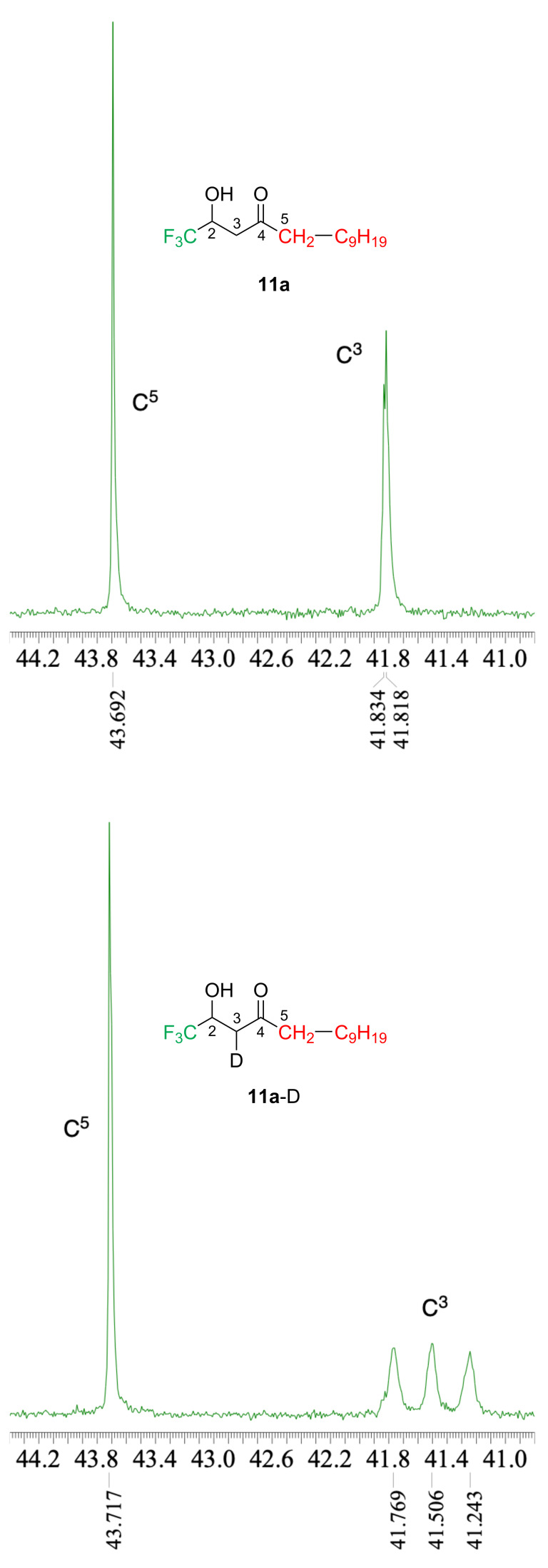
A part of ^13^C NMR spectra for the compounds **11a** and **11a**-D.

The second reaction was carried out for the verification of the intermediate leading to the product **11**. Although we initially assumed that the epoxy ring opening occurred by hydride generated through the β-elimination of the *n*-C_10_H_21_MgBr-based cuprate species, the TLC analysis of the reaction mixture did not show any evidence of the production of the possible olefinic product *n*-C_8_H_17_CH=CH_2_. Moreover, when the reaction mixture was quenched with D_2_O, incorporation of deuterium was observed to give **11a**-D in a high yield which allowed us to conclude the possible presence of the *C*-copper species just before quenching. Our result well compares with the one by Alexakis et al. [57]*.* In their instance, the reaction of *t*-Bu_2_CuCNLi_2_ and cyclohexene oxide afforded a mixture of products in 10 and 50% yields as a result of the epoxy ring opening by *t*-Bu group and hydride, respectively. Their additional experiment to quench the corresponding intermediate by D_2_O proved that no deuteration occurred. This result clearly indicated that hydride was released from the *t*-Bu group of the Cu(III) species formed after the nucleophilic attack of the epoxy ring. In our case, since the strongly electron-withdrawing CF_3_ group would render the rate of the reductive elimination very slow, the intermediary Cu(III) species safely existed until the addition of D_2_O. Because the significant overlap of NMR peaks was observed due to the quite similar structure of **11a** and **11a**-D, quantitative analysis of the deuterium content of **11a**-D was not possible. However, the comparison of their specific region of the ^13^C NMR charts and sharp peaks readily led us to qualitative understanding of the high purity of **11a**-D possibly as a single diastereomer (Figure 3).

## Conclusion

As described above, we have succeeded in the facile preparation of 2,3-epoxyesters **2** with a variety of Rf groups at the 3 position starting from the corresponding 3-Rf-acrylates **1** by the action of the low cost and easy-to-handle reagent, NaOCl·5H_2_O. The special feature of this process is the requirement of a very mild temperature of 0 °C which can be well compared to the previous one executed at −78 °C under the action of LDA [29]. Moreover, by using the epoxyester **2b** as the representative substrate, clarification of its reactivity was carried out by mixing with 1) heteronucleophiles like amines, thiols, and magnesium halides, 2) softer carbon nucleophiles such as malonates, and 3) Grignard-based cuprates. These processes usually yielded the addition products along with the epoxy ring opening at the 2 position via the S_N_2 mechanism, affording 3-Rf-3-hydroxyesters with the incorporation of a variety of substituents at the 2-position in a highly *anti*-selective fashion. We believe that the facile procedure presented here opens novel routes to the application of these intriguing products in a variety of fields.

## Experimental

### General procedure for the formation of the epoxyesters (GP-1): Benzyl (*E*)-2,3-epoxy-4,4,4-trifluorobutanoate (**2b**)

**GP-1A** (by use of aqueous NaClO): To a solution of compound **1b** [42] (0.23 g, 1.00 mmol) in 3.0 mL of CH_3_CN was added NaClO aq. (5% in H_2_O, 1.50 g, 1.00 mmol) and the solution was stirred for 4.5 h at room temperature. This mixture was extracted with CH_2_Cl_2_ and the usual workup and purification afforded 0.15 g (0.60 mmol) of the pure title compound in 60% yield.

**GP-1B** (by use of NaClO·5H_2_O): To a solution of compound **1b** [42] (0.2302 g, 1.00 mmol) in 3.0 mL of CH_3_CN was added NaClO·5H_2_O (0.3290 g, 2.00 mmol) at 0 °C, and the solution was stirred for 6 h at the same temperature. After the same workup process and purification with silica gel column chromatography using AcOEt/Hex 1:20 as an eluent, 0.2117 g (0.86 mmol) of the title compound (86% yield) were isolated. *R*_f_ 0.52 (Hex/AcOEt 5:1); ^1^H NMR (300.40 MHz, CDCl_3_) δ 3.71–3.76 (m, 2H), 5.21 (d, *J* = 12.3 Hz, 1H), 5.28 (d, *J* = 12.3 Hz, 1H), 7.34–7.44 (m, 5H); ^13^C NMR (75.45 MHz, CDCl_3_) δ 49.4 (q, *J* = 2.5 Hz), 52.7 (q, *J* = 42.2 Hz), 68.0, 121.4 (q, *J* = 276.0 Hz), 128.5, 128.7, 128.8, 134.3, 165.6; ^19^F NMR (282.65 MHz, CDCl_3_) δ −75.12 (d, *J* = 4.5 Hz); IR (neat) ν: 3944, 3689, 3054, 2987, 2685, 2306, 1756, 1456, 1422, 1382, 1341, 1265, 1169, 1089, 988, 929, 896, 664 cm^−1^; Anal. calcd for C_11_H_9_F_3_O_3_: C, 53.67; H, 3.68; found: C, 53.54; H, 3.89.

### General procedure for the ring opening of epoxides (GP-2). Benzyl 2,3-*anti*-4,4,4-trifluoro-3-hydroxy-2-(*p*-methoxyphenyl)amino-butanoate (**3ba**)

*p*-Anisidine (0.07 g, 0.60 mmol) was added to an EtOH (3 mL) solution of compound **2b** (0.12 g, 0.50 mmol), and the resultant mixture was stirred at 50 °C for 19 h under the open air. After quenching the reaction with 1 M HCl aq., the mixture was extracted with AcOEt three times and the combined organic phase was washed with brine. Evaporation of the volatiles furnished crude materials which were recrystallized by use of Hex/CHCl_3_ 3:2 as a solvent to afford 0.14 g (0.39 mmol) of the title compound **3aa** in 78% yield as a sole stereoisomer. *R*_f_ 0.30 (Hex/AcOEt 2:1); mp 95–97 °C; ^1^H NMR (300.40 MHz, CDCl_3_) δ 3.70 (brs, 1H), 3.76 (s, 3H), 4.31–4.33 (m, 2H), 4.39 (brs, 1H), 5.14 (dd, *J* = 12.0, 21.3 Hz, 1H), 6.74–6.81 (m, 4H), 7.26–7.36 (m, 5H); ^13^C NMR (75.45 MHz, acetone-*d_6_*) δ 55.5, 59.3, 67.9, 70.0 (q, *J* = 30.2 Hz), 114.8, 117.7, 124.1 (q, *J* = 283.5 Hz), 128.5, 128.6, 128.7, 134.4, 139.5, 154.3, 170.2; ^19^F NMR (282.65 MHz, CDCl_3_) δ −76.83 (d, *J* = 9.0 Hz); IR (KBr) ν: 3454, 3315, 2955, 2924, 2854, 2360, 1741, 1519, 1458, 1238, 1204, 1156, 1138, 1097, 1030, 822, 749 cm^−1^; HRMS–FAB (*m/z*): [M]^+^ calcd for C_18_H_18_F_3_NO_4_, 369.1182; found, 369.1209.

### General procedure for the ring opening of epoxides by enolates (GP-3). 4-Benzyl 5-ethyl *anti*,*syn*-tetrahydro-2-oxo-3-(trifluoromethyl)-furan-4,5-dicarboxylate (*anti*,*syn*-**7a**) and 4,5-diethyl *anti*,*syn*-tetrahydro-2-oxo-3-(trifluoromethyl)furan-4,5-dicarboxylate (*anti*,*syn*-**7b**)

Diethyl malonate (0.18 mL, 1.20 mmol) was added to a flask containing 0.0673 g (0.60 mmol) of *t*-BuOK in DMSO (1.8 mL) under an argon atmosphere and the resultant mixture was stirred for 15 min at room temperature. Then, 0.1477 g (0.60 mmol) of **2b** in 0.8 mL of DMSO was introduced to the resultant solution and the stirring was continued for 0.5 h. The same workup process and purification furnished 0.1717 g of an inseparable mixture of *anti*,*syn*-**7a** (dr = 99:1) and *anti*,*syn*-**7b** (**7a**:**7b** = 83:17). *Anti*,*syn*-**7a**: *R*_f_ 0.34 (Hex/AcOEt 4:1); ^1^H NMR (300.40 MHz, CDCl_3_) δ 1.32 (t, *J* = 7.2 Hz, 3H), 4.20–4.27 (m, 2H), 4.21–4.35 (m, 2H), 5.05 (quint, *J* = 7.2 Hz, 1H), 5.15 (d, *J* = 12.3 Hz, 1H), 5.23 (d, *J* = 12.0 Hz, 1H), 7.32–7.40 (m, 5H); ^13^C NMR (75.45 MHz, CDCl_3_) δ 13.8, 44.4, 46.4, 63.1, 68.5, 73.5 (q, *J* = 34.1 Hz), 122.5 (q, *J* = 282.9 Hz), 128.61, 128.63, 128.8, 134.0, 165.1, 165.6, 167.4; ^19^F NMR (282.65 MHz, CDCl_3_) δ −75.84 (d, *J* = 6.8 Hz); IR (neat) ν: 2987, 1813, 1742, 1457, 1389, 1321,1218, 1182, 1128, 1023, 972, 755 cm^−1^; HRMS–FAB+ (*m/z*): [M + H]^+^ calcd for C_16_H_16_F_3_O_6_, 361.0893; found, 361.0911. Epimer at the 2 position of *anti*,*syn*-**7a** (*syn*,*syn*-**7a**): ^1^H NMR (300.40 MHz, CDCl_3_) δ 1.30 (t, *J* = 7.2 Hz, 3H), 4.00 (d, *J* = 8.4 Hz, 1H), 4.08 (dd, *J* = 6.3, 8.1 Hz, 1H), 4.26–4.34 (m, 2H), 5.00 (quint, *J* = 5.7 Hz, 1H), 5.22 (d, *J* = 12.0 Hz, 1H), 5.27 (d, *J* = 12.3 Hz, 1H), 7.31–7.41 (m, 5H); ^13^C NMR (75.45 MHz, CDCl_3_) δ 13.9, 43.2, 48.5, 63.3, 68.6, 74.9 (q, *J* = 35.4 Hz), 122.4 (q, *J* = 279.8 Hz), 128.3, 128.8, 128.9, 134.2, 164.5, 167.1, 168.2; ^19^F NMR (282.65 Hz, CDCl_3_) δ −79.55 (d, *J* = 4.8 Hz); HRMS–FAB+ (*m/z*): [M + H]^+^ calcd for C_16_H_16_F_3_O_6_, 361.0893; found, 361.0909.

### General procedure for the reaction of the epoxyester **2b** with cuprates (GP-4): 1,1,1-Trifluoro-2-hydroxytetradecan-4-one (**11a**)

1.70 mL of a 0.94 M Et_2_O solution of decylmagnesium bromide (1.6 mmol) was added to an Et_2_O (3.0 mL) solution containing 0.1524 g (0.80 mmol) of CuI at −40 °C under an argon atmosphere and the resultant mixture was stirred for 0.5 h at that temperature. A solution of 0.1231 g (0.50 mmol) of **2b** in Et_2_O (1.0 mL) was added and the mixture was stirred for 3 h at the same temperature. After quenching the reaction with a saturated NH_4_Cl aq, the usual workup afforded 0.1116 g (0.40 mmol) of the title compound in 79% yield after silica gel column chromatography using Hex/AcOEt 6:1 as an eluent. *R*_f_ 0.51 (Hex/AcOEt 4:1); ^1^H NMR (300.40 MHz, CDCl_3_) δ 0.88 (t, *J* = 6.9 Hz, 3H), 1.26 (brs, 14H), 1.60 (quint, *J* = 6.9 Hz, 2H), 2.49 (t, *J* = 7.5 Hz, 2H), 2.74 (dd, *J* = 3.6, 17.7 Hz, 1H), 2.83 (dd, *J* = 9.0, 17.7 Hz, 1H), 3.49 (d, *J* = 4.2 Hz, 1H), 4.43–4.56 (m, 1H); ^13^C NMR (75.45 MHz, CDCl_3_) δ 14.0, 15.0, 22.6, 23.4, 29.26, 29.28, 29.4, 29.5, 31.8, 41.8 (q, *J* = 1.2 Hz), 43.7, 66.4 (q, *J* = 32.2 Hz), 124.7 (q, *J* = 281.1 Hz), 208.9; ^19^F NMR (282.65 MHz, CDCl_3_) δ −80.79 (d, *J* = 7.1 Hz); IR (neat) ν: 3408, 2958, 2927, 2856, 1720, 1469, 1291, 1176, 1146, 899, 841, 719, 643 cm^−1^; HRMS–FAB+ (*m/z*): [M + H]^+^ calcd for C_14_H_26_F_3_O_2_, 283.1879; found, 283.1893.

## Supporting Information

File 1 Full experimental and analytical details, copies of NMR spectra for new compounds, and crystallographic data.

## Data Availability

All data that supports the findings of this study is available in the published article and/or the supporting information to this article.
