# Supplementary material for: Facile preparation of fluorine-containing 2,3-epoxypropanoates and their epoxy ring-opening reactions with various nucleophiles
Source: Beilstein J Org Chem. 2024 Sep 25;20:2421–33. doi: 10.3762/bjoc.20.206 (PMC11443650; doi:10.3762/bjoc.20.206)
Supplement: File 1 — Full experimental and analytical details, copies of NMR spectra for new compounds, and crystallographic data. [file Beilstein_J_Org_Chem-20-2421-s001.pdf]

BEILSTEIN JOURNAL OF ORGANIC CHEMISTRY

Supporting Information

for

Facile preparation of fluorine-containing 2,3-epoxypropanoates and their epoxy ring-opening reactions with various nucleophiles

Yutaro Miyashita, Sae Someya, Tomoko Kawasaki-Takasuka, Tomohiro Agou and Takashi Yamazaki

Beilstein J. Org. Chem. **2024,** *20,* 2421–2433. doi:10.3762/bjoc.20.206

Full experimental and analytical details, copies of NMR spectra for new compounds, and crystallographic data

License and Terms: This is a supporting information file under the terms of the Creative Commons Attribution License (https://creativecommons.org/licenses/by/4.0). Please note that the reuse, redistribution and reproduction in particular requires that the author(s) and source are credited and that individual graphics may be subject to special legal provisions.
The license is subject to the *Beilstein Journal of Organic Chemistry* terms and conditions: (https://www.beilstein-journals.org/bjoc/terms)

Experimental procedures

General information S1
Formation of benzyl (*E*)-4-chloro-4,4-difluorobut-2-enoate (**1d**).......... S1

General procedure for the formation of the epoxy esters (GP-1)

Benzyl (*E*)-2,3-epoxy-4,4,4-trifluorobutanoate (**2b**) S2
Benzyl (*E*)-2,3-epoxy-4,4-difluorobutanoate (**2c**).......... S2
Benzyl (*E*)-4-chloro-2,3-epoxy-4,4-difluorobutanoate (**2d**) S2
Benzyl (*E*)-2,3-epoxy-4,4,5,5,5-pentafluoropentanoate (**2e**) S3
Ethyl (*E*)-2,3-epoxy-4,4,5,5,6,6,7,7,8,8,9,9,9-tridercafluorononanonate (**2f**).......... S3

General Procedure for the ring opening of epoxides (GP-2)

Benzyl 2,3-*anti*-4,4,4-trifluoro-3-hydroxy-2-(*p*-methoxyphenyl)aminobutanoate (**3ba**)· S3
Benzyl 2,3-*anti*-4,4-difluoro-3-hydroxy-2-(*p*-methoxyphenyl)aminobutanoate (**3ca**).......... S4
Benzyl 2,3-*anti*-4-chloro-4,4-difluoro-3-hydroxy-2-(*p*-methoxyphenyl)amino-butanoate (**3da**.......... S4
Benzyl 2,3-*anti*-2-(benzylamino)-4,4,4-trifluoro-3-hydroxybutanoate (**3bb**.......... S4
Benzyl 2,3-*anti*-2-*n*-buthylamino-4,4,4-trifluoro-3-hydroxybutanoate (**3bc**).......... S5
Benzyl 2,3-*anti*-2-(1-phenylethylamino)-4,4,4-trifluoro-3-hydroxybutanoate (**3bd**) S5
Benzyl (2*R**,3*S**)-2,3-*anti*-2-((2'*R**)-1-phenylethylamino)-4,4,4-trifluoro-3-hydroxy-butanoate ((**2*R**,3*S**,2'*R**)-3bd**, minor isomer) S5
Benzyl 2,3-*anti*-2-[{(*S*)-1-(benzyloxycarbonyl)-2-methylbutyl}amino]-4,4,4-trifluoro-3-hydroxybutanoate (**3be**) S5
Benzyl 2,3-*anti*-2-(*N*,*N*-diethylamino)-4,4,4-trifluoro-3-hydroxybutanoate (**3bf**) S6
Benzyl 2,3-*anti*-2-(pyrrolidin-1-yl)-4,4,4-trifluoro-3-hydroxybutanoate (**3bg**) S6
Benzyl 2,3-*anti*-4,4,4-trifluoro-3-hydroxy-2-(phenylmethylsulfenyl)butanoate (**4ba**) S6
Benzyl 2,3-*anti*-4,4-difluoro-3-hydroxy-2-(phenylmethylsulfenyl)butanoate (**4ca**) S7
Benzyl 2,3-*anti*-4-chloro-4,4-difluoro-3-hydroxy-2-(phenylmethyl-sulfenyl)butanoate (**4da**) S7
Benzyl 2,3-*anti*-4,4,5,5,5-pentafluoro-3-hydroxy-2-(phenylmethylsulfenyl)-butanoate (**4ea**) S8
Benzyl 2,3-*anti*-2-(decylsulfenyl)-4,4,4-trifluoro-3-hydroxybutanoate (**4bb**) S8
Benzyl 2,3-*anti*-4,4,4-trifluoro-3-hydroxy-2-(phenylsulfenyl)butanoate (**4bc**) S8
Benzyl 2,3-*anti*-4,4,4-trifluoro-3-hydroxy-2-{(methoxycarbonyl)methyl-sulfenyl)}butanoate (**4bd**) S9
Benzyl 2,3-*anti*-2-chloro-4,4,4-trifluoro-3-hydroxybutanoate (**5ba**) S9
Benzyl 2,3-*anti*-2-bromo-4,4,4-trifluoro-3-hydroxybutanoate (**5bb**) S9
Benzyl 2,3-*anti*-4,4,4-trifluoro-3-hydroxy-2-iodobutanoate (**5bc**) S10
Benzyl 4,4,4-trifluoro-3-hydroxybutanoate (**6b**) S10

General Procedure for the ring opening of epoxides by enolates (GP-3)

4-Benzyl 3-ethyl *anti*,*syn*-tetrahydro-2-oxo-4-(trifluoromethyl)furan-dicarboxylate (***anti*,*syn*-7a**) S10
3,4-Diethyl *anti*,*syn*-tetrahydro-2-oxo-4-(trifluoromethyl)furan-dicarboxylate (***anti*,*syn*-7b**) S10
3-Ethyl *anti*,*syn*-tetrahydro-2-oxo-4-(trifluoromethyl)furandicarboxylate (***anti*,*syn*-8a**) S11
3,4-Diethyl *anti*,*syn*-tetrahydro-2-oxo-4-(trifluoromethyl)furan-dicarboxylate (***anti*,*syn*-7b**) S11
3,4-Dibenzyl *anti*,*syn*-tetrahydro-2-oxo-4-(trifluoromethyl)furan-dicarboxylate (***anti*,*syn*-7c**) S12
Benzyl *anti*-5-amino-4-cyano-2-(trifluoromethyl)-2,3-dihydrofuran-3-carboxylate (***syn*-7d**) S12
3-Benzyl-4-ethyl *anti*-5-amino-2-(trifluoromethyl)-2,3-dihydrofuran-dicarboxylate (***syn*-7e**) S12
Benzyl 2,3-*anti*-2-{cyano(ethoxycarbonyl)methyl}-4,4,4-trifluoro-3-hydroxy-butanoate (***anti*-9e**) S13
Benzyl 2,3-*anti*-4,4,4-trifluoro-2,3-dihydroxybutanoate (***anti*-10a**) S13

General procedure for the reaction of the epoxyester 3a with cuprates (GP-4)

1,1,1-Trifluoro-2-hydroxytetradecan-4-one (**11a**) S13
6,6,6-Trifluoro-5-hydroxy-1-phenylhexan-3-one (**11b**) S14
1-Cyclohexyl-4,4,4-trifluoro-3-hydroxybutan-1-one (**11c**) S14
(*E*)-4-Decyl-2,3-epoxy-4,4,4-trifluorotetradecan-4-ol (**12a**) S14
(*E*)-2,3-Epoxy-4,4,4-trifluoro-1,1-diphenylbutan-1-ol (**12d**)........ S14
(*E*)-2,3-Epoxy-4,4,4-trifluoro-1,1-bis(4-methoxyphenyl)butan-1-ol (**12e**) S15
1,1,1-Trifluoro-2-hydroxydecan-4-one (**11f**) S15
2,3-Epoxy-1,1,1-trifluoro-4-hexyltetradecan-4-ol (**12f**) S15
1,1,1-Trifluoro-3-deutero-2-hydroxytetradecan-4-one (**11a-D**)........ S15
(*E*)-2,3-Epoxy-4,4,4-trifluorotetradecan-4-one (**13a**) S16

^{1}H and ^{13}C NMR charts for new compounds

Benzyl (*E*)-4-chloro-4,4-difluorobut-2-enoate (**1d**)........ S17
Benzyl (*E*)-2,3-epoxy-4,4,4-trifluorobutanoate (**2b**)........ S18
Benzyl (*E*)-2,3-epoxy-4,4-difluorobutanoate (**2c**)........ S19
Benzyl (*E*)-4-chloro-2,3-epoxy-4,4-difluorobutanoate (**2d**) S20
Benzyl (*E*)-2,3-epoxy-4,4,5,5,5-pentafluoropentanoate (**2e**) S21
Ethyl (*E*)-2,3-epoxy-4,4,5,5,6,6,7,7,8,8,9,9,9-tridercafluorononanonate (**2f**)........ S22
Benzyl 2,3-*anti*-4,4,4-trifluoro-3-hydroxy-2-(*p*-methoxyphenyl)aminobutanoate (**3ba**) S23
Benzyl 2,3-*anti*-4,4-difluoro-3-hydroxy-2-(*p*-methoxyphenyl)aminobutanoate (**3ca**) S24
Benzyl 2,3-*anti*-4-chloro-4,4-difluoro-3-hydroxy-2-(*p*-methoxyphenyl)-aminobutanoate (**3da**) S25
Benzyl 2,3-*anti*-2-(benzylamino)-4,4,4-trifluoro-3-hydroxybutanoate (**3bb**) S26
Benzyl 2,3-*anti*-2-(*n*-buthylamino)-4,4,4-trifluoro-3-hydroxybutanoate (**3bc**) S27
Benzyl 2,3-*anti*-2-(1-phenylethylamino)-4,4,4-trifluoro-3-hydroxybutanoate (**3bd**)........ S28
Benzyl (2*R**,3*S**)-2,3-*anti*-2-[(1*R**)-1-phenylethylamino]-4,4,4-trifluoro-3-hydroxybutanoate (**(2*R**,3*S**,2’*R**)-3bd**)........ S29

Benzyl 2,3-*anti*-2-[{(*S*)-1-(benzyloxycarbonyl)-2-methylbutyl}amino]-4,4,4-trifluoro-3-hydroxybutanoate (**3be**) S30
Benzyl 2,3-*anti*-2-(*N*,*N*-diethylamino)-4,4,4-trifluoro-3-hydroxybutanoate (**3bf**) S31
Benzyl 2,3-*anti*-2-(pyrrolidin-1-yl)-4,4,4-trifluoro-3-hydroxybutanoate (**3ag**) S32
Benzyl 2,3-*anti*-4,4,4-trifluoro-3-hydroxy-2-(phenylmethylsulfenyl)butanoate (**4ba**) S33
Benzyl 2,3-*anti*-4,4-difluoro-3-hydroxy-2-(phenylmethylsulfenyl)butanoate (**4ca**) S34
Benzyl 2,3-*anti*-4-chloro-4,4-difluoro-3-hydroxy-2-(phenylmethyl-sulfenyl)butanoate (**4da**) S35
Benzyl 2,3-*anti*-4,4,5,5,5-pentafluoro-3-hydroxy-2-(phenylmethyl-sulfenyl)butanoate (**4ea**) S36
Benzyl 2,3-*anti*-2-(*n*-decylsulfenyl)-4,4,4-trifluoro-3-hydroxybutanoate (**4bb**) S37
Benzyl 2,3-*anti*-4,4,4-trifluoro-3-hydroxy-2-(phenylsulfenyl)butanoate (**4bc**) S38
Benzyl 2,3-*anti*-4,4,4-trifluoro-3-hydroxy-2-{(methoxycarbonyl)methylsulfenyl)}-butanoate (**4bd**) S39
Benzyl 2,3-*anti*-2-chloro-4,4,4-trifluoro-3-hydroxybutanoate (**5ba**) S40
Benzyl 2,3-*anti*-2-bromo-4,4,4-trifluoro-3-hydroxybutanoate (**5bb**) S41
Benzyl 2,3-*anti*-4,4,4-trifluoro-3-hydroxy-2-iodobutanoate (**5bc**) S42
4-Benzyl 3-ethyl *anti*,*syn*-tetrahydro-2-oxo-4-(trifluoromethyl)furan-dicarboxylate (***anti*,*syn*-7a**) S43
3,4-Diethyl *anti*,*syn*-tetrahydro-2-oxo-4-(trifluoromethyl)furan-dicarboxylate (***anti*,*syn*-7b**) S44
4-Benzyl 3-ethyl *syn*,*syn*-tetrahydro-2-oxo-4-(trifluoromethyl)furan-dicarboxylate (***syn*,*syn*-7a**) S45
3,4-Dibenzyl *anti*,*syn*-tetrahydro-2-oxo-4-(trifluoromethyl)furan-dicarboxylate (***anti*,*syn*-7c**) S46
Benzyl *anti*-5-amino-4-cyano-2-(trifluoromethyl)-2,3-dihydrofuran-3-carboxylate (***syn*-7d**) S47
3-Benzyl-4-ethyl *anti*-5-amino-2-(trifluoromethyl)-2,3-dihydrofuran-dicarboxylate (***syn*-7e**) S48
3-Ethyl *anti*,*syn*-tetrahydro-2-oxo-4-(trifluoromethyl)furandicarboxylate (***anti*,*syn*-8a**) S49
Benzyl 2,3-*anti*-2-{cyano(ethoxycarbonyl)methyl}-4,4,4-trifluoro-3-hydroxybutanoate (***anti*-9e**) S50
Benzyl 2,3-*anti*-4,4,4-trifluoro-2,3-dihydroxybutanoate (***anti*-10a**) S51
1,1,1-Trifluoro-2-hydroxytetradecan-4-one (**11a**) S52
(*E*)-4-Decyl-2,3-epoxy-4,4,4-trifluorotetradecan-4-ol (**12a**) S53
(*E*)-2,3-Epoxy-4,4,4-trifluoro-1,1-bis(4-methoxyphenyl)butan-1-ol (**12e**) S54
2,3-Epoxy-1,1,1-trifluoro-4-*n*-hexyltetradecan-4-ol (**12f**) S55
1,1,1-Trifluoro-3-deutero-2-hydroxytetradecan-4-one (**11a-D**) S56
(*E*)-2,3-Epoxy-4,4,4-trifluorotetradecan-4-one (**13a**) S57

Crystallographic data

Benzyl (2*R**,3*S**)-2,3-*anti*-2-((2’*R**)-1-phenylethylamino)-4,4,4-trifluoro-3-hydroxy-butanoate (**(2*R**,3*S**,2’*R**)-3bd**, minor isomer) S58
Benzyl 2,3-*anti*-4,4,4-trifluoro-3-hydroxy-2-(phenylmethylsulfenyl)butanoate (**4ba**) S59
3-Ethyl *anti*,*syn*-tetrahydro-2-oxo-4-(trifluoromethyl)furandicarboxylate (***anti*,*syn*-8a** S60

References S61

General information. ^{1}H (300.40 MHz), ^{13}C (75.45 MHz), and ^{19}F (282.65 MHz) NMR spectra were recorded on a JEOL AL 300 spectrometer and chemical shifts were recorded in parts per million (ppm), downfield from internal tetramethylsilane (Me_4Si: δ 0.00, for ^{1}H and ^{13}C) or hexafluorobenzene (C_6F_6: δ −163.00 for ^{19}F). Data were tabulated in the following order: number of protons, multiplicity (s, singlet; d, doublet; t, triplet; q, quartet; quint, quintet; sex, sextet; m, multiplet; b, broad peak), coupling constants in hertz. Infrared (IR) spectra were obtained on a JASCO A-302 spectrometer and reported in wave numbers (cm^{-1}). Elemental analyses were performed by Perkin-Elmer Series II CHNS/O analyzer. JEOL JMS-700 was used for obtaining high resolution mass spectrometry data by the positive ionization mode.

Most of the reactions where an organic solvent was employed were performed under argon with magnetic stirring using flame-dried glassware. Anhydrous THF, Et_2O, and CH_2Cl_2 were purchased and used without further purification. Unless otherwise noted, materials were obtained from commercial suppliers and were used without further purification. Analytical thin-layer chromatography (TLC) was routinely used for monitoring reactions by generally using a mixture of hexane (Hex) and ethyl acetate (AcOEt) (v/v). Spherical neutral silica gel (63–210 μm or 40–50 μm) was employed for column chromatography and flush chromatography, respectively.

Formation of benzyl (*E*)-4-chloro-4,4-difluorobut-2-enoate (1d). To a three-necked round-bottomed flask containing THF (20 mL) and ethyl chlorodifluoroacetate (1.50 mL, 12.0 mmol) was added diisobutylaluminium hydride (1.0 M in hexanes, 12.0 mL, 12.0 mmol) at −80 °C and the mixture was stirred for 0.5 h at that temperature.

Benzyl diethylphosphonoacetate [1] (1.43 g, 10.0 mmol) was added to a THF (10.0 mL) solution of lithium bromide (1.0424 g, 12.0 mmol) in a separate flask at 0 °C where triethylamine (1.54 mL, 11.0 mmol) was added and the whole solution was stirred for 10 min at room temperature. An aluminum acetal solution prepared above was slowly introduced to this mixture at −80 °C with the aid of a cannula, and the resultant mixture was further stirred for 5 h at room temperature. After quenching this reaction, the crude material was extracted with AcOEt three times, and the combined organic phase was washed with brine and dried over anhydrous Na_2SO_4. After filtration, the removal of the volatiles furnished the crude materials which were purified by silica gel column chromatography (Hex:CH_2Cl_2 = 6:1) to afford 1.080 g (4.4 mmol, 44% yield, >99% *E* selectivity) of the title compound **1c**. Rf = 0.61 (Hex:AcOEt = 4:1). ^{1}H NMR δ 5.24 (2H, s), 6.43 (1H, td, *J* = 1.8, 15.6 Hz), 6.96 (1H, td, *J* = 9.0, 15.3 Hz), 7.37-7.41 (5H, m). ^{13}C NMR (75.45 MHz, $CDCl_3$): δ 67.3, 123.5 (t, *J* = 287.2 Hz), 125.3 (t, *J* = 6.3 Hz), 128.4, 128.57, 128.61, 135.0, 137.1 (t, *J* = 28.5 Hz), 163.8. ^{19}F NMR (282.65 MHz, $CDCl_3$): δ

–56.04 (d, J = 9.0 Hz). IR (neat) ν 3064, 3034, 2959, 1732, 1456, 1378, 1309, 1280, 1255, 1227, 1176, 1104, 1004, 964, 697 cm^{-1}. HRMS (FAB+) *m/z*: $[M]^+$ calcd for $C_{11}H_9ClF_2O_2$, 246.0259; found 246.0277.

General procedure for the formation of the epoxy esters (GP-1): Benzyl (*E*)-2,3-epoxy-4,4,4-trifluorobutanoate (2b). GP-1A (by use of aqueous NaClO): To a solution of the compound **1b** [2] (0.23 g, 1.00 mmol) in 3.0 mL of CH_3CN was added NaClO aq. (5% in H_2O, 1.50 g, 1.00 mmol) and the whole solution was stirred for 4.5 h at room temperature. This mixture was extracted with CH_2Cl_2 and the usual workup and purification afforded 0.15 g (0.60 mmol) of the pure title compound in 60% yield.

GP-1B (by use of $NaClO·5H_2O$): To a solution of the compound **1b** [2] (0.2302 g, 1.00 mmol) in 3.0 mL of CH_3CN was added $NaClO·5H_2O$ (0.3290 g, 2.00 mmol) at 0 °C, and the whole solution was stirred for 6 h at the same temperature. After the same workup process and purification with silica gel column chromatography using AcOEt:Hex = 1:20 as an eluent, 0.2117 g (0.86 mmol) of the title compound was isolated in 86% yield. Rf = 0.52 (Hex:AcOEt = 5:1). ^{1}H NMR (300.40 MHz, $CDCl_3$): δ 3.71–3.76 (2H, m), 5.21 (1H, d, J = 12.3 Hz), 5.28 (1H, d, J = 12.3 Hz), 7.34–7.44 (5H, m). ^{13}C NMR (75.45 MHz, $CDCl_3$): δ 49.4 (q, J = 2.5 Hz), 52.7 (q, J = 42.2 Hz), 68.0, 121.4 (q, J = 276.0 Hz), 128.5, 128.7, 128.8, 134.3, 165.6. ^{19}F NMR (282.65 MHz, $CDCl_3$): δ
–75.12 (d, J = 4.5 Hz). IR (neat) ν 3944, 3689, 3054, 2987, 2685, 2306, 1756, 1456, 1422, 1382, 1341, 1265, 1169, 1089, 988, 929, 896, 664 cm^{-1}. Anal. calcd for $C_{11}H_9F_3O_3$: C, 53.67; H, 3.68. found C, 53.54; H, 3.89.

Benzyl (*E*)-2,3-epoxy-4,4-difluorobutanoate (2c). 0.2124 g (1.00 mmol) of the compound **1c** [3] was employed instead of **1b** following to **GP-1B**, and the same procedure afforded 0.1803 g of the title compound **2c** (0.79 mmol) in 79% yield. 73% yield by **GP-1A**. Rf = 0.14 (Hex:AcOEt = 20:1). ^{1}H NMR (300.40 MHz, $CDCl_3$): δ 3.56 (1H, ddt, J = 1.8, 3.6, 6.9 Hz), 3.63 (1H, d, J = 1.8 Hz), 5.20 (1H, d, J = 12.0 Hz), 5.27 (1H, d, J = 12.0 Hz), 5.70 (1H, dt, J = 3.3, 54.7 Hz), 7.36-7.39(5H, m). ^{13}C NMR (75.45 MHz, $CDCl_3$): δ 49.1 (t, J = 3.7 Hz), 54.3 (t, J = 32.9 Hz), 67.7, 112.2 (t, J = 241.9 Hz), 128.4, 128.58, 128.65, 134.5, 166.5. ^{19}F NMR (282.65 MHz, $CDCl_3$): δ –126.85 (1F, ddd, J = 6.8, 54.8, 303.3 Hz), –125.00 (1F, ddd, J = 6.8, 54.8, 303.6 Hz). IR (neat) ν 3067, 3036, 2964, 1753, 1456, 1389, 1321, 1295, 1243, 1200, 1146, 1107, 1065, 1001, 910, 753, 698 cm^{-1}. Anal. calcd for $C_{11}H_{10}F_2O_3$: C, 57.90; H, 4.42. found C, 57.95; H, 4.60.

Benzyl (*E*)-4-chloro-2,3-epoxy-4,4-difluorobutanoate (2d). 0.2469 g (1.00 mmol) of the compound **1d** was employed instead of **1b** following to **GP-1B**, and the same procedure afforded 0.2065 g of the title compound **2d** (0.78 mmol) in 78% yield. 45% yield by **GP-1A**. Rf = 0.31 (Hex:AcOEt = 20:1). ^{1}H NMR (300.40 MHz, $CDCl_3$): δ 3.77 (1H, d, J = 0.9 Hz), 3.86 (1H, dt, J

= 1.2, 6.6 Hz), 5.21 (1H, d, *J* = 12.0 Hz), 5.28 (1H, d, *J* = 12.3 Hz), 7.34–7.44 (5H, m). ^{13}C NMR (75.45 MHz, $CDCl_3$): δ 40.6 (dd, *J* = 1.9, 3.1 Hz), 57.5 (dd, *J* = 33.5, 35.9 Hz), 68.0, 123.8 (t, *J* = 291.5 Hz), 128.5, 128.7, 128.8, 134.3, 165.6. ^{19}F NMR (282.65 MHz, $CDCl_3$): δ –65.22 (1F, dd, *J* = 6.8, 168.7 Hz), –63.14 (1F, dd, *J* = 6.8, 168.7 Hz). IR (neat) ν 3490, 3068, 3036, 2960, 1956, 1756, 1456, 1380, 1330, 1291, 1197, 1127, 1042, 983, 924, 752, 698, 608 cm^{-1}. Anal. calcd for $C_{11}H_9ClF_2O_3$: C, 50.30; H, 3.45. found C, 50.46; H, 3.54.

Benzyl (*E*)-2,3-epoxy-4,4,5,5,5-pentafluoropentanoate (2e). 0.2802 g (1.00 mmol) of the compound **1e** [2] was employed instead of **1b** following to **GP-1B**, and the same procedure afforded 0.2148 g (0.73 mmol) of the title compound **2e** in 73% yield. 45% yield by **GP-1A**. Rf = 0.36 (Hex:AcOEt = 20:1). ^{1}H NMR (300.40 MHz, $CDCl_3$): δ 3.70–3.76 (2H, m), 5.22 (1H, d, *J* = 12.3 Hz), 5.28 (1H, d, *J* = 11.7 Hz), 7.36–7.44 (5H, m). ^{13}C NMR (75.45 MHz, $CDCl_3$): δ 48.5(dd, *J* = 2.5, 4.9 Hz), 61.7 (dd, *J* = 26.6, 32.9 Hz), 68.2, 110.2 (qdd, *J* = 38.5, 254.3, 257.4 Hz), 118.2 (ddq, *J* = 34.7, 36.0, 286.0 Hz), 128.5, 128.7, 128.8, 134.3, 165.7. ^{19}F NMR (282.65 MHz, $CDCl_3$): δ –128.92 (1F, dd, *J* = 11.3, 275.9 Hz), –127.04 (1F, dd, *J* = 6.8, 275.9 Hz), −85.15 (3F, s). IR (neat) ν 3069, 3037, 2962, 1758, 1457, 1357, 1327, 1287, 1200, 1149, 1044, 974, 923, 833, 745, 698 cm^{-1}. Anal. calcd for $C_{12}H_9F_5O_3$: C, 48.66; H, 3.06. found C, 48.98; H, 3.08.

Ethyl (*E*)-2,3-epoxy-4,4,5,5,6,6,7,7,8,8,9,9,9-tridecafluorononanonate (2f). 0.4183 g (1.00 mmol) of the compound **1f** [4] was employed instead of **1b** following to **GP-1B**, and the same procedure afforded 0.2630 g (0.61 mmol) of the title compound **2e** in 61% yield. 58% yield by **GP-1A**. Rf = 0.40 (Hex:AcOEt = 20:1). ^{1}H NMR (300.40 MHz, $CDCl_3$): δ 1.34 (3H, t, *J* = 7.2 Hz), 3.71 (1H, d, *J* = 1.5 Hz), 3.77 (1H, brt, *J* = 9.3 Hz), 4.27–4.34 (2H, m). ^{13}C NMR (75.45 MHz, $CDCl_3$): δ 13.8, 48.7 (t, *J* = 3.8 Hz), 52.0 (t, *J* = 28.5 Hz), 62.6, 106.6–116.6 (m), 117.2 (mtq, *J* = 33.0, 286.1 Hz), 165.8. ^{19}F NMR (282.65 MHz, $CDCl_3$): δ –127.31 (2F, m), –124.80 (2F, m), –124.44 (2F, m),
–123.95 (2F, m), –123.17 (2F, m), –81.95 (3F, m). IR (neat) ν 2990, 2945, 1759, 1451, 1339, 1242, 1147, 1026, 927, 850, 744, 721, 708, 653 cm^{-1}. Anal. calcd for $C_{11}H_7F_{13}O_3$: C, 30.43; H, 1.63. found C, 30.27; H, 1.58.

General procedure for the ring opening of epoxides (GP-2). Benzyl 2,3-*anti*-4,4,4-trifluoro-3-hydroxy-2-(*p*-methoxyphenyl)aminobutanoate (3b). *p*-Anisidine (0.07 g, 0.60 mmol) was added to an EtOH (3 mL) solution of the compound **2b** (0.12 g, 0.50 mmol), and the resultant mixture was stirred at 50 °C for 19 h under the open air. After quenching the reaction with 1 M HCl aq., the mixture was extracted with AcOEt three times and the combined organic phase was washed with brine. Evaporation of the volatiles furnished crude materials which were recrystallized by use of Hex:$CHCl_3$ = 3:2 as a solvent to afford 0.14 g (0.39 mmol) of the title compound **3ba** in 78% yield as a sole stereoisomer. Rf = 0.30 (Hex:AcOEt = 2:1). mp 95–97

°C. ^{1}H NMR (300.40 MHz, $CDCl_3$): δ 3.70 (1H, brs), 3.76 (3H, s), 4.31–4.33 (2H, m), 4.39 (1H, brs), 5.14 (1H, dd, *J* = 12.0, 21.3 Hz), 6.74–6.81 (4H, m), 7.26–7.36 (5H, m). ^{13}C NMR (75.45 MHz, acetone-d_6): δ 55.5, 59.3, 67.9, 70.0 (q, *J* = 30.2 Hz), 114.8, 117.7, 124.1 (q, *J* = 283.5 Hz), 128.5, 128.6, 128.7, 134.4, 139.5, 154.3, 170.2. ^{19}F NMR (282.65 MHz, $CDCl_3$): δ –76.83 (d, *J* = 9.0 Hz). IR (KBr) ν 3454, 3315, 2955, 2924, 2854, 2360, 1741, 1519, 1458, 1238, 1204, 1156, 1138, 1097, 1030, 822, 749 cm^{-1}. HRMS (FAB) *m/z*: $[M]^+$ calcd for $C_{18}H_{18}F_3NO_4$, 369.1182; found 369.1209.

Benzyl 2,3-*anti*-4,4-difluoro-3-hydroxy-2-(*p*-methoxyphenyl)aminobutanoate (3ca). Yield 59% as a sole stereoisomer, Rf = 0.30 (Hex:AcOEt = 2:1). mp 68–70 °C. ^{1}H NMR (300.40 MHz, $CDCl_3$): δ 3.75 (3H, s), 4.15 (1H, m), 4.29 (1H, d, *J* = 4.5 Hz), 5.18 (2H, s), 5.89 (1H, dt, *J* = 4.2, 55.6 Hz), 6.68–6.79 (4H, m), 7.27–7.36 (5H, m). ^{13}C NMR (75.45 MHz, acetone-d_6): δ 55.6, 58.8, 67.6, 71.5 (t, *J* = 24.2 Hz), 114.8, 114.7 (t, *J* = 243.5 Hz), 116.5, 128.3, 128.5, 128.6, 134.7, 139.7, 153.6, 170.7. ^{19}F NMR (282.65 MHz, $CDCl_3$): δ –130.84 (1F, ddd, *J* = 14.8, 54.8, 289.7 Hz), –130.65 (1F, ddd, *J* = 4.5, 54.7, 289.6 Hz). IR (neat) ν 3383, 2956, 2836, 1742, 1604, 1514, 1456, 1380, 1290, 1242, 1181, 1151, 1063, 825 cm^{-1}. HRMS (FAB) *m/z*: $[M]^+$ calcd for $C_{18}H_{19}F_2NO_4$, 351.1277; found 351.1266.

Benzyl 2,3-*anti*-4-chloro-4,4-difluoro-3-hydroxy-2-(*p*-methoxyphenyl)amino-butanoate (3da). Yield 76% as a sole stereoisomer, Rf = 0.30 (Hex:AcOEt = 2:1). mp 73–75 °C. ^{1}H NMR (300.40 MHz, $CDCl_3$): δ 3.76 (3H, s), 3.90 (1H, brd, *J* = 11.7 Hz), 4.16 (1H, brs), 4.39 (2H, s), 5.10 (1H, d, *J* = 12.0 Hz), 5.18 (1H, d, *J* = 12.0 Hz), 6.73–6.80 (4H, m), 7.25–7.37 (5H, m). ^{13}C NMR (75.45 MHz, acetone-d_6): δ 55.6, 58.9, 67.9, 74.9 (t, *J* = 26.6 Hz), 114.8, 117.4, 128.2 (t, *J* = 297.4 Hz), 128.5, 128.60, 128.64, 134.4, 139.3, 154.2, 170.7. ^{19}F NMR (282.65 MHz, $CDCl_3$): δ –63.03 (1F, dd, *J* = 9.3, 166.5 Hz), –61.61 (1F, dd, *J* = 7.1, 166.5 Hz). IR (KBr) ν 3300, 2830, 1738, 1596, 1513, 1455, 1385, 1354, 1282, 1249, 1214, 1183, 1105, 1032, 953, 928, 853, 825, 789, 730 cm^{-1}. Anal. calcd for $C_{18}H_{18}ClF_2NO_4$: C, 56.04; H, 4.70; N, 3.63. found C, 55.76; H, 4.67; N, 3.51.

Benzyl 2,3-*anti*-2-(benzylamino)-4,4,4-trifluoro-3-hydroxybutanoate (3bb). Following to **GP-2**, 0.2461g (1.0 mmol) of **2a** in DMF (5 mL) was reacted with benzylamine (0.16 mL, 1.2 mmol) at 40 °C for 7 h under open air. 0.304 g (0.86 mmol) of the title compound **3ab** was obtained after silica gel column chromatography in 86% yield as a sole stereoisomer. Rf = 0.69 (Hex:AcOEt = 1:1). mp 69.6 °C. ^{1}H NMR (300.40 MHz, $CDCl_3$): δ 3.61(1H, d, *J* = 12.4 Hz), 3.64 (1H, d, *J* = 4.8 Hz), 3.96 (1H, d, *J* = 12.6 Hz), 4.14–4.26 (1H, brs), 4.23 (1H, dq, *J* = 7.2, 7.5 Hz), 5.13 (1H, d, *J* = 11.7 Hz), 5.23 (1H, d, *J* = 12.0 Hz), 7.33 (10H, m). ^{13}C NMR (75.45 MHz, $CDCl_3$): δ 52.6, 60.0, 67.6, 69.1 (q, *J* = 33.2 Hz), 128.2, 124.3 (q, *J* = 284.1 Hz), 128.0, 128.39, 128.45, 128.53, 128.6, 134.7, 138.2, 170.4. ^{19}F NMR (282.65 MHz, $CDCl_3$): δ –76.46 (d, *J* = 6.7 Hz). IR (KBr) ν 3526, 3042, 2986, 2955, 2924, 2860, 2737, 2431, 1772, 1669, 1447, 1348, 1284,

1268, 1243, 1190, 1159, 1091, 1037, 963, 923, 888, 855, 821, 782 cm^{-1}. Anal. calcd for $C_{18}H_{18}F_3NO_3$: C, 61.19; H, 5.13; N, 3.96. found C, 61.55; H, 5.29; N, 4.29.

Benzyl 2,3-*anti*-2-*n*-butylamino-4,4,4-trifluoro-3-hydroxybutanoate (3bc). Yield 48% as a sole stereoisomer, Rf = 0.40 (Hex:AcOEt = 2:1). mp 54.8 °C. ^{1}H NMR (300.40 MHz, $CDCl_3$): δ 0.90 (3H, t, *J* = 7.2 Hz), 1.28–1.51 (4H, m), 2.60 (1H, td, *J* = 6.9, 11.4 Hz), 2.72 (1H, td, *J* = 7.2, 11.4 Hz), 3.50 (1H, d, *J* = 4.2 Hz), 4.08 (1H, dq, *J* = 4.2, 6.9 Hz), 5.19 (1H, d, *J* = 12.3 Hz), 5.24 (1H, d, *J* = 12.0 Hz), 7.34–7.40 (5H, m). ^{13}C NMR (75.45 MHz, $CDCl_3$): δ 13.7, 20.1, 31.9, 48.9, 61.1, 67.6, 68.8 (q, *J* = 30.2 Hz), 124.3 (q, *J* = 281.8 Hz), 128.5, 128.6, 134.8, 170.6. ^{19}F NMR (282.65 MHz, $CDCl_3$): δ –76.53 (d, *J* = 6.8 Hz). IR (KBr) ν 3279, 3091, 2960, 2367, 2358, 2342, 1740, 1450, 1380, 1294, 1265, 1227, 1213, 1174, 1138, 1096, 916, 868, 755, 482 cm^{-1}. Anal. calcd for $C_{15}H_{20}F_3NO_3$: C, 56.42; H, 6.21; N, 4.39. found C, 56.33; H, 5.90; N, 4.54.

Benzyl 2,3-*anti*-2-(1-phenylethylamino)-4,4,4-trifluoro-3-hydroxybutanoate (3bd). Yield 77%, Dr = 53:47, Rf = 0.27 (Hex:AcOEt = 4:1). ^{1}H NMR (300.40 MHz, $CDCl_3$): δ 1.36 (3H, d, *J* = 6.3 Hz) and 1.41 (3H, d, *J* = 6.3 Hz), 3.41 (1H, d, *J* = 5.1 Hz), 3.70 (1H, *J* = 4.8 Hz), 3.74–3.81 (2H, m), 4.09 (1H, dq, *J* = 5.1, 7.2 Hz) and 4.23 (1H, dq, *J* = 5.1, 7.2 Hz), 4.76 (2H, brs), 5.01 (1H, d, *J* = 12.0 Hz), 5.08 (1H, d, *J* = 12.6 Hz), 5.12 (1H, d, *J* = 12.6 Hz), 5.24 (1H, d, *J* = 12.0 Hz), 7.21–7.39 (20H, m). ^{13}C NMR (75.45 MHz, $CDCl_3$): δ 22.0 and 24.6, 56.9 and 57.1, 58.3 and 58.5, 67.51 and 67.53, 68.9 (q, *J* = 29.8 Hz) and 69.1 (q, *J* = 30.0 Hz), 124.3 (q, *J* = 284.0 Hz) and 124.4 (q, *J* = 284.0 Hz), 126.7 and 127.0, 127.6 and 127.8, 128.43 and 128.49, 128.57 and 128.59, 128.8, 134.7 and 134.8, 170.5 and 170.7. ^{19}F NMR (282.65 MHz, $CDCl_3$): δ –76.58 (d, *J* = 4.5 Hz; major), –76.35 (d, *J* = 6.8 Hz; minor). IR (neat) ν 3275, 3087, 2875, 2360, 2342, 1736, 1456, 1385, 1289, 1268, 1189, 1128, 1096, 915, 865, 763, 699, 530, 493, 436 cm^{-1}. HRMS (FAB) *m/z*: $[M+H]^+$ calcd for $C_{19}H_{21}F_3NO_3$, 368.1468; found 368.1480.

Benzyl (2*R,3*S**)-2,3-*anti*-2-((2'*R**)-1-phenylethylamino)-4,4,4-trifluoro-3-hydroxy-butanoate ((2*R**,3*S**,2'*R**)-3bd).** Recrystallization of the above mixture in AcOEt under the atmosphere of hexane afforded a good crystal appropriate for the X-ray crystallographic analysis whose analytical data are as follows. mp 79.3–79.9 °C. ^{1}H NMR (300.40 MHz, $CDCl_3$): δ 1.41 (3H, d, *J* = 6.3 Hz), 1.63 (1H, br s), 3.42 (1H, d, *J* = 4.8 Hz), 3.79 (1H, q, *J* = 6.3 Hz), 4.10 (1H, m), 5.13 (1H, d, *J* = 12.3 Hz), 5.44 (1H, d, *J* = 12.0 Hz), 7.21–7.38 (10H, m). ^{13}C NMR (75.45 MHz, $CDCl_3$): δ 24.7, 56.9, 58.3, 67.6, 68.8 (q, *J* = 30.2 Hz), 124.3 (q, *J* = 284.0 Hz), 127.1, 128.5, 128.6, 128.8, 134.7, 142.9, 170.6. ^{19}F NMR (282.65 MHz, $CDCl_3$): δ –76.37 (d, *J* = 6.8 Hz). IR (KBr) ν 3274, 3090, 2873, 2267, 1735, 1457, 1293, 1123, 760, 529 cm^{-1}. HRMS (FAB) *m/z*: $[M+H]^+$ calcd for $C_{19}H_{21}F_3NO_3$, 368.1468; found 368.1484.

Benzyl 2,3-*anti*-2-[{(*S*)-1-(benzyloxycarbonyl)-2-methylbutyl}amino]-4,4,4-trifluoro-3-hydroxybutanoate (3be). Yield 72%, Dr = 53:47, Rf = 0.57 (Hex:AcOEt = 2:1). ^{1}H NMR

(300.40 MHz, $CDCl_3$): δ 0.84 (6H, t, *J* = 7.2 Hz), 0.88 (3H, d, *J* = 6.9 Hz) and 0.95 (3H, d, *J* = 6.9 Hz), 1.14–1.28 (2H, m), 1.34–1.54 (2H, m), 1.69–1.93 (2H, m), 3.08 (1H, d, *J* = 6.0 Hz) and 3.31 (1H, d, *J* = 4.5 Hz), 3.53 (1H, d, *J* = 4.5 Hz) and 3.58 (1H, d, *J* = 5.1 Hz), 4.24 (1H, m), 4.78 (2H, brs), 5.01 (1H, d, *J* = 12.3 Hz), 5.08 (1H, d, *J* = 11.7 Hz), 5.09 (2H, s), 5.10 (1H, d, *J* = 18.6 Hz), 5.16 (2H, s), 5.17 (1H, d, *J* = 18.6 Hz), 7.31–7.38 (10H, m). ^{13}C NMR (75.45 MHz, $CDCl_3$): δ 21.7 and 21.9, 22.6 and 22.8, 24.4 and 24.7, 41.9 and 42.2, 58.7, 60.0, 60.2, 60.5, 66.7 and 67.0, 67.5 and 67.8, 69.3 (q, *J* = 29.6 Hz) and 70.0 (q, *J* = 25.9 Hz), 124.2 (q, *J* = 279.9 Hz) and 124.4 (q, *J* = 282.3 Hz), 128.2, 128.27, 128.31, 128.41, 128.43, 128.47, 128.50, 128.53, 128.56, 128.61, 134.50 and 134.53, 134.5 (q, *J* = 1.8 Hz) and 134.7 (q, *J* = 1.9 Hz), 135.1 and 135.3, 170.1, 170.4 (q, *J* = 4.3 Hz), 170.4 (q, *J* = 3.7 Hz), 174.4. ^{19}F NMR (282.65 MHz, $CDCl_3$): δ –75.89 (d, *J* = 6.8 Hz) and –76.25(d, *J* = 6.7 Hz). IR (neat) ν 3341, 3035, 2958, 2871, 2388, 2347, 1738, 1498, 1455, 1170, 966, 910, 734, 697, 466, 457, 449, 435, 428, 419 cm^{-1}. Anal. calcd for $C_{24}H_{28}F_3NO_5$: C, 61.66; H, 6.04; N, 3.00. found C, 61.44; H, 6.15; N, 3.09.

Benzyl 2,3-*anti*-2-(*N,N*-diethylamino)-4,4,4-trifluoro-3-hydroxybutanoate (3bf). Yield 83%, Rf = 0.69 (Hex:AcOEt = 2:1). ^{1}H NMR (300.40 MHz, $CDCl_3$): δ 1.02 (3H, t, *J* = 6.9 Hz), 1.07 (3H, t, *J* = 6.9 Hz), 2.49 (1H, q, *J* = 6.9 Hz), 2.53 (1H, q, *J* = 6.6 Hz), 2.47–2.58 (2H, m), 2.65–2.79 (2H, m), 3.57 (1H, d, *J* = 9.3 Hz), 3.74 (1H, d, *J* = 6.9 Hz), 4.33 (1H, dq, *J* = 6.9, 7.2 Hz), 5.15–5.26 (2H, m), 7.37 (5H, brs). ^{13}C NMR (75.45 MHz, $CDCl_3$): δ 13.2, 44.9, 62.4, 66.8, 68.9 (q, *J* = 28.4 Hz), 124.5 (q, *J* = 279.9 Hz), 128.3, 128.4, 128.5, 135.2, 170.9. ^{19}F NMR (282.65 MHz, $CDCl_3$): δ –76.79 (d, *J* = 7.5 Hz). IR (neat) ν 3453, 3035, 2976, 2877, 1736, 1455, 1275, 1169, 1132, 996, 864, 752, 698, 487, 467, 449, 435, 428, 418 cm^{-1}. Anal. calcd for $C_{15}H_{20}F_3NO_3$: C, 56.42; H, 6.31; N, 4.39. found C, 56.50; H, 6.06; N, 4.34.

Benzyl 2,3-*anti*-2-(pyrrolidin-1-yl)-4,4,4-trifluoro-3-hydroxybutanoate (3bg). Yield 56%, Rf = 0.42 (Hex:AcOEt = 2:1). mp 102.1 °C. ^{1}H NMR (300.40 MHz, $CDCl_3$): δ 1.74–1.78 (4H, m), 2.67–2.72 (4H, m), 3.59 (1H, d, *J* = 4.2 Hz), 4.15 (1H, d, *J* = 6.6 Hz), 4.32 (1H, m), 5.16 (1H, d, *J* = 12.3 Hz), 5.26 (1H, d, *J* = 12.0 Hz), 7.37 (5H, brs). ^{13}C NMR (75.45 MHz, $CDCl_3$): δ 23.4, 50.5, 63.9, 67.0, 70.1 (q, *J* = 29.6 Hz), 124.3 (q, *J* = 280.5 Hz), 128.45, 128.48, 128.5, 135.0, 169.9. ^{19}F NMR (282.65 MHz, $CDCl_3$): δ –78.06 (d, *J* = 6.7 Hz). IR (KBr) ν 2984, 2858, 2367, 2358, 2342, 1739, 1462, 1402, 1306, 1268, 1220, 1169, 1120, 911, 898, 861, 743, 701, 584, 501 cm^{-1}. Anal. calcd for $C_{15}H_{18}F_3NO_3$: C, 56.78; H, 5.72; N, 4.52. found C, 56.71; H, 5.53; N, 4.52.

Benzyl 2,3-*anti*-4,4,4-trifluoro-3-hydroxy-2-(phenylmethylsulfenyl)butanoate (4ba). 0.0739 g (0.30 mmol) of **2b** in DMF (1.0 mL) was reacted with benzyl mercaptan (0.040 mL, 0.36 mmol) at room temperature for 5 h under an argon atmosphere. 0.0996 g (0.27 mmol) of the title compound **4ba** was obtained after silica gel column chromatography in 90% yield as an inseparable 94:6 diastereomer mixture. Rf = 0.37 (Hex:AcOEt = 4:1). IR (neat) ν 3427, 3032,

2952, 1704, 1496, 1455, 1374, 1272, 1167, 1130, 1081, 960, 744, 703, 663, 560 cm^{-1}. HRMS (FAB+) *m/z*: $[M+2H]^+$ calcd for $C_{18}H_{19}F_3O_3S$, 372.1002; found 372.1030. **major isomer:** ^1H NMR (300.40 MHz, $CDCl_3$): δ 3.47 (1H, d, *J* = 3.3 Hz), 3.80 (1H, d, *J* = 13.2 Hz), 3.88 (1H, d, *J* = 13.2 Hz), 4.09–4.20 (1H, m), 4.51 (1H, d, *J* = 10.2 Hz), 5.15 (1H, d, *J* = 12.0 Hz), 5.24 (1H, d, *J* = 12.0 Hz), 7.22–7.39 (10H, m). ^{13}C NMR (75.45 MHz, $CDCl_3$): δ 36.4, 43.1, 67.7, 72.4 (q, *J* = 31.6 Hz), 123.7 (q, *J* = 284.1 Hz), 127.6, 128.4, 128.61, 128.64, 128.7, 128.9, 134.6, 135.9, 171.1. ^{19}F NMR (282.65 MHz, $CDCl_3$): δ –78.60 (d, *J* = 6.8 Hz). **minor isomer:** ^{13}C NMR δ 35.7, 46.9, 67.6, 68.6 (q, *J* = 31.0 Hz), 127.7, 128.3, 128.5, 128.6, 129.0, 129.3, 134.8, 136.1, 169.3. ^{19}F NMR (282.65 MHz, $CDCl_3$): δ –78.32 (d, *J* = 7.3 Hz).

Benzyl 2,3-*anti*-4,4-difluoro-3-hydroxy-2-(phenylmethylsulfenyl)butanoate (4ca). 0.1369 g (0.60 mmol) of **2c** was used for 48 h instead of **2b** and 0.1606 g (0.46 mmol) of the title compound **4ca** was obtained after silica gel column chromatography in 76% yield as a single diastereomer. Rf = 0.26 (Hex:AcOEt = 2:1). ^1H NMR (300.40 MHz, $CDCl_3$): δ 3.32 (1H, d, *J* = 8.1 Hz), 3.42 (1H, d, *J* = 6.6 Hz), 3.79 (1H, d, *J* = 13.2 Hz), 3.84 (1H, d, *J* = 13.2 Hz), 3.97–4.11 (1H, m), 5.16 (1H, d, *J* = 12.3 Hz), 5.25 (1H, d, *J* = 12.3 Hz), 5.91 (1H, dt, *J* = 2.4, 55.0 Hz), 7.21–7.43 (10H, m). ^{13}C NMR (75.45 MHz, $CDCl_3$): δ 36.5, 44.6 (dd, *J* = 2.5, 4.4 Hz), 67.4, 71.2 (t, *J* = 22.9 Hz), 114.1 (dd, *J* = 243.8, 245.3 Hz), 127.5, 128.2, 128.5, 128.6, 129.0, 134.9, 136.3, 170.8. ^{19}F NMR (282.65 MHz, $CDCl_3$): δ –133.36 (ddd, *J* = 11.3, 54.6, 287.5 Hz), −131.59 (ddd, *J* = 13.6, 26.6, 287.5 Hz). IR (neat) ν 3465, 1728, 1496, 1455,1380, 1337, 1276, 1151, 1058, 751, 699 cm^{-1}. HRMS (FAB+) *m/z*: $[M+H]^+$ calcd for $C_{18}H_{19}F_2O_3S$, 353.1017; found 353.1047.

Benzyl 2,3-*anti*-4-chloro-4,4-difluoro-3-hydroxy-2-(phenylmethylsulfenyl)-butanoate (4da). 0.1576 g (0.60 mmol) of **2d** was used for 24 h instead of **2b** and 0.2020 g (0.52 mmol) of the title compound **4da** was obtained after silica gel column chromatography in 87% yield as an inseparable 90:10 diastereomer mixture. Rf = 0.34 (Hex:AcOEt = 4:1). IR (neat) ν 3427, 3021, 2951, 1731, 1707, 1496, 1455, 1379, 1278, 1106, 959, 746, 697 cm^{-1}. HRMS (FAB+) *m/z*: $[M+H]^+$ calcd for $C_{18}H_{18}ClF_2O_3S$, 387.0628; found 387.0647. **major isomer:** ^1H NMR (300.40 MHz, $CDCl_3$): δ 3.59 (1H, d, *J* = 2.7 Hz), 3.81 (1H, d, *J* = 13.2 Hz), 3.89 (1H, d, *J* = 13.2 Hz), 4.13–4.23 (1H, m), 4.85 (1H, d, *J* = 10.2 Hz), 5.15 (1H, d, *J* = 12.0 Hz), 5.24 (1H, d, *J* = 12.0 Hz), 7.22–7.40 (10H, m). ^{13}C NMR (75.45 MHz, $CDCl_3$): δ 36.5, 43.0, 67.7, 77.6 (t, *J* = 27.3 Hz), 127.6, 128.0 (t, *J* = 298.3 Hz), 128.5, 128.63, 128.65, 128.7, 129.0, 134.5, 135.9, 171.4. ^{19}F NMR (282.65 MHz, $CDCl_3$): δ –65.54 (dd, *J* = 9.0, 166.5 Hz), –64.09 (dd, *J* = 9.0, 166.5 Hz). **minor isomer:** ^1H NMR (300.40 MHz, $CDCl_3$): δ 3.14 (1H, d, *J* = 5.1 Hz), 3.79 (1H, d, *J* = 12.9 Hz), 3.87 (1H, d, *J* = 12.6 Hz), 4.38–4.63 (1H, m). ^{13}C NMR (75.45 MHz, $CDCl_3$): δ 35.8, 47.7, 67.6, 77.6 (t, *J* = 27.3 Hz), 127.7, 128.3, 128.5, 128.6, 129.1, 134.9, 136.0. ^{19}F NMR (282.65 MHz, $CDCl_3$): δ –65.40 (dd, *J* = 7.1, 168.7 Hz), –61.81 (dd, *J* = 7.1, 168.7 Hz).

Benzyl 2,3-*anti*-4,4,5,5,5-pentafluoro-3-hydroxy-2-(phenylmethylsulfenyl)-butanoate (4ea). 0.1777 g (0.60 mmol) of **2e** was used for 72 h instead of **2b** and 0.2051 g (0.49 mmol) of the title compound **4ea** was obtained after silica gel column chromatography in 72% yield as an inseparable 69:31 diastereomer mixture. Rf = 0.49 (Hex:AcOEt = 4:1). IR (neat) ν 3449, 3031, 1717, 1455, 1383, 1333, 1277, 1217, 1191, 1119, 1052, 749, 699 cm^{-1}. HRMS (FAB+) *m/z*: $[M+H]^+$ calcd for $C_{19}H_{18}F_5O_3S$, 421.0891; found 421.0924. **major isomer:** ^{1}H NMR (300.40 MHz, $CDCl_3$): δ 3.50 (1H, t, *J* = 2.1 Hz), 3.82 (1H, d, *J* = 13.2 Hz), 3.91 (1H, d, *J* = 13.2 Hz), 4.23 (1H, dddd, *J* = 2.1, 3.6, 11.1, 22.2 Hz), 4.82 (1H, d, *J* = 10.8 Hz), 5.16 (1H, d, *J* = 12.0 Hz), 5.25 (1H, d, *J* = 12.0 Hz), 7.18–7.45 (10H, m). ^{13}C NMR (75.45 MHz, $CDCl_3$): δ 36.7, 41.7, 67.9, 72.5 (dd, *J* = 21.1, 28.5 Hz), 109.4–124.7 (m), 127.7, 128.4, 128.5, 128.7, 128.8, 129.1, 134.5, 136.0, 171.9. ^{19}F NMR (282.65 MHz, $CDCl_3$): δ –132.28 (1F, dd, *J* = 22.9, 273.6 Hz), −121.51 (1F, d, *J* = 271.3 Hz), –83.27 (3F, s). **minor isomer:** ^{1}H NMR (300.40 MHz, $CDCl_3$): δ 3.09 (1H, dd, *J* = 2.1, 4.5 Hz), 3.63 (1H, d, *J* = 8.4 Hz), 4.41–4.52 (1H, m), 5.15 (1H, d, *J* = 12.3 Hz), 5.22 (1H, d, *J* = 12.0 Hz). ^{13}C NMR (75.45 MHz, $CDCl_3$): δ 36.7, 46.5, 66.9 (dd, *J* = 21.1, 29.7 Hz), 67.7, 127.9, 128.6, 128.7, 128.82, 128.84, 129.1, 134.9, 136.0, 169.2. ^{19}F NMR (282.65 MHz, $CDCl_3$): δ –133.91 (1F, dd, *J* = 20.4, 276.1 Hz), –122.25 (1F, d, *J* = 276.1 Hz), −83.62 (3F, s).

Benzyl 2,3-*anti*-2-(decylsulfenyl)-4,4,4-trifluoro-3-hydroxybutanoate (4bb). 0.1477 g (0.60 mmol) of **2b** was reacted with 1-decanethiol (0.15 mL, 0.72 mmol) and 0.1485 g (0.35 mmol) of the title compound **4bb** was obtained after silica gel column chromatography in 59% yield as a single diastereomer. Rf = 0.23 (Hex:AcOEt = 10:1). ^{1}H NMR (300.40 MHz, $CDCl_3$): δ 0.88 (3H, t, *J* = 6.9 Hz), 1.21–1.34 (14H, m), 1.53 (1H, quint, *J* = 7.5 Hz), 2.64 (2H, dt, *J* = 2.4, 7.5 Hz), 3.55 (1H, d, *J* = 3.3 Hz), 4.21–4.33 (1H, m), 4.51 (1H, d, *J* = 9.9 Hz), 5.18 (1H, d, *J* = 12.3 Hz), 5.28 (1H, d, *J* = 12.3 Hz), 7.33–7.42 (5H, m). ^{13}C NMR (75.45 MHz, $CDCl_3$): δ 14.0, 22.6, 28.6, 28.9, 29.0, 29.2, 29.4, 29.5, 31.8, 32.3, 43.7, 67.7, 72.6 (q, *J* = 31.0 Hz), 123.8 (q, *J* = 283.5 Hz), 128.4, 128.60, 128.64, 134.6, 171.4. ^{19}F NMR (282.65 MHz, $CDCl_3$): δ –78.64 (d, *J* = 6.8 Hz). IR (neat) ν 3455, 2926, 2855, 1719, 1457, 1380, 1340, 1270, 1177, 1132, 1083, 749, 697 cm^{-1}. HRMS (FAB+) *m/z*: $[M+H]^+$ calcd for $C_{21}H_{32}F_3O_3S$, 421.2019; found 421.2041.

Benzyl 2,3-*anti*-4,4,4-trifluoro-3-hydroxy-2-(phenylsulfenyl)butanoate (4bc). 0.1477 g (0.60 mmol) of **2b** was reacted with thiophenol (0.073 mL, 0.72 mmol) and 0.1969 g (0.55 mmol) of the title compound **4bc** was obtained after silica gel column chromatography in 92% yield as an inseparable 93:7 diastereomer mixture. Rf = 0.43 (Hex:AcOEt = 4:1). IR (neat) ν 3459, 1719, 1441, 1383, 1343, 1269, 1176, 1132, 1085, 748, 694 cm^{-1}. HRMS (FAB+) *m/z*: $[M]^+$ calcd for $C_{17}H_{15}F_3O_3S$, 356.0689; found 356.0704. **major isomer:** ^{1}H NMR (300.40 MHz, $CDCl_3$): δ 3.93 (1H, d, *J* = 3.3 Hz), 4.25–4.37 (1H, m), 4.50 (1H, d, *J* = 9.9 Hz), 5.10 (1H, d, *J* = 12.0 Hz), 5.15 (1H, d, *J* = 12.3 Hz), 7.22–7.52 (10H, m). ^{13}C NMR (75.45 MHz, $CDCl_3$): δ 48.7

(q, J = 1.2 Hz), 67.8, 71.7 (q, J = 31.6 Hz), 123.8 (q, J = 283.5 Hz), 128.4, 128.5, 128.6, 129.2, 129.3, 131.0, 134.0, 134.3, 170.7. ^{19}F NMR (282.65 MHz, $CDCl_3$): δ –78.38 (d, J = 7.1 Hz). **minor isomer:** ^{1}H NMR (300.40 MHz, $CDCl_3$): δ 3.35 (1H, d, J = 4.8 Hz), 3.63 (1H, d, J = 7.8 Hz). ^{13}C NMR (75.45 MHz, $CDCl_3$): δ 52.2, 67.6, 71.7 (q, J = 31.6 Hz), 128.5, 129.1, 129.2, 129.5, 129.3, 134.2, 134.7, 168.9. ^{19}F NMR (282.65 MHz, $CDCl_3$): δ –77.88 (d, J = 4.5 Hz).

Benzyl 2,3-*anti*-4,4,4-trifluoro-3-hydroxy-2-{(methoxycarbonyl)methyl-sulfenyl)}butanoate (4bd). 0.1477 g (0.60 mmol) of **2b** was reacted with methyl thioglycolate (0.065 mL, 0.72 mmol) and 0.1985 g (0.56 mmol) of the title compound **4ad** was obtained after silica gel column chromatography in 94% yield as an inseparable 95:5 diastereomer mixture. Rf = 0.43 (Hex:AcOEt = 2:1). IR (neat) ν 3457, 1737, 1455, 1439, 1383, 1301, 1271, 1174, 1132, 1005, 751, 698 cm^{-1}. HRMS (FAB+) *m/z*: $[M+H]^+$ calcd for $C_{21}H_{32}F_3O_3S$, 353.0665; found 353.0663. **major isomer:** ^{1}H NMR (300.40 MHz, $CDCl_3$): δ 3.42 (1H, d, J = 15.9 Hz), 3.48 (1H, d, J = 15.9 Hz), 3.72 (3H, s), 3.81 (1H, d, J = 3.0 Hz), 4.32–4.43 (1H, m), 4.54 (1H, d, J = 10.2 Hz), 5.21 (1H, d, J = 12.0 Hz), 5.26 (1H, d, J = 12.3 Hz), 7.33–7.42 (5H, m). ^{13}C NMR (75.45 MHz, $CDCl_3$): δ 33.1, 43.9, 52.6, 67.9, 72.2 (q, J = 31.6 Hz), 123.7 (q, J = 283.4 Hz), 128.3, 128.59, 128.63, 134.5, 169.8, 170.4. ^{19}F NMR (282.65 MHz, $CDCl_3$): δ –78.53 (d, J = 6.8 Hz). **minor isomer:** ^{13}C NMR (75.45 MHz, $CDCl_3$): δ 31.8, 41.0, 53.2, 67.6, 128.2, 128.5, 129.3, 134.7, 172.1. ^{19}F NMR (282.65 MHz, $CDCl_3$): δ –78.20 (d, J = 6.8 Hz).

Benzyl 2,3-*anti*-2-chloro-4,4,4-trifluoro-3-hydroxybutanoate (5ba). A mixture of 0.2462 g (1.00 mmol) of **2b**, $MgCl_2 \cdot 6H_2O$ (0.3050 g, 1.5 mmol), and acetone (5.0 mL) in an ace pressure tube was stirred for 24 h at the reflux temperature under an argon atmosphere. After the addition of a saturated aqueous NH_4Cl solution, the reaction mixture was extracted with AcOEt three times, and dried over anhydrous Na_2SO_4. Evaporation of the volatiles afforded crude materials which was purified by silica gel column chromatography using Hex:AcOEt = 4:1 as an eluent to furnish 0.1894 g (0.67mmol) of the title compound **5ba** in 67% yield as an inseparable 97:3 diastereomer mixture with 19% of recovery of the substrate (determined by ^{19}F NMR). mp 46.5 °C, Rf = 0.34 (Hex:AcOEt = 4:1). ^{1}H NMR (300.40 MHz, $CDCl_3$): d 3.14 (1H, δ, J = 8.7 Hz), 4.41 (1H, quint, J = 6.0 Hz), 4.49 (1H, d, J = 5.4 Hz), 5.24 (1H, d, J = 12.3 Hz), 5.29 (1H, d, J = 12.0 Hz), 7.33–7.42(5H, m). ^{13}C NMR (75.45 MHz, $CDCl_3$): δ 51.7, 68.7, 71.9 (q, J = 31.6 Hz), 123.3 (q, J = 282.9 Hz), 128.4, 128.7, 128.8, 134.0, 167.6. ^{19}F NMR (282.65 MHz, $CDCl_3$): δ –77.03 (d, J = 6.8 Hz; major), –77.24 (d, J = 4.8 Hz; minor). IR (KBr) ν 3494, 3070, 3039, 2994, 2964, 2359, 2342, 1719, 1395, 1329, 1267, 1182, 1132, 907, 754, 742, 697, 676, 667, 658, 498 cm^{-1}. Anal. Calcd for $C_{11}H_{10}F_3ClO_3$: C, 46.74; H, 3.57; Found: C, 46.62; H, 3.32.

Benzyl 2,3-*anti*-2-bromo-4,4,4-trifluoro-3-hydroxybutanoate (5bb). 0.4920 g (2.00 mmol) of **2b** was added at 0 °C to a solution of $MgBr_2$ (0.5613 g, 2.00 mmol) in acetone (10.0 mL) under an argon atmosphere and the resultant mixture was stirred for 3 h at that temperature.

The same workup process and purification furnished 0.3889 g (1.18 mmol) of the title compound **5bb** in 59% yield with 18% recovery of the substrate (determined by ^{19}F NMR). Rf = 0.34 (Hex:AcOEt = 4:1). ^{1}H NMR (300.40 MHz, $CDCl_3$): δ 3.20 (1H, brs), 4.52 (1H, dq, *J* = 4.5, 6.0 Hz), 4.59 (1H, d, *J* = 4.5 Hz), 5.21 (1H, d, *J* = 12.0 Hz), 5.28 (1H, d, *J* = 12.0 Hz), 7.38–7.43 (5H, m). ^{13}C NMR (75.45 MHz, $CDCl_3$): δ 37.5, 68.6, 72.1 (q, *J* = 31.8Hz), 123.2 (q, *J* = 283.4 Hz), 128.4, 128.8, 128.9, 134.0, 168.5. ^{19}F NMR (282.65 MHz, $CDCl_3$): δ –77.30 (d, *J* = 6.8 Hz). IR (KBr) ν 3492, 3070, 3039, 2963, 2361, 2341, 1719, 1455, 1394, 1328, 1267, 1181, 1132, 1086, 961, 907, 754, 697, 505, 406 cm^{-1}. HRMS (FAB+) *m/z*: $[M-H]^+$ calcd for $C_{11}H_9BrF_3O_3$, 324.9682; found 324.9684.

Benzyl 2,3-*anti*-4,4,4-trifluoro-3-hydroxy-2-iodobutanoate (5bc). 0.1231 g (0.50 mmol) of **2b** was added at room temperature to a solution of Mg (0.0134 g, 0.55 mmol), I_2 (0.1396 g, 0.55 mmol) in Et_2O (5.0 mL) under an argon atmosphere and the resultant mixture was stirred for 3 h at that temperature. The same workup process and purification furnished 0.1230 g (0.33 mmol) of the title compound **5bc** in 67% yield with 6% recovery of the substrate (determined by ^{19}F NMR). Rf = 0.31 (Hex:AcOEt = 4:1). ^{1}H NMR (300.40 MHz, $CDCl_3$): δ 4.14–4.25 (1H, m), 4.62 (1H, d, *J* = 3.3 Hz), 4.70 (1H, d, *J* = 5.1 Hz), 5.19 (1H, d, *J* = 12.0 Hz), 5.24 (1H, d, *J* = 12.0 Hz), 7.34–7.40 (5H, m). ^{13}C NMR (75.45 MHz, $CDCl_3$): δ 10.6, 68.4, 74.0 (q, *J* = 31.6 Hz), 122.7 (q, *J* = 285.6 Hz), 128.4, 128.7, 128.8, 134.1, 170.8. ^{19}F NMR (282.65 MHz, $CDCl_3$): δ −77.70 (d, *J* = 7.1 Hz). IR (neat) ν 3456, 1719, 1456, 1385, 1346, 1265, 1187, 1138, 1076, 1005, 747, 697 cm^{-1}. HRMS (FAB+) *m/z*: $[M+H]^+$ calcd for $C_{11}H_{11}F_3IO_3$, 374.9699; found 374.9730.

Benzyl 4,4,4-trifluoro-3-hydroxybutanoate (6b) [5,6]**.** 0.0748 g (0.20 mmol) of **5bc** was added at room temperature to an acetone (2 mL) solution of LiI (0.0540 g, 0.40 mmol) under an argon atmosphere and the resultant mixture was stirred for 24 h at that temperature. The same workup process and purification furnished an inseparable mixture of **6b** and **5bc** and the yield of the former was determined by ^{19}F NMR as 54%. Rf = 0.31 (Hex:AcOEt = 4:1). ^{1}H NMR (300.40 MHz, $CDCl_3$): δ 2.74 (1H, dd, *J* = 8.1, 16.8 Hz), 2.80 (1H, dd, *J* = 4.2, 16.8 Hz), 3.33 (1H, d, *J* = 5.4 Hz), 4.47 (1H, m), 5.20 (2H, s), 7.33–7.42 (5H, m). ^{13}C NMR (75.45 MHz, $CDCl_3$): δ 34.8 (q, *J* = 1.2 Hz), 67.0 (q, *J* = 32.2 Hz), 67.3, 124.4 (q, *J* = 280.9 Hz), 128.3, 128.5, 128.6, 134.9, 170.6. ^{19}F NMR (282.65 MHz, $CDCl_3$): δ –80.99 (d, *J* = 6.8 Hz).

General Procedure for the ring opening of epoxides by enolates (GP-3). 4-Benzyl 5-ethyl *anti*,*syn*-tetrahydro-2-oxo-3-(trifluoromethyl)furan-4,5-dicarboxylate (*anti*,*syn*-7a) and 4,5-diethyl *anti*,*syn*-tetrahydro-2-oxo-3-(trifluoromethyl)furan-4,5-dicarboxylate (*anti*,*syn*-7b). 0.18 mL (1.20 mmol) of diethyl malonate was added to a flask containing 0.0673 g (0.60 mmol) of *t*-BuOK in DMSO (1.8 mL) under an argon atmosphere and the resultant mixture was stirred for 15 min at room temperature. 0.1477 g (0.60 mmol) of **2b** in 0.8 mL of DMSO was introduced to the resultant solution and the stirring was continued for 0.5 h. The same workup

process and purification furnished 0.1717 g of an inseparable mixture of ***anti,syn*-7a** (dr = 99:1) and ***anti,syn*-7b** (**7a**:**7b** = 83:17). ***Anti,syn*-7a:** Rf = 0.34 (Hex:AcOEt = 4:1). ^{1}H NMR (300.40 MHz, $CDCl_3$): δ 1.32 (3H, t, *J* = 7.2 Hz), 4.20–4.27 (2H, m), 4.21–4.35 (2H, m), 5.05 (1H, quint, *J* = 7.2 Hz), 5.15 (1H, d, *J* = 12.3 Hz), 5.23 (1H, d, *J* = 12.0 Hz), 7.32–7.40 (5H, m). ^{13}C NMR (75.45 MHz, $CDCl_3$): δ 13.8, 44.4, 46.4, 63.1, 68.5, 73.5 (q, *J* = 34.1 Hz), 122.5 (q, *J* = 282.9 Hz), 128.61, 128.63, 128.8, 134.0, 165.1, 165.6, 167.4. ^{19}F NMR (282.65 MHz, $CDCl_3$): δ −75.84 (d, *J* = 6.8 Hz). IR (neat) ν 2987, 1813, 1742, 1457, 1389, 1321,1218, 1182, 1128, 1023, 972, 755 cm^{-1}. HRMS (FAB+) *m/z*: $[M+H]^+$ calcd for $C_{16}H_{16}F_3O_6$, 361.0893; found 361.0911. **Epimer at the 2 position of *anti,syn*-7a (*syn,syn*-7a):** ^{1}H NMR (300.40 MHz, $CDCl_3$): δ 1.30 (3H, t, *J* = 7.2 Hz), 4.00 (1H, d, *J* = 8.4 Hz), 4.08 (1H, dd, *J* = 6.3, 8.1 Hz), 4.26–4.34 (2H, m), 5.00 (1H, quint, *J* = 5.7 Hz), 5.22 (1H, d, *J* = 12.0 Hz), 5.27 (1H, d, *J* = 12.3 Hz), 7.31–7.41 (5H, m). ^{13}C NMR (75.45 MHz, $CDCl_3$): δ 13.9, 43.2, 48.5, 63.3, 68.6, 74.9 (q, *J* = 35.4 Hz), 122.4 (q, *J* = 279.8 Hz), 128.3, 128.8, 128.9, 134.2, 164.5, 167.1, 168.2. ^{19}F NMR (282.65 MHz, $CDCl_3$): δ –79.55 (d, *J* = 4.8 Hz). HRMS (FAB+) *m/z*: $[M+H]^+$ calcd for $C_{16}H_{16}F_3O_6$, 361.0893; found 361.0909.

5-Ethyl *anti,syn*-tetrahydro-2-oxo-3-(trifluoromethyl)furan-4,5-dicarboxylate (*anti,syn*-8a) and 4,5-diethyl *anti,syn*-tetrahydro-2-oxo-3-(trifluoromethyl)furan-4,5-dicarboxylate (*anti,syn*-7b). A mixture of ***anti,syn*-7a** and ***anti,syn*-7b** (1.3322 g, 3.83 mmol) was added to a two-necked flask containing 0.1969 g (0.19 mmol) of 10% Pd/C and AcOEt (19 mL) and the resultant mixture was stirred for 3 h at room temperature under a hydrogen atmosphere. Filtration of the mixture through Celite and evaporation of the volatiles, the crude materials were purified by silica gel column chromatography using Hex:AcOEt = 1:1 to AcOEt:MeOH = 4:1 to furnish 0.791 g (2.93 mmol) in 79% yield as an inseparable 97:3 diastereomer mixture. 13% of ***anti,syn*-7b** was at the same time recovered. ***anti,syn*-8a**: Rf = 0.21 (AcOEt:MeOH = 4:1). mp 166.2 °C. ^{1}H NMR (300.40 MHz, acetone-d_6): δ 1.28 (3H, t, *J* = 7.2 Hz), 4.17 (1H, d, *J* = 11.1 Hz), 4.27 (2H, q, *J* = 7.2 Hz), 4.46 (1H, dd, *J* = 8.4, 11.1 Hz), 5.61 (1H, m). ^{13}C NMR (75.45 MHz, acetone-d_6): δ 14.3, 45.2, 47.7, 63.3, 74.6 (q, *J* = 33.5 Hz), 124.2 (q, *J* = 281.6 Hz), 166.3, 168.0, 169.0. ^{19}F NMR (282.65 MHz, $CDCl_3$): δ –75.51 (d, *J* = 7.1 Hz; major), –79.43 (d, *J* = 4.5 Hz; minor). IR (KBr) ν 3193, 3001, 2970, 2922, 2535, 1805, 1746, 1708, 1376, 1317, 1181, 1146, 1067, 1038, 685 cm^{-1}. HRMS (FAB+) *m/z*: $[M+H]^+$ calcd for $C_9H_{10}F_3O_6$, 271.0424; found 271.0422. ***anti,syn*-7b**: Rf = 0.34 (Hex:AcOEt = 4:1). ^{1}H NMR (300.40 MHz, $CDCl_3$): δ 1.31 (3H, t, *J* = 7.2 Hz), 1.35 (3H, t, *J* = 7.2 Hz), 4.18–4.35 (6H, m), 5.07 (1H, dquint, *J* = 2.1, 6.6 Hz). ^{13}C NMR (75.45 MHz, $CDCl_3$): δ 13.6, 13.8, 44.4, 46.4 (q, *J* = 1.2 Hz), 62.6, 63.0, 73.5 (q, *J* = 3.3 Hz), 122.5 (q, *J* = 282.2 Hz), 165.2, 165.7, 167.6. ^{19}F NMR (282.65 MHz, $CDCl_3$): δ –75.90 (d, *J* = 6.8 Hz; major), –79.60 (d, *J* = 4.5 Hz; minor

(***syn*,*syn***)). IR (neat) ν 2989, 1814, 1742, 1373, 1322, 1225, 1184, 1129, 1068, 1026, 962, 858 cm^{-1}. HRMS (FAB+) *m/z*: $[M+H]^+$ calcd for $C_{11}H_{14}F_3O_6$, 299.0737; found 299.0739.

4,5-Dibenzyl *anti*,*syn*-tetrahydro-2-oxo-3-(trifluoromethyl)furan-4,5-dicarboxylate (*anti*,*syn*-7c). Following to **GP-3**, 0.142 g (0.50 mmol) of dibenzyl malonate [7] was used instead of the corresponding diethyl ester, and the same workup process and purification furnished 0.1333 g (0.32 mmol) of ***anti*,*syn*-7c** in 53% yield as an inseparable 98:2 diastereomer mixture. Rf = 0.20 (Hex:AcOEt = 8:1). ^{1}H NMR (300.40 MHz, $CDCl_3$): δ 4.20–4.31 (2H, m), 4.98 (1H, quint, *J* = 6.9 Hz), 5.10 (1H, d, *J* = 12.0 Hz), 5.18 (1H, d, *J* = 12.0 Hz). 5.20 (1H, d, *J* = 12.6 Hz), 5.25 (1H, d, *J* = 12.3 Hz), 7.30–7.34 (10H, m). ^{13}C NMR (75.45 MHz, $CDCl_3$): δ 44.4, 46.3, 68.48, 68.54, 73.4 (q, *J* = 33.5 Hz), 122.5 (q, *J* = 282.8 Hz), 128.0, 128.56, 128.59, 128.6, 128.7, 128.8, 134.0, 134.4, 165.0, 165.5 167.1. ^{19}F NMR (282.65 MHz, $CDCl_3$): δ –75.84 (d, *J* = 7.1 Hz; major), –79.51 (d, *J* = 4.5 Hz; minor). IR (neat) ν 3067, 3025, 2959, 1814, 1743, 1499, 1456, 1382, 1320, 1217, 1178, 1127, 1002, 753, 698 cm^{-1}. HRMS (FAB+) *m/z*: $[M+H]^+$ calcd for $C_{21}H_{18}F_3O_6$, 423.1050; found 423.1055.

Benzyl *syn*-5-amino-4-cyano-2-(trifluoromethyl)-2,3-dihydrofuran-3-carboxylate (*syn*-7d). Following to **GP-3**, 0.0402 mL (0.60 mmol) of malononitrile was reacted with 0.0741 g (0.30 mmol) of **2b**, and the same workup process and purification furnished 0.0681 g (0.22 mmol) of ***anti*-7d** in 73% yield as a single stereoisomer. Rf = 0.20 (Hex:AcOEt = 2:1). mp 117.7 °C. ^{1}H NMR (300.40 MHz, $CDCl_3$): δ 4.21(1H, d, *J* = 9.9 Hz), 5.02 (1H, dq, *J* = 6.6, 9.9 Hz), 5.15 (1H, d, *J* = 12.3 Hz), 5.20 (1H, d, *J* = 12.3 Hz), 5.32 (2H, brs), 7.31–7.41 (5H, m). ^{13}C NMR (75.45 MHz, $CDCl_3$): δ 47.5, 53.4, 68.2, 79.2 (q, *J* = 34.8 Hz), 116.6, 121.8 (q, *J* = 280.3 Hz), 128.5, 128.6, 134.6, 167.2, 168.0. ^{19}F NMR (282.65 MHz, $CDCl_3$): δ –75.00 (d, *J* = 6.8 Hz). IR(KBr) ν 3448, 3355, 3296, 3226, 3193, 3006, 2201, 1739, 1697, 1604, 1442, 1292, 1211, 1136, 1050, 968, 955, 749, 695 cm^{-1}. HRMS (FAB+) *m/z*: [M+H]+ calcd for $C_{14}H_{12}F_3N_2O_3$, 313.0795; found 313.0802.

3-Benzyl 4-ethyl *syn*-5-amino-2-(trifluoromethyl)-2,3-dihydrofuran-3,4-di-carboxylate (*syn*-7e). Following to **GP-3**, 0.11 mL (1.00 mmol) of ethyl 2-cyano-acetate, 0.1122 g (1.00 mmol) of *t*-BuOK, and 0.2462 g (1.00 mmol) of **2b** were used. After quenching the reaction, the obtained crude material was dissolved in AcOEt (3.0 mL) and the resultant mixture was refluxed for 15 h. After concentration and decantation by the addition of AcOEt, 0.2037 g (0.57 mmol) of the desired compound ***syn*-7e** was obtained in 57% total yield. Rf = 0.31 (Hex:AcOEt = 2:1). mp 116.0 °C. ^{1}H NMR (300.40 MHz, $CDCl_3$): δ 1.11 (3H, brt, *J* = 6.9 Hz), 4.04 (2H, brq, *J* = 7.2 Hz), 4.20 (1H, d, *J* = 9.9 Hz), 4.98 (1H, qd, *J* = 6.6, 9.9 Hz), 5.16 (2H, s), 5.85 (2H, brs), 7.29–7.41 (5H, m). ^{13}C NMR (75.45 MHz, $CDCl_3$): δ 14.3, 46.6, 59.1, 67.3, 75.1, 78.7 (q, *J* = 34.1 Hz), 122.1 (q, *J* = 279.7 Hz), 128.22, 128.28, 128.4, 136.2, 166.5, 169.6. ^{19}F NMR (282.65 MHz, $CDCl_3$): δ –74.82 (d, *J* = 6.2 Hz). IR (KBr) ν 3426, 3263, 3231, 3152, 1614, 1585, 1488,

1236, 1043, 755, 695, 547 cm^{-1}. HRMS (FAB+) *m/z*: $[M]^+$ calcd for $C_{16}H_{16}F_3NO_5$, 359.0975; found 359.0976.

Benzyl 2,3-*anti*-2-{cyano(ethoxycarbonyl)methyl}-4,4,4-trifluoro-3-hydroxy-butanoate (*anti*-9e). Following to **GP-3**, 0.032 mL (0.30 mmol) of ethyl 2-cyanoacetate was reacted with 0.0741 g (0.30 mmol) of **2b**, and the same workup process and purification furnished 0.0786 g (0.22 mmol) of ***anti*-9e** in 76% yield as an inseparable 90:10 diastereomer mixture. Rf = 0.40 (Hex:AcOEt = 2:1). IR (neat) ν 3426, 3379, 3151, 2906, 1742, 1582, 1489, 1473, 1334, 1321, 1287, 1240, 1214 cm^{-1}. HRMS (FAB+) *m/z*: $[M]^+$ calcd for $C_{16}H_{16}F_3NO_4$, 359.0981; found 359.0988. **major isomer:** ^{1}H NMR (300.40 MHz, $CDCl_3$): δ 1.28 (3H, t, *J* = 7.2 Hz), 3.42 (1H, dd, *J* = 2.7, 10.5 Hz), 3.64 (1H, d, *J* = 9.9 Hz), 4.22 (2H, q, *J* = 7.2 Hz), 4.24 (1H, d, *J* = 10.5 Hz) 4.46 (1H, m), 5.16 (1H, d, *J* = 12.3 Hz), 5.24 (1H, d, *J* = 12.0 Hz), 7.33–7.42 (5H, m). ^{13}C NMR (75.45 MHz, $CDCl_3$): δ 13.8, 37.2, 43.8, 63.8, 68.4, 69.5 (q, *J* = 32.2 Hz), 113.9, 123.6 (q, *J* = 282.8 Hz), 128.51, 128.54, 128.7, 134.1, 163.8, 168.8. ^{19}F NMR (282.65 MHz, $CDCl_3$): δ –78.19 (d, *J* = 6.8 Hz). **minor isomer:** ^{1}H NMR (300.40 MHz, $CDCl_3$): δ 1.33 (3H, t, *J* = 7.2 Hz), 3.53 (1H, dd, *J* = 3.9, 7.5 Hz), 4.03 (2H, q, *J* = 9.6 Hz), 4.07 (1H, d, *J* = 3.0 Hz). ^{13}C NMR (75.45 MHz, $CDCl_3$): δ 13.7, 36.4, 44.4, 63.8, 68.6, 113.8, 123.6 (q, *J* = 282.8 Hz), 128.6, 128.8, 134.0, 163.6, 168.6. ^{19}F NMR (282.65 MHz, $CDCl_3$): δ –78.02 (d, *J* = 6.8 Hz).

Benzyl 2,3-*anti*-4,4,4-trifluoro-2,3-dihydroxybutanoate (*anti*-10a). Following to **GP-3**, 0.1231 g (0.50 mmol) of **2b** was reacted with 0.051 mL (1.00 mmol) of nitromethane, and the same workup process and purification furnished 0.0223 g (0.085 mmol) of ***anti*-10a** in 17% yield as a sole stereoisomer. Rf = 0.21 (AcOEt:MeOH = 4:1). mp 76.3 °C. ^{1}H NMR (300.40 MHz, $CDCl_3$): δ 3.04 (2H, brs), 4.30 (1H, dq, *J* = 3.9, 6.9 Hz), 4.50 (1H, d, *J* = 3.0 Hz), 5.20 (1H, d, *J* = 12.3 Hz), 5.29 (1H, d, *J* = 12.0 Hz), 7.34–7.40 (5H, m). ^{13}C NMR (75.45 MHz, $CDCl_3$): δ 68.5, 71.1 (q, *J* = 1.3 Hz), 71.3 (q, *J* = 30.4 Hz), 123.8 (q, *J* = 283.5 Hz), 128.6, 128.7, 128.9, 134.2, 170.3. ^{19}F NMR (282.65 MHz, $CDCl_3$): δ –75.82 (d, *J* = 6.8 Hz). IR (KBr) ν 3356, 2999, 2893, 1752, 1499, 1285, 1152, 955, 824, 754, 638 cm^{-1}. HRMS (FAB+) *m/z*: $[M+Na]^+$ calcd for $C_{11}H_{11}F_3NaO_4$, 287.0502; found 287.0500.

General procedure for the reaction of the epoxyester 3a with cuprates (GP-4): 1,1,1-Trifluoro-2-hydroxytetradecan-4-one (11a). 1.70 mL of a 0.94 M Et_2O solution of decylmagnesium bromide (1.6 mmol) was added to an Et_2O (3.0 mL) solution containing 0.1524 g (0.80 mmol) of CuI at –40 °C under an argon atmosphere and the resultant mixture was stirred for 0.5 h at that temperature. A solution of 0.1231 g (0.50 mmol) of **2b** in Et_2O (1.0 mL) was added and the mixture was stirred for 3 h at the same temperature. After quenching the reaction with a saturated NH_4Cl aq, the usual workup afforded 0.1116 g (0.40 mmol) of the title compound in 79% yield after silica gel column chromatography using Hex:AcOEt = 6:1 as an eluent. Rf = 0.51 (Hex:AcOEt = 4:1). ^{1}H NMR (300.40 MHz, $CDCl_3$): δ 0.88 (3H, t, *J* = 6.9 Hz), 1.26 (14H, brs),

1.60 (2H, quint, J = 6.9 Hz), 2.49 (2H, t, J = 7.5 Hz), 2.74 (1H, dd, J = 3.6, 17.7 Hz), 2.83 (1H, dd, J = 9.0, 17.7 Hz), 3.49 (1H, d, J = 4.2 Hz), 4.43–4.56 (1H, m). ^{13}C NMR (75.45 MHz, $CDCl_3$): δ 14.0, 15.0, 22.6, 23.4, 29.26, 29.28, 29.4, 29.5, 31.8, 41.8 (q, J = 1.2 Hz), 43.7, 66.4 (q, J = 32.2 Hz), 124.7 (q, J = 281.1 Hz), 208.9. ^{19}F NMR (282.65 MHz, $CDCl_3$): δ –80.79 (d, J = 7.1 Hz). IR (neat) ν 3408, 2958, 2927, 2856, 1720, 1469, 1291, 1176, 1146, 899, 841, 719, 643 cm^{-1}. HRMS (FAB+) *m/z*: $[M+H]^+$ calcd for $C_{14}H_{26}F_3O_2$, 283.1879; found 283.1893.

6,6,6-Trifluoro-5-hydroxy-1-phenylhexan-3-one (11b) [8]**.** Following to the **GP4**, 2-phenylethylmagnesium bromide was used instead of decylmagnesium bromide. However, because separation of unidentified byproducts was not possible even after silica gel column chromatography, 57% yield was determined by ^{19}F NMR. Rf = 0.27 (Hex:AcOEt = 4:1). ^{1}H NMR (300.40 MHz, $CDCl_3$): δ 2.64 (1H, dd, J = 3.0, 17.7 Hz), 2.76–2.93 (5H, m), 3.32 (1H, brs), 4.42–4.53 (1H, m), 7.16–7.37 (5H, m). ^{13}C NMR (75.45 MHz, $CDCl_3$): δ 29.2, 42.0 (q, J = 1.3 Hz), 45.0, 66.3 (q, J = 32.2 Hz), 124.6 (q, J = 280.3 Hz), 126.3, 128.2, 128.5, 140.2, 207.7. ^{19}F NMR (282.65 MHz, $CDCl_3$): δ –80.80 (d, J = 7.1 Hz).

1-Cyclohexyl-4,4,4-trifluoro-3-hydroxybutan-1-one (11c) [9]**.** Following to the above general procedure, cyclohexylmagnesium bromide was used instead of decyl-magnesium bromide, and 0.0612 g (0.27 mmol) of the title compound was isolated in 55% yield. Rf = 0.41 (Hex:AcOEt = 4:1). ^{1}H NMR (300.40 MHz, $CDCl_3$): δ 1.19–1.91 (10H, m), 2.39 (1H, tt, J = 3.3, 11.1 Hz), 2.77 (1H, dd, J = 3.0, 17.7 Hz), 2.86 (1H, dd, J = 8.7, 17.7 Hz), 3.53 (1H, d, J = 4.2 Hz), 4.42–4.53 (1H, m). ^{13}C NMR (75.45 MHz, $CDCl_3$): δ 25.3, 25.4, 25.6, 27.9, 28.0, 39.9 (q, J = 1.3 Hz), 51.3, 66.4 (q, J = 32.4 Hz), 124.8 (q, J = 280.3 Hz), 211.9. ^{19}F NMR (282.65 MHz, $CDCl_3$): δ –80.70 (d, J = 6.8 Hz).

(*E*)-4-Decyl-2,3-epoxy-4,4,4-trifluorotetradecan-4-ol (12a). 1.70 mL of a 0.94 M Et_2O solution of decylmagnesium bromide (1.6 mmol) was added to a solution of 0.1231 g (0.50 mmol) of **2b** in Et_2O (4.0 mL) at –40 °C under an argon atmosphere, and the resultant mixture was stirred for 0.5 h at that temperature. After quenching the reaction with a saturated NH_4Cl aq., the usual workup afforded 0.1546 g (0.36 mmol) of the title compound in 73% yield after silica gel column chromatography using Hex:AcOEt = 20:1 as an eluent. Rf = 0.37 (Hex:AcOEt = 20:1). ^{1}H NMR (300.40 MHz, $CDCl_3$): δ 0.86–0.90 (6H, m), 1.26–1.62 (36H, m), 3.18 (1H, d, J = 1.5 Hz), 3.53 (1H, dq, J = 1.8, 5.1 Hz). ^{13}C NMR (75.45 MHz, $CDCl_3$): δ 14.1, 22.7, 22.8, 23.2, 29.3, 29.4, 29.5, 29.6, 30.1, 31.9, 36.5, 39.6, 50.4 (q, J = 40.3 Hz), 59.6 (q, J = 1.3 Hz), 70.5, 122.9 (q, J = 274.8 Hz). ^{19}F NMR (282.65 MHz, $CDCl_3$): δ –74.84 (d, J = 4.5 Hz). IR (neat) ν 3019, 2927, 2855, 1468, 1284, 1216, 1163, 932, 868, 760, 670 cm^{-1}. HRMS (FAB+) *m/z*: $[M+H]^+$ calcd for $C_{24}H_{46}F_3O_2$, 423.3444; found 423.3448.

(*E*)-2,3-Epoxy-4,4,4-trifluoro-1,1-diphenylbutan-1-ol (12d) [10]**.** Following to the **GP4**, phenylmagnesium bromide was used instead of decylmagnesium bromide, and 0.1143 g (0.39

mmol) of the title compound was isolated in 77% yield. Rf = 0.54 (Hex:AcOEt = 4:1). ^{1}H NMR (300.40 MHz, $CDCl_3$): δ 2.47 (1H, brs), 3.72 (1H, dq, *J* = 1.8, 5.1 Hz), 4.00 (1H, d, *J* = 1.8 Hz), 7.35–7.49 (10H, m). ^{13}C NMR (75.45 MHz, $CDCl_3$): δ 50.8 (q, *J* = 41.0 Hz), 59.9, 74.9, 122.6 (q, *J* = 275.3 Hz), 126.1, 127.0, 128.1, 128.3, 128.5, 128.7, 141.8, 143.6. ^{19}F NMR (282.65 MHz, $CDCl_3$): δ –74.62 (d, *J* = 4.5 Hz).

(*E*)-2,3-Epoxy-4,4,4-trifluoro-1,1-bis(4-methoxyphenyl)butan-1-ol (12e). Following to the **GP4**, 4-methoxyphenylmagnesium bromide was used instead of decyl-magnesium bromide, and 0.1417 g (0.40 mmol) of the title compound was isolated in 80% yield. Rf = 0.34 (Hex:AcOEt = 4:1). mp 105.1 °C. ^{1}H NMR (300.40 MHz, $CDCl_3$): δ 2.39 (1H, s), 3.70 (1H, dq, *J* = 2.1, 5.1 Hz), 3.80 (3H, s), 3.81 (3H, s), 3.90 (1H, d, *J* = 1.8 Hz), 6.84–6.92 (4H, m), 7.21–7.26 (2H, m), 7.35–7.40 (2H, m). ^{13}C NMR (75.45 MHz, $CDCl_3$): δ 50.8 (q, *J* = 41.0 Hz), 55.1, 55.2, 60.1 (q, *J* = 2.5 Hz), 74.3, 113.7, 113.8, 122.7 (q, *J* = 275.4 Hz), 127.4, 128.3, 134.3, 135.9, 159.1, 159.3. ^{19}F NMR (282.65 MHz, $CDCl_3$): δ –74.61 (d, *J* = 4.5 Hz). IR (KBr) ν 3514, 3033, 3013, 2963, 2938, 2915, 2842, 1610, 1586, 1512, 1469, 1254, 1172, 1033, 812, 679 cm^{-1}. HRMS (FAB+) *m/z*: $[M]^+$ calcd for $C_{18}H_{17}F_3O_4$, 354.1073, found 354.1070.

1,1,1-Trifluoro-2-hydroxydecan-4-one (11f) [11]**.** Following to the **GP4**, 0.1121 g (0.50 mmol) of 1,1,1-trifluoro-2,3-epoxydecan-4-one [12] was used instead of **2b** and the usual workup and purification afforded 0.0362 g (0.16 mmol) of the desired compound in 32% yield along with 0.0362 g (0.258 mmol) of 2,3-epoxy-1,1,1-trifluoro-4-hexyltetradecan-4-ol (**12f**) in 52% yield in a 98:2 isomeric ratio. Rf = 0.37 (Hex:AcOEt = 6:1). ^{1}H NMR (300.40 MHz, $CDCl_3$): δ 0.89 (3H, t, *J* = 6.9 Hz), 1.29 (6H, brs), 1.60 (2H, quint, *J* = 7.2 Hz), 2.49 (2H, t, *J* = 7.5 Hz), 2.74 (1H, dd, *J* = 3.0, 17.7 Hz), 2.84 (1H, dd, *J* = 8.7, 17.7 Hz), 3.73 (1H, d, *J* = 4.5 Hz), 4.44–4.56 (1H, m). ^{13}C NMR (75.45 MHz, $CDCl_3$): δ 13.9, 22.4, 23.3, 28.6, 31.4, 41.8, 43.7, 66.4 (q, *J* = 32.2 Hz), 124.7 (q, *J* = 280.3 Hz), 209.1. ^{19}F NMR (282.65 MHz, $CDCl_3$): δ –80.79 (d, *J* = 7.1 Hz).

2,3-Epoxy-1,1,1-trifluoro-4-hexyltetradecan-4-ol (12f). Rf = 0.59 (Hex:AcOEt = 6:1). ^{1}H NMR (300.40 MHz, $CDCl_3$): δ 0.86–0.93 (6H, m), 1.27–1.29 (24H, m), 1.50–1.60 (4H, m), 3.18 (1H, d, *J* = 2.1 Hz), 3.53 (1H, dq, *J* = 1.8, 5.1 Hz). ^{13}C NMR (75.45 MHz, $CDCl_3$): δ 14.0, 14.1, 22.5, 22.7, 22.8, 23.2, 29.3, 29.50, 29.56, 29.6, 29.7, 30.1, 31.7, 31.9, 36.5, 39.7, 50.4 (q, *J* = 40.3 Hz), 59.6 (q, *J* = 1.9 Hz), 70.5, 122.9 (q, *J* = 274.7 Hz). ^{19}F NMR (282.65 MHz, $CDCl_3$): δ –74.83 (d, *J* = 4.8 Hz). IR (neat) ν 3466, 2856, 1469, 1343, 1283, 1246, 1161, 935, 864, 691 cm^{-1}. HRMS (FAB+) *m/z*: $[M+H]^+$ calcd for $C_{20}H_{38}F_3O_2^+$, 367.2818; found 367.2820.

1,1,1-Trifluoro-3-deutero-2-hydroxytetradecan-4-one (11a-D). Following to the **GP-4**, the reaction mixture was quenched with 0.5 mL of D_2O, the mixture was stirred overnight at room temperature. The usual workup and purification afforded 0.1008 g (0.36 mmol) of the desired compound in 71% yield. Rf = 0.51 (Hex:AcOEt = 4:1), mp 53.1 °C. ^{1}H NMR (300.40 MHz, $CDCl_3$): δ 0.88 (3H, t, *J* = 6.9 Hz), 1.26 (14H, brs), 1.59 (2H, quint, *J* = 6.9 Hz), 2.48 (2H, t, *J* =

7.2 Hz), 2.72–2.82 (1H, m), 3.57–3.59 (1H, m), 4.48–4.52 (1H, m). ^{13}C NMR (75.45 MHz, $CDCl_3$): δ 14.0, 22.6, 23.4, 29.0, 29.26, 29.29, 29.4, 29.5, 31.8, 41.5 (t, *J* = 19.8 Hz), 43.7, 124.7 (q, *J* = 280.4 Hz), 208.9. ^{19}F NMR (282.65 MHz, $CDCl_3$): δ –80.82 (d, *J* = 6.8 Hz). IR (KBr) ν 3392, 2920, 2852, 1713, 1469, 1282, 1165, 1128, 939, 847, 703, 632, 560 cm^{-1}. HRMS (FAB+) *m/z*: $[M+H]^+$ calcd for $C_{14}H_{25}DF_3O_2^+$, 284.1942; found 284.1957.

(*E*)-2,3-Epoxy-4,4,4-trifluorotetradecan-4-one (13a). Following to the **GP4**, the reaction was performed for 1 h. After silica gel column chromatography using Hex:$CHCl_3$ = 4:1, the complete separation from **12a** was not possible but the following data were collected by using a pure fraction (19% yield determined by ^{19}F NMR). Rf = 0.17 (Hex:$CHCl_3$ = 4:1). ^{1}H NMR (300.40 MHz, $CDCl_3$): δ 0.88 (3H, t, *J* = 6.6 Hz), 1.26 (1H, m), 1.61 (2H, quint, *J* = 7.5 Hz), 2.39 (1H, td, *J* = 7.5, 17.4 Hz), 2.52 (1H, td, *J* = 7.2, 17.4 Hz), 3.59 (1H, dq, *J* = 1.8, 4.5 Hz), 3.69 (1H, d, *J* = 1.5 Hz). ^{13}C NMR (75.45 MHz, $CDCl_3$): δ 14.1, 22.6, 22.8, 29.0, 29.3, 29.4, 29.5, 31.9, 38.5, 52.9 (q, *J* = 42.2 Hz), 54.3 (q, *J* = 1.9 Hz), 63.1, 121.5 (q, *J* = 276.0 Hz), 203.1. ^{19}F NMR (282.65 MHz, $CDCl_3$): δ –75.13 (d, *J* = 4.5 Hz). IR ($CHCl_3$) ν 3025, 2955, 2928, 2856, 1725, 1465, 1286, 1216, 1168, 762 cm^{-1}. HRMS (FAB+) *m/z*: $[M]^+$ calcd for $C_{14}H_{23}F_3O_2$, 280.1645; found 280.1639.

Benzyl (*E*)-4-chloro-4,4-difluorobut-2-enoate (**1d**)

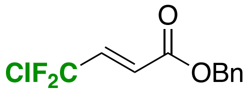

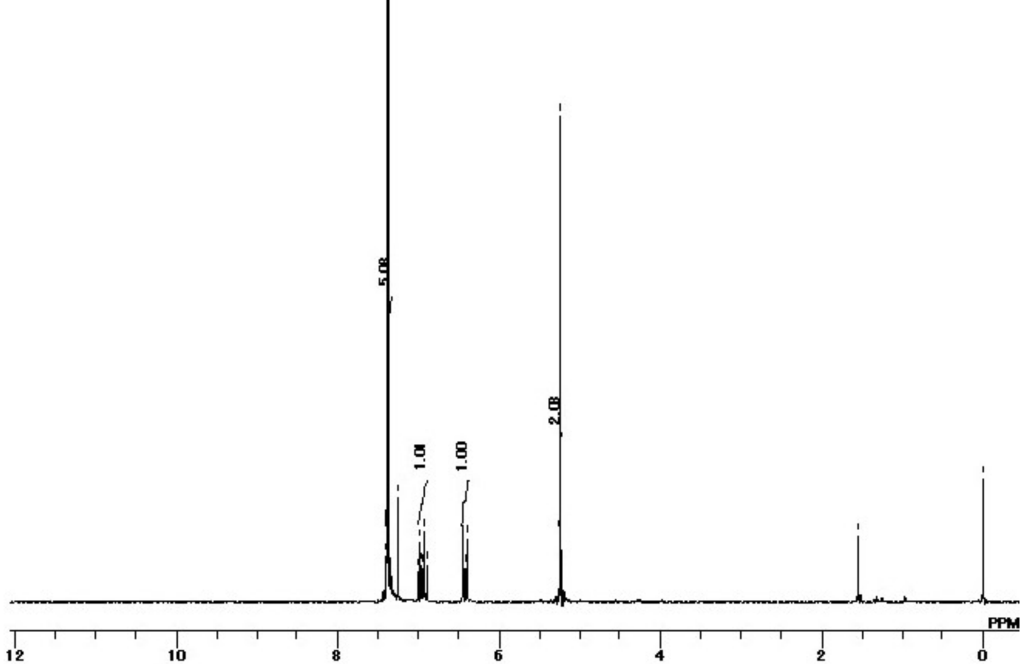

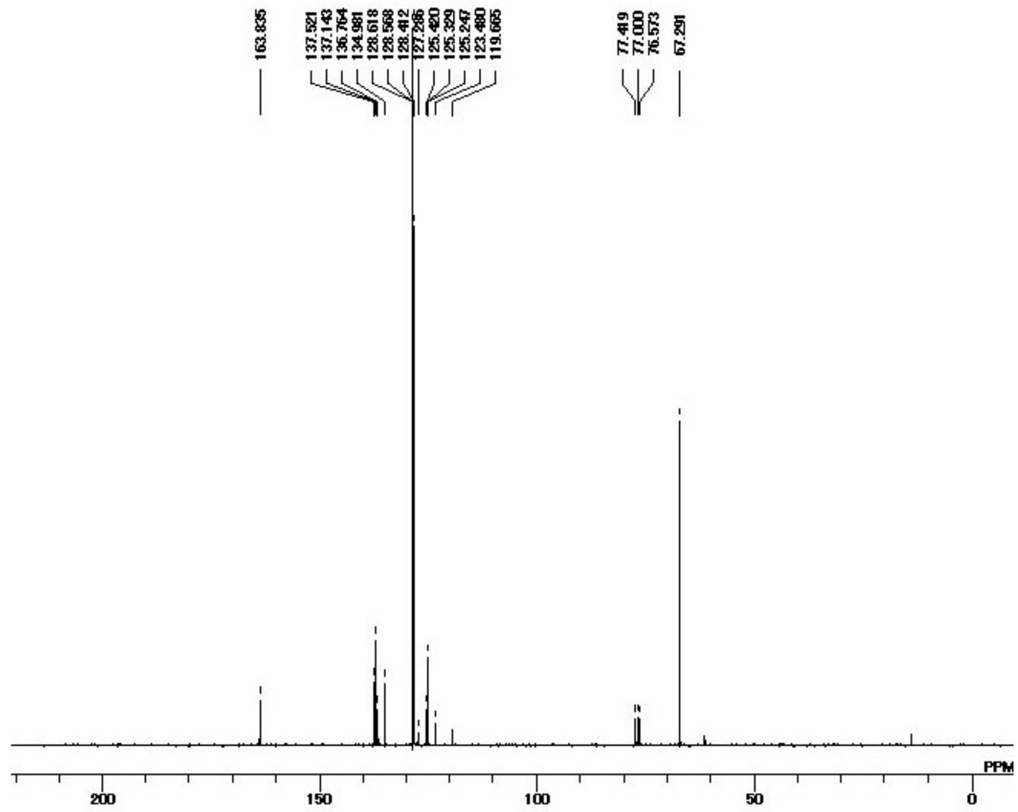

Benzyl (*E*)-2,3-epoxy-4,4,4-trifluorobutanoate (**2b**)

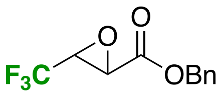

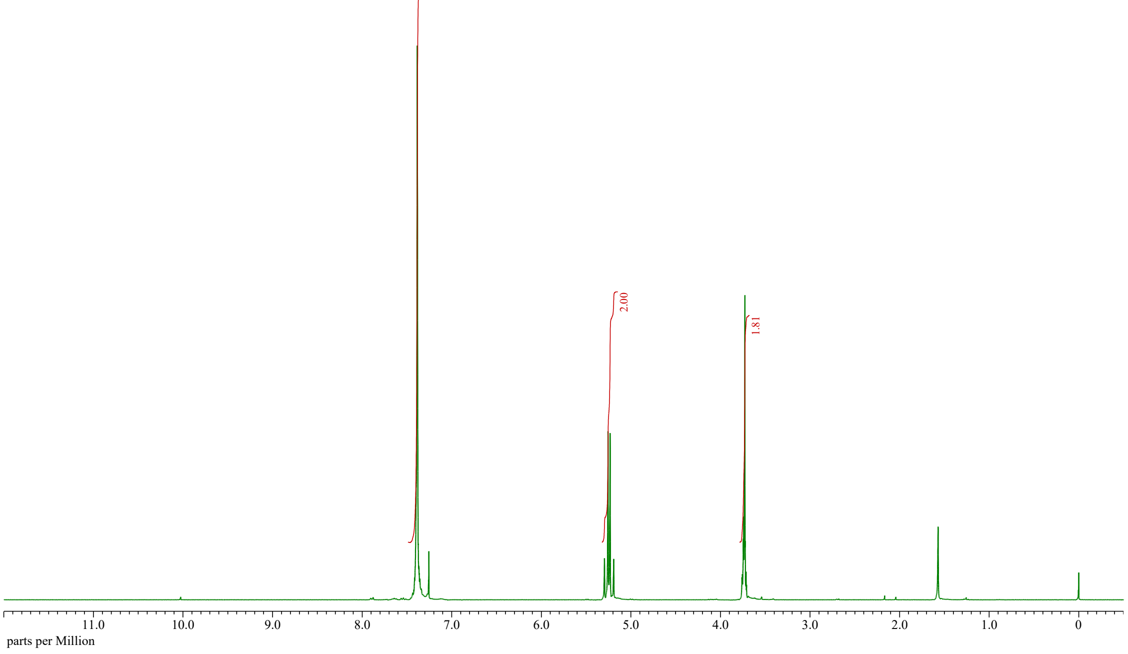

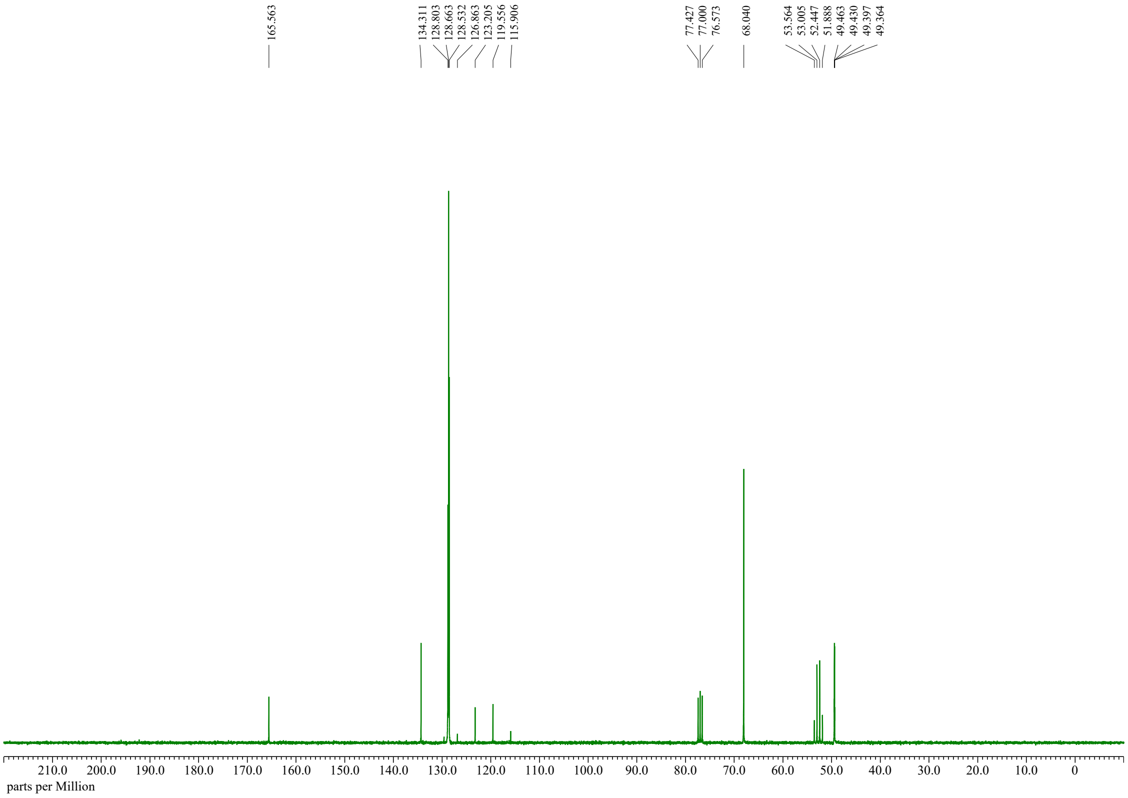

Benzyl (*E*)-2,3-epoxy-4,4-difluorobutanoate (**2c**)

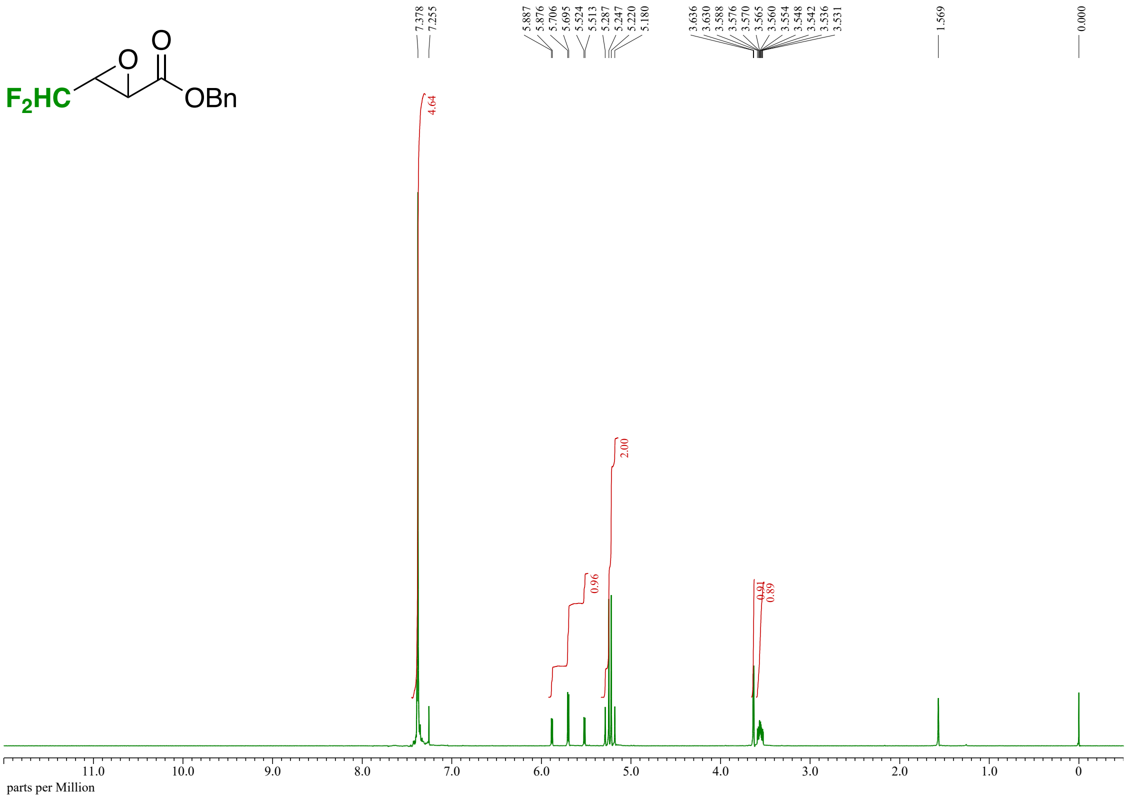

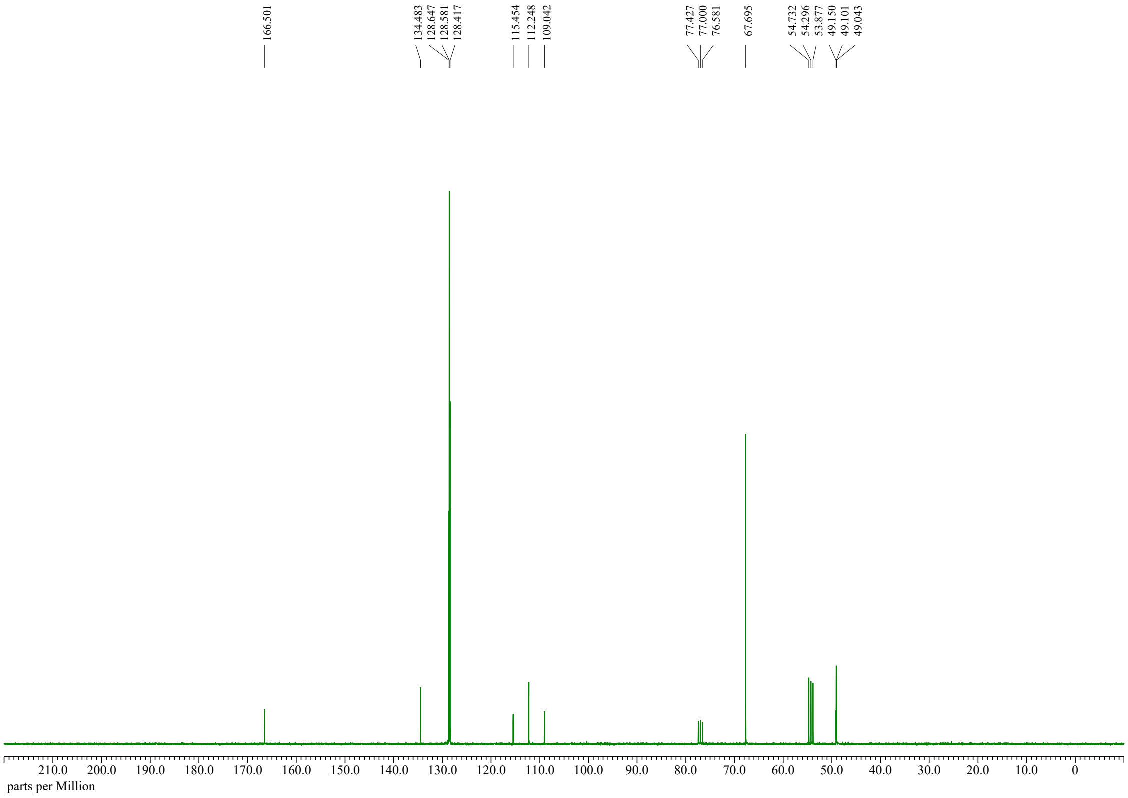

Benzyl (*E*)-4-chloro-2,3-epoxy-4,4-difluorobutanoate (**2d**)

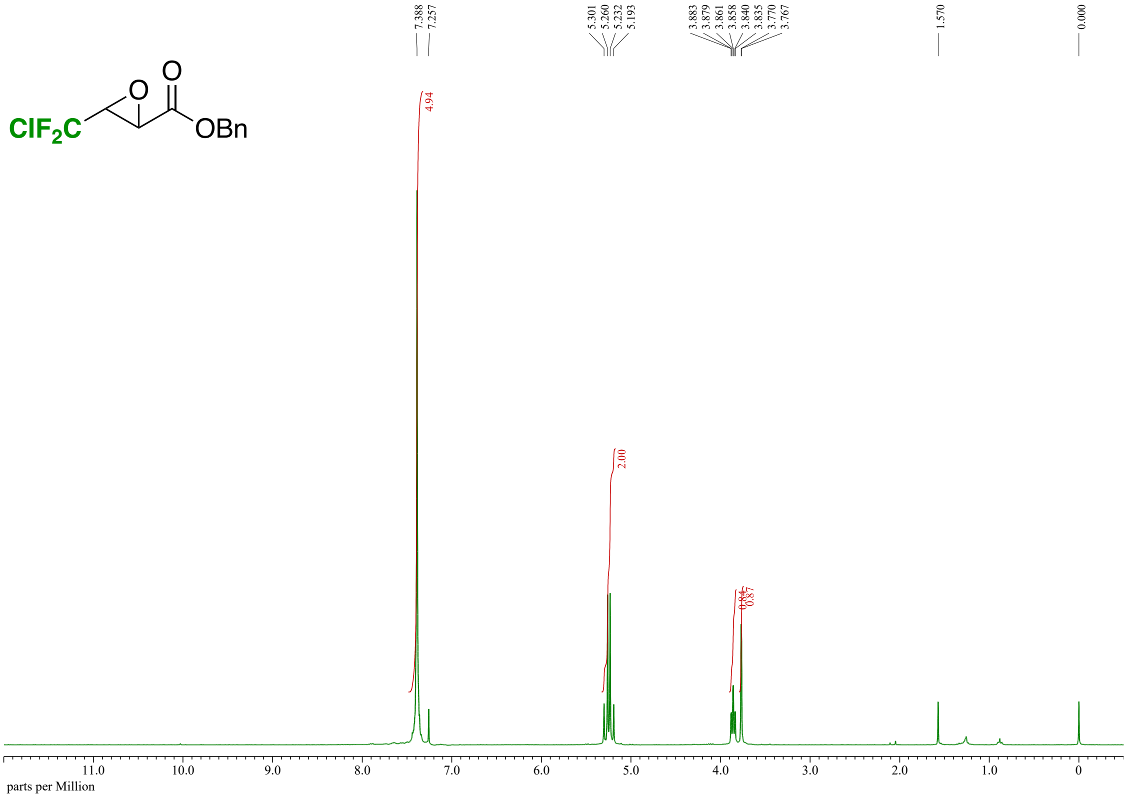

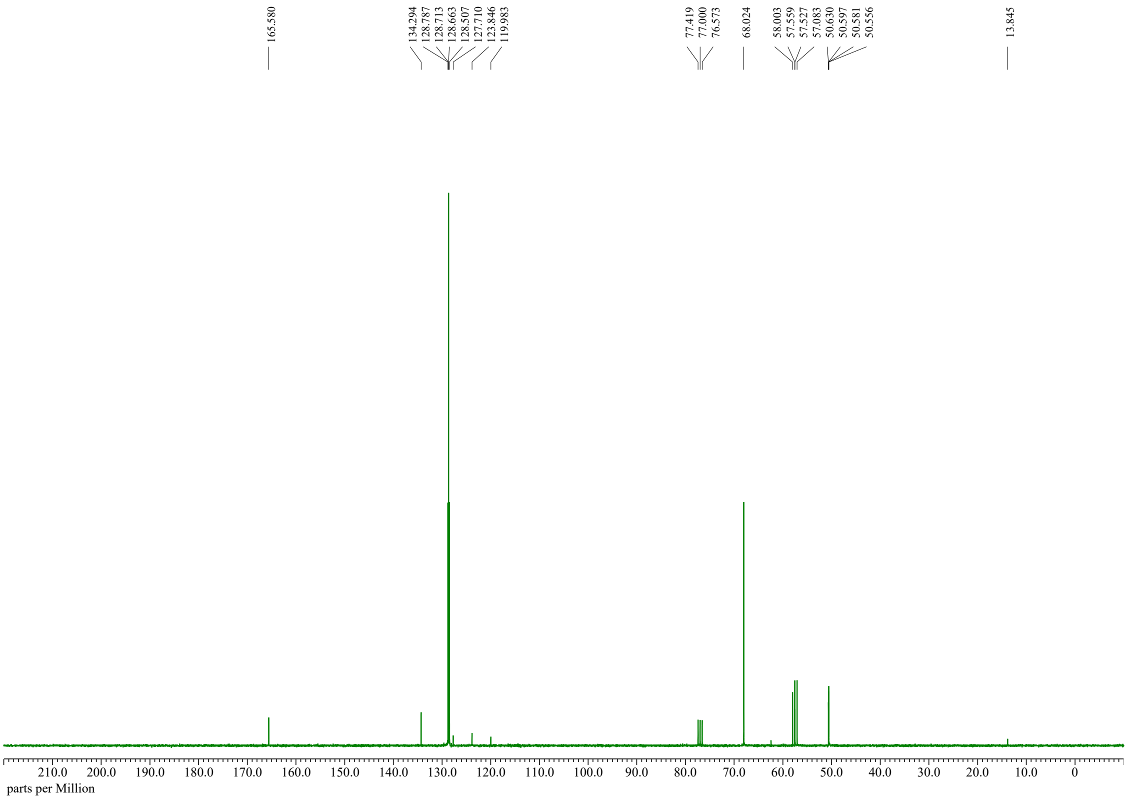

Benzyl (*E*)-2,3-epoxy-4,4,5,5,5-pentafluoropentanoate (**2e**)

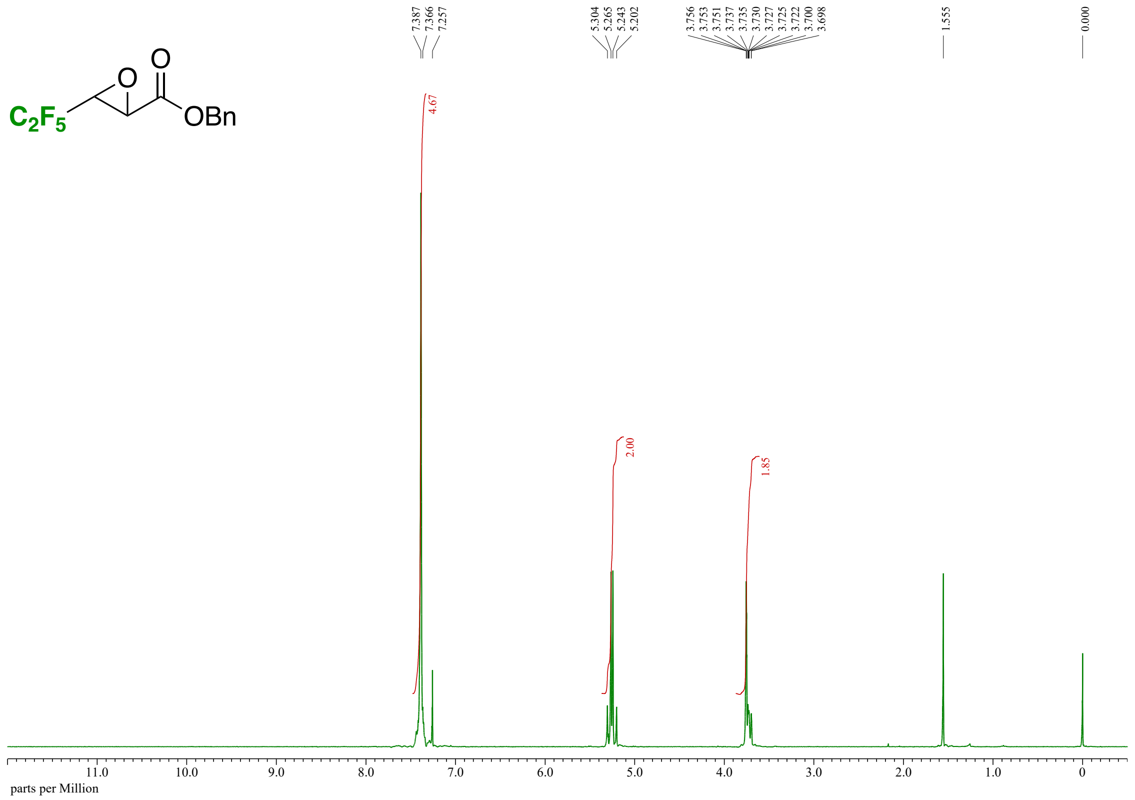

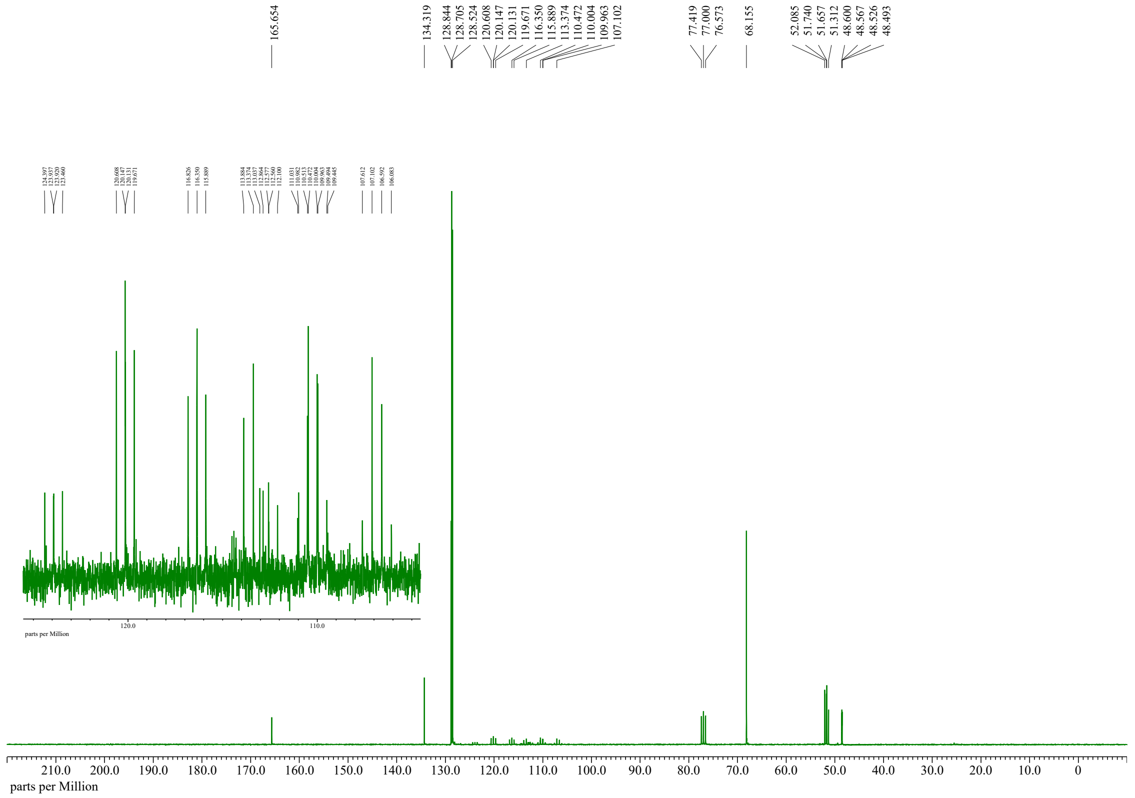

Ethyl (*E*)-2,3-epoxy-4,4,5,5,6,6,7,7,8,8,9,9,9-tridercafluorononanonate (**2f**)

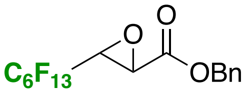

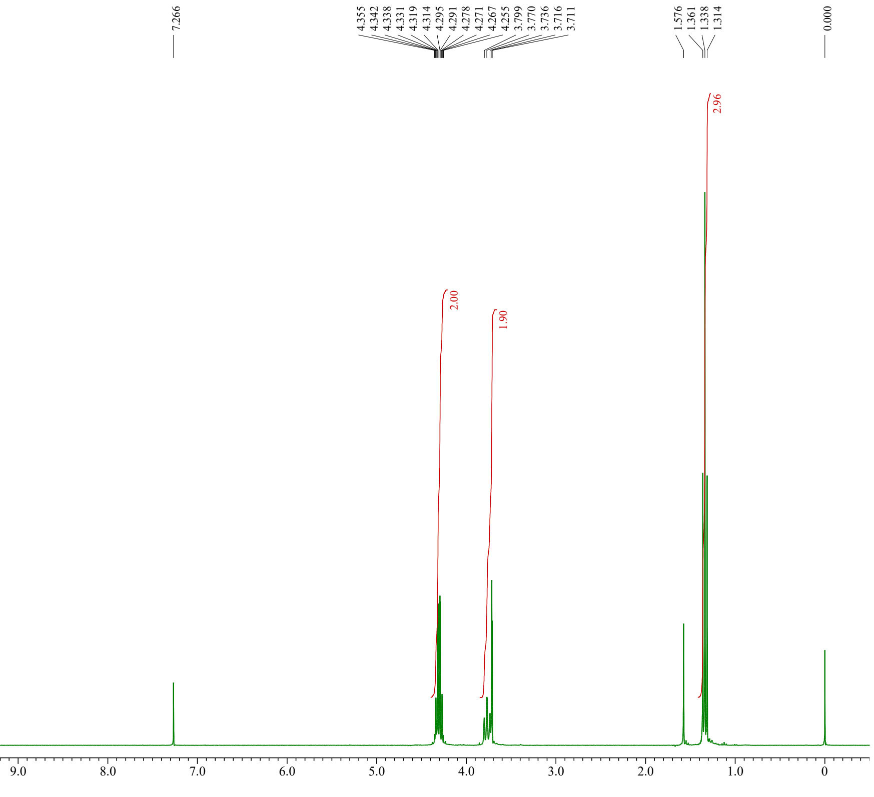

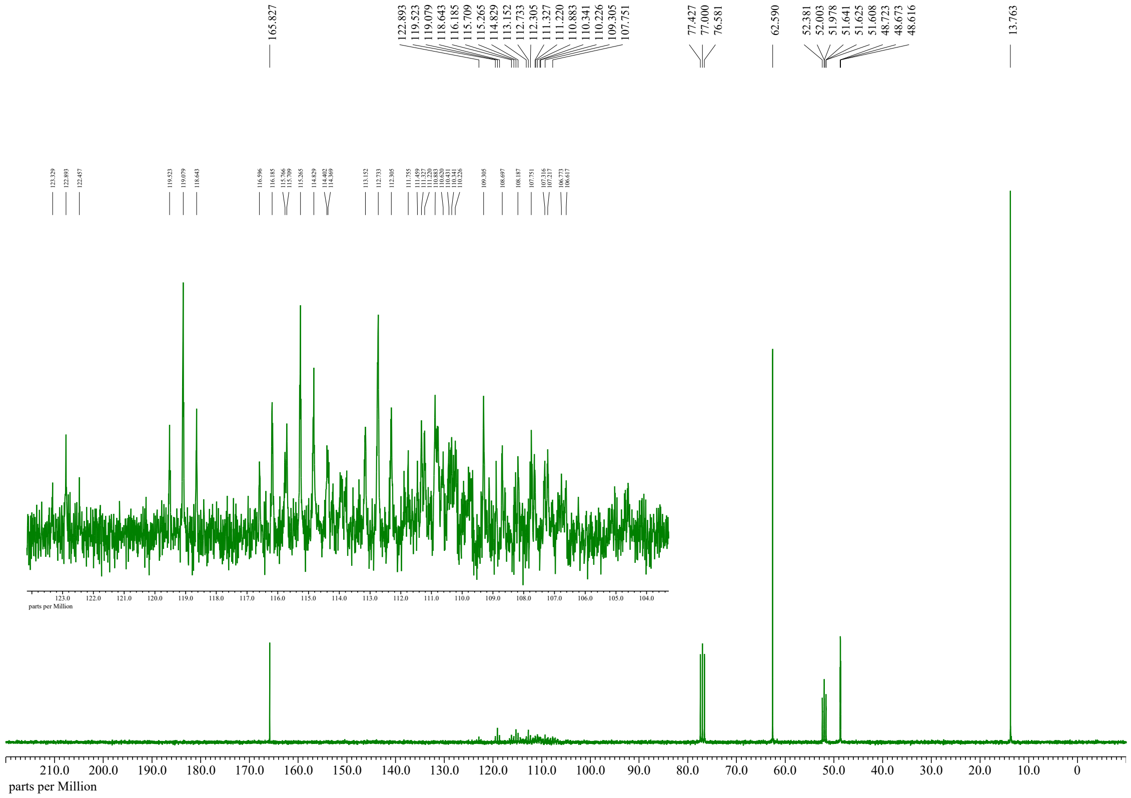

Benzyl 2,3-*anti*-4,4,4-trifluoro-3-hydroxy-2-(*p*-methoxyphenyl)aminobutanoate (**3ba**)

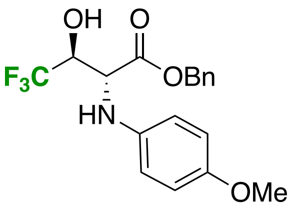

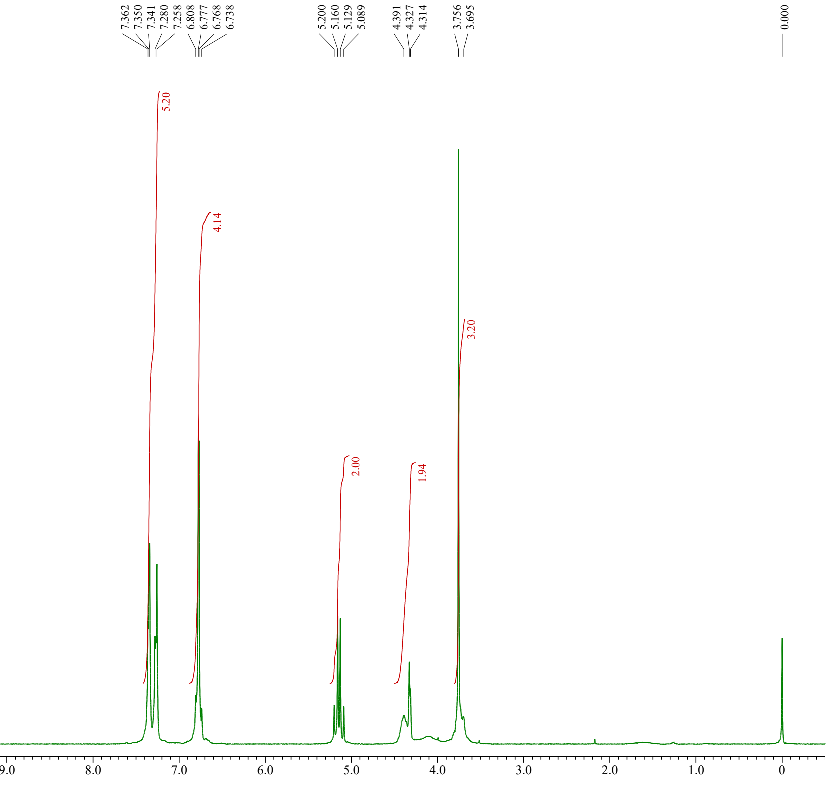

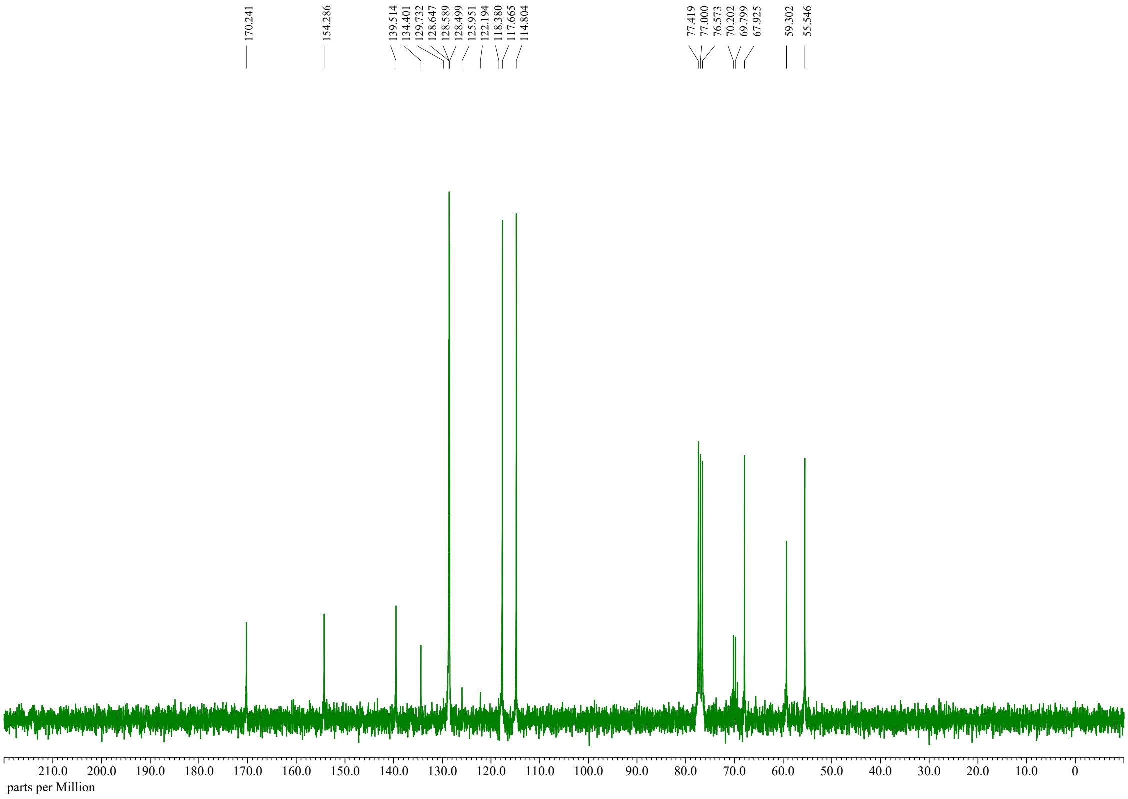

Benzyl 2,3-*anti*-4,4-difluoro-3-hydroxy-2-(*p*-methoxyphenyl)aminobutanoate (**3ca**)

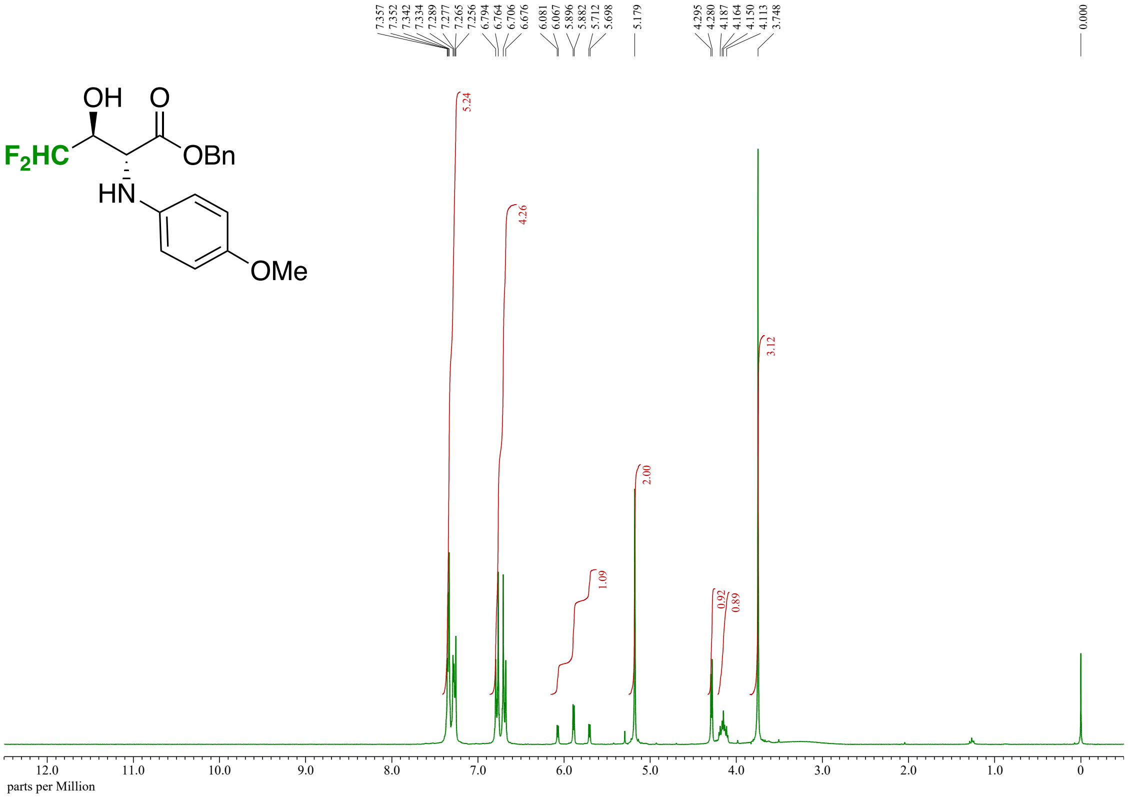

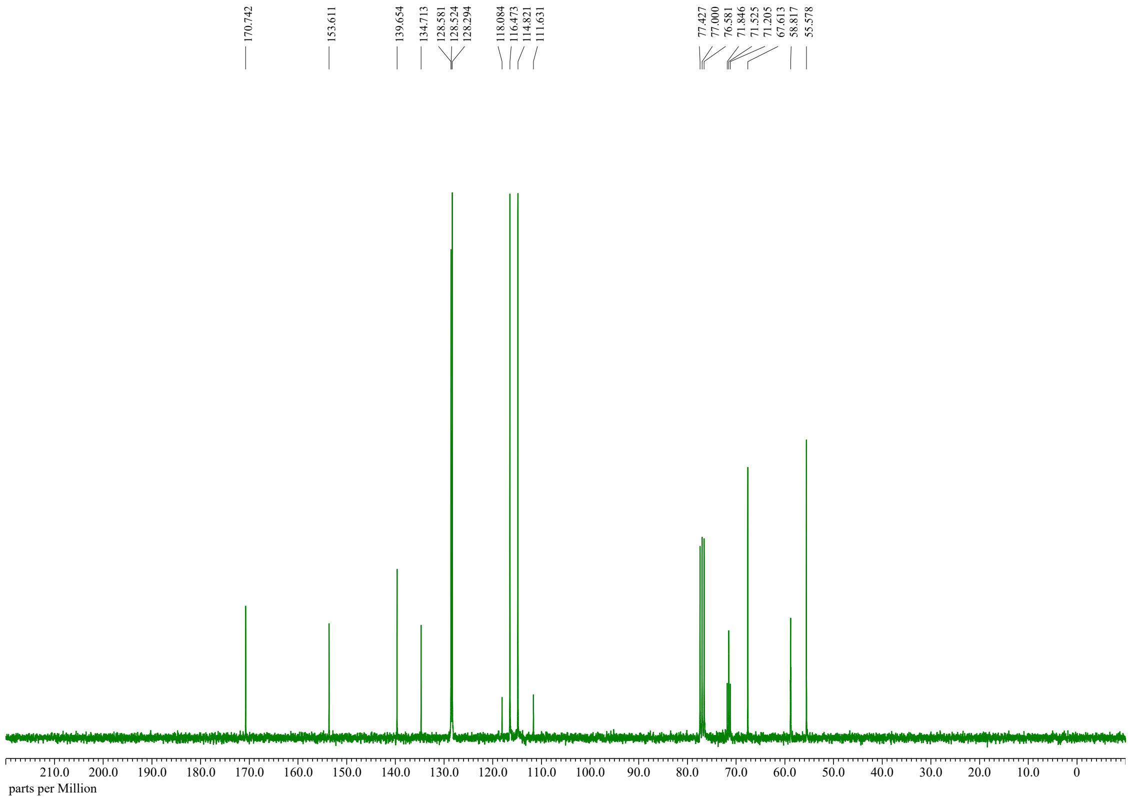

Benzyl 2,3-*anti*-4-chloro-4,4-difluoro-3-hydroxy-2-(*p*-methoxyphenyl)aminobutanoate (**3da**)

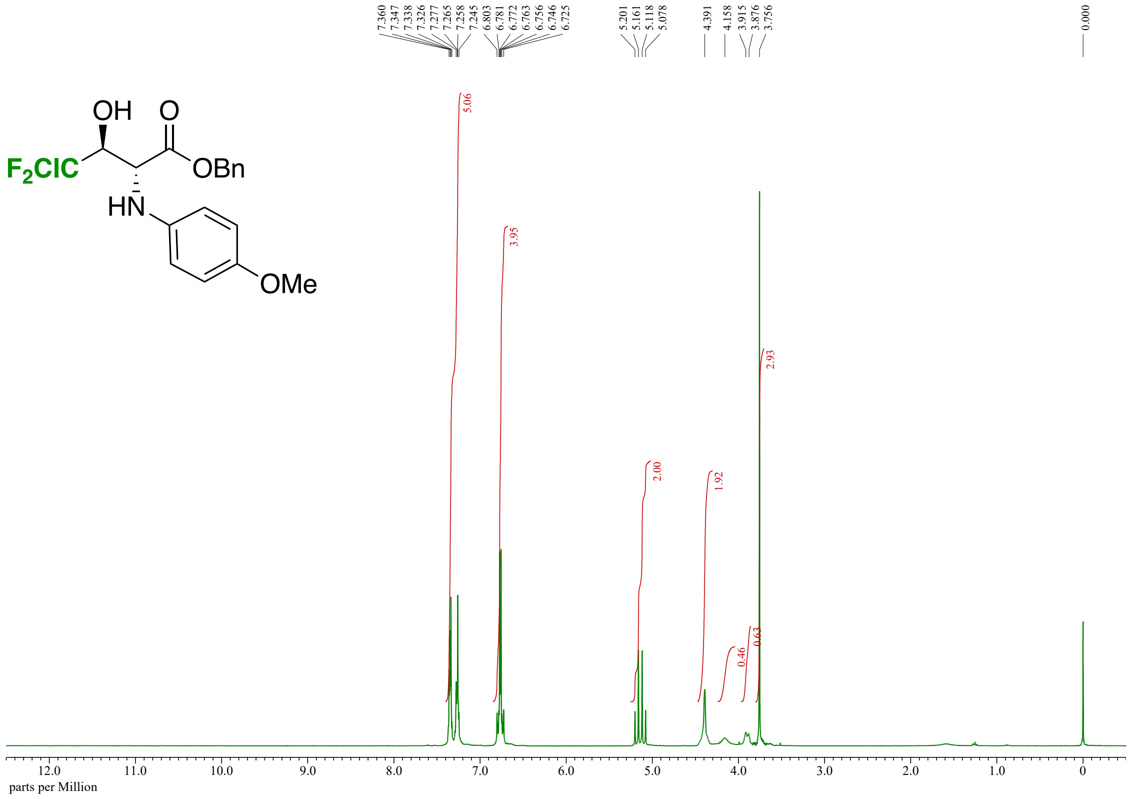

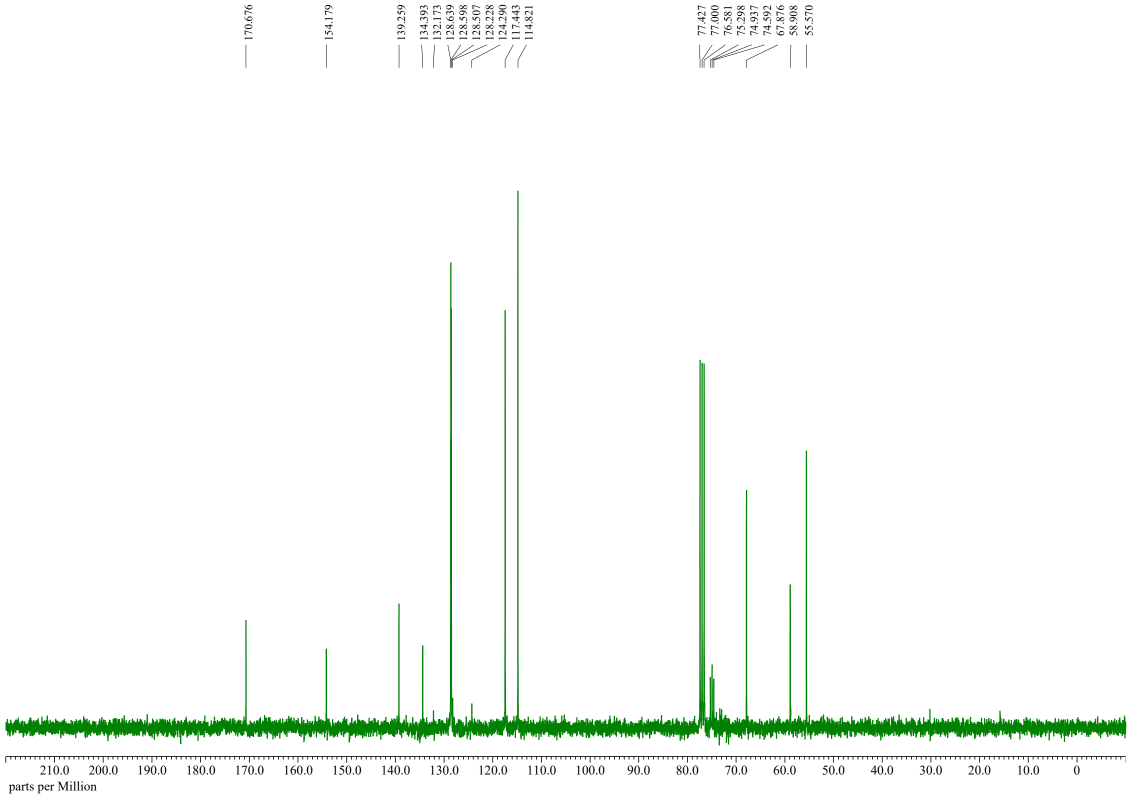

Benzyl 2,3-*anti*-2-(benzylamino)-4,4,4-trifluoro-3-hydroxybutanoate (**3bb**)

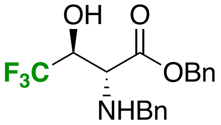

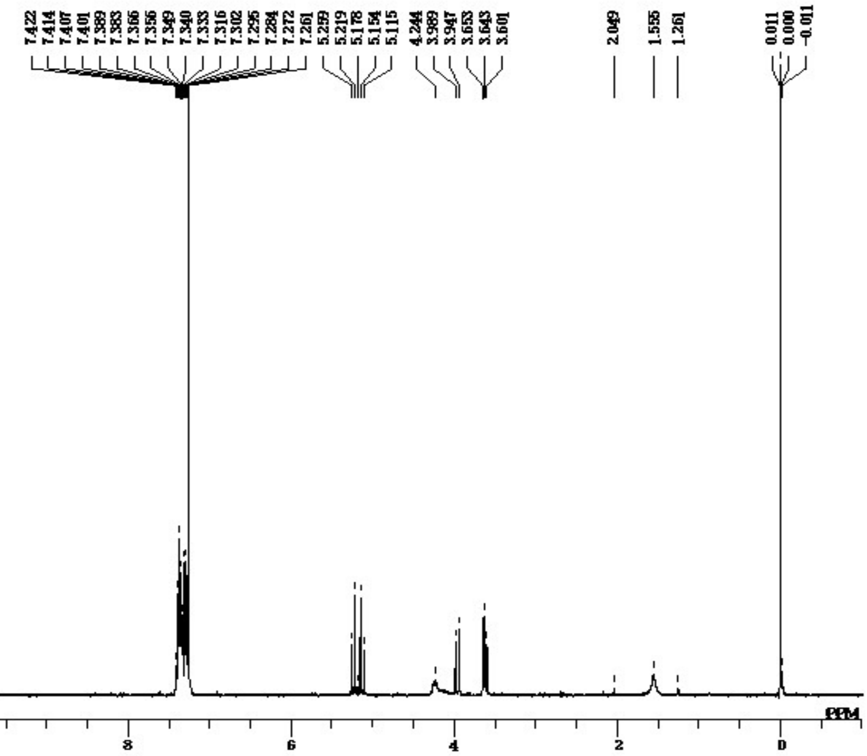

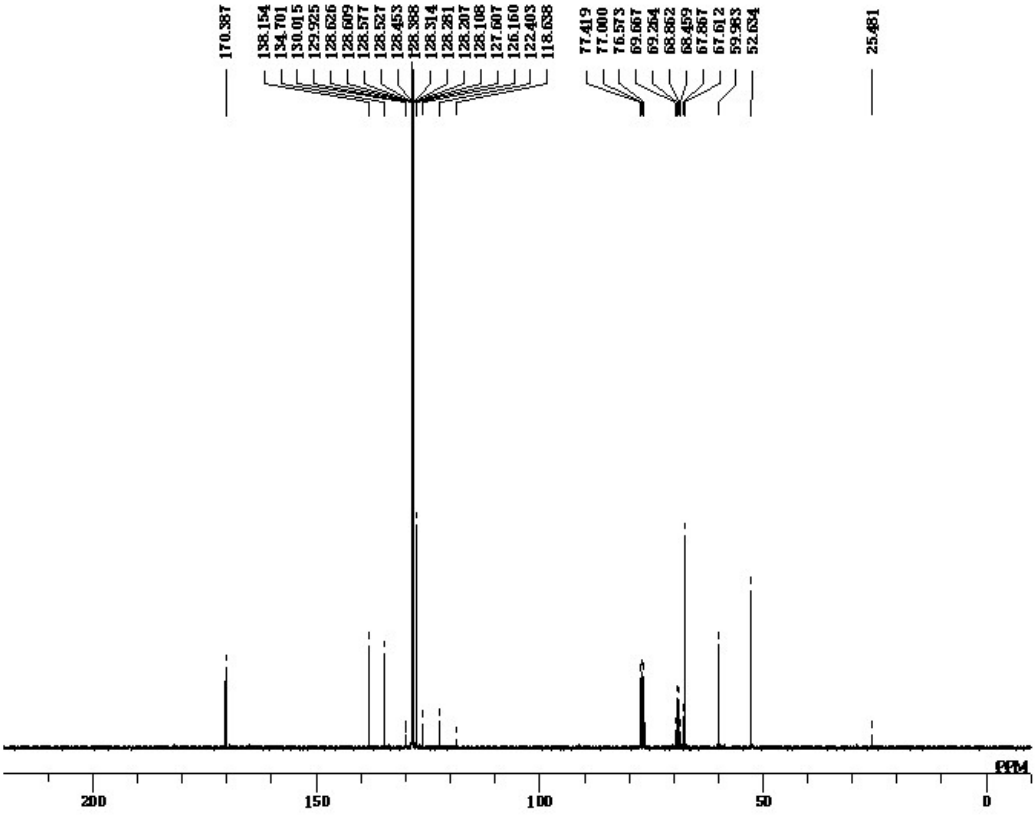

Benzyl 2,3-*anti*-2-(*n*-buthylamino)-4,4,4-trifluoro-3-hydroxybutanoate (**3bc**)

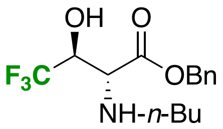

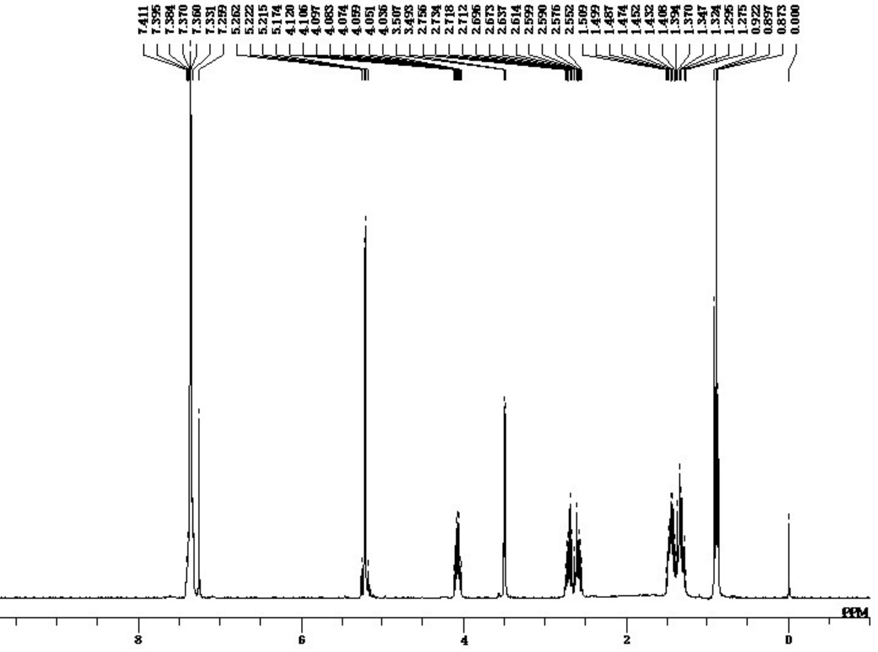

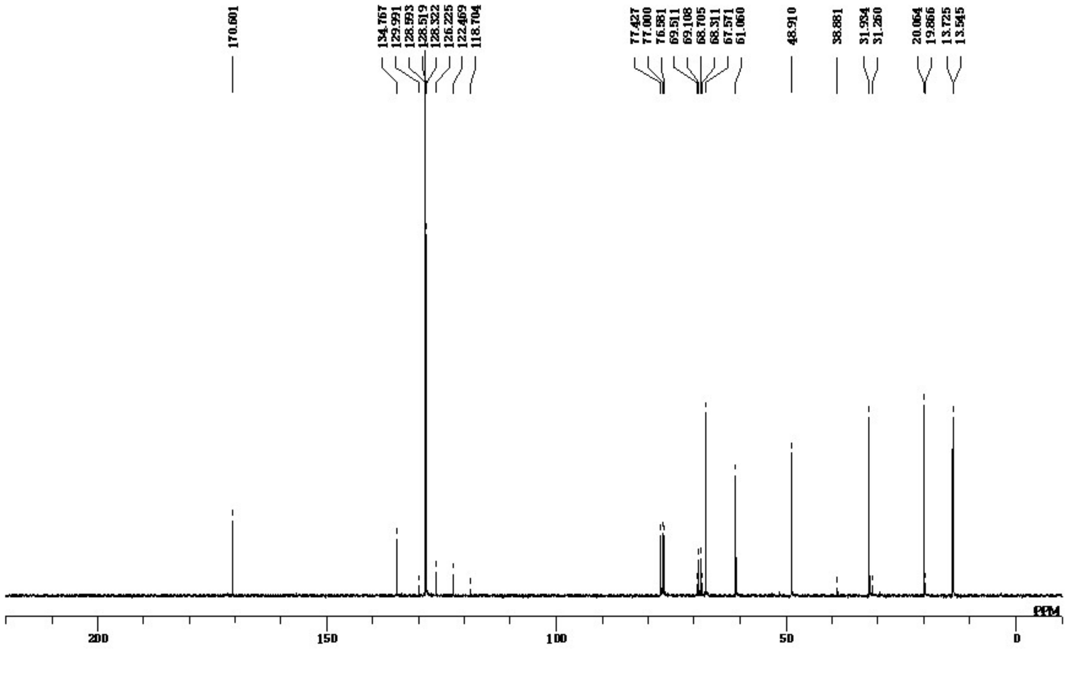

Benzyl 2,3-*anti*-2-(1-phenylethylamino)-4,4,4-trifluoro-3-hydroxybutanoate (**3bd**)

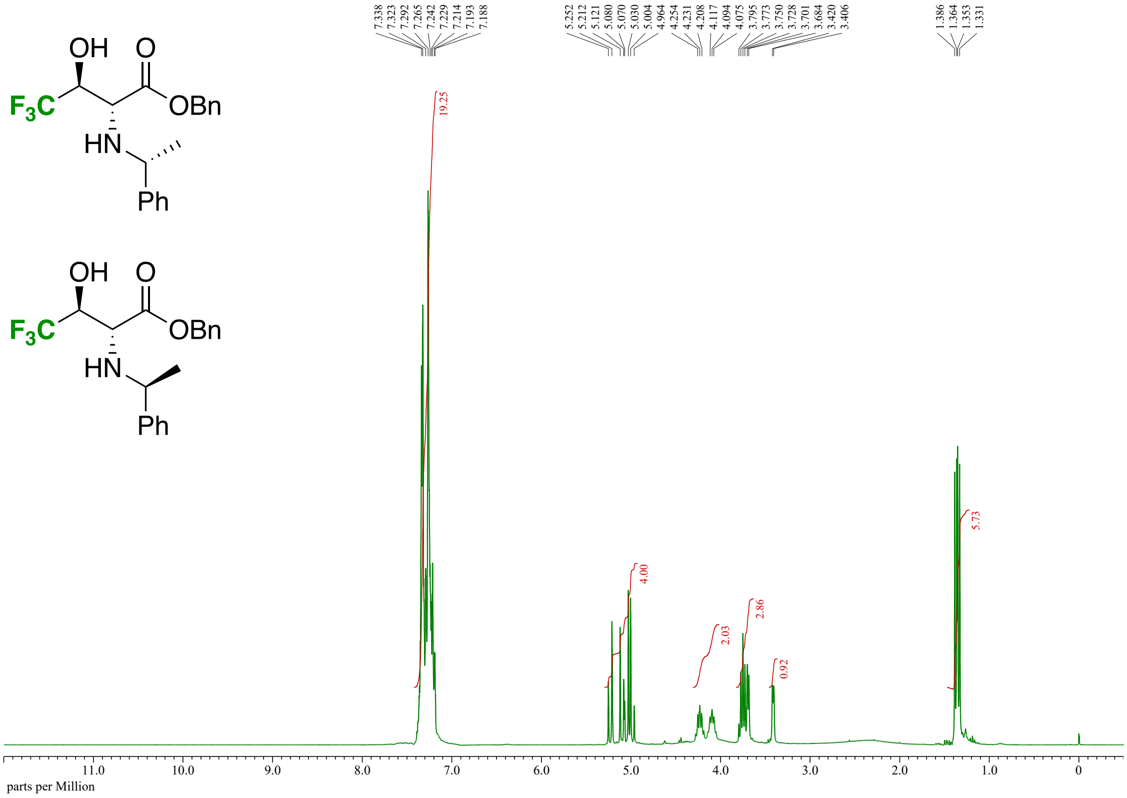

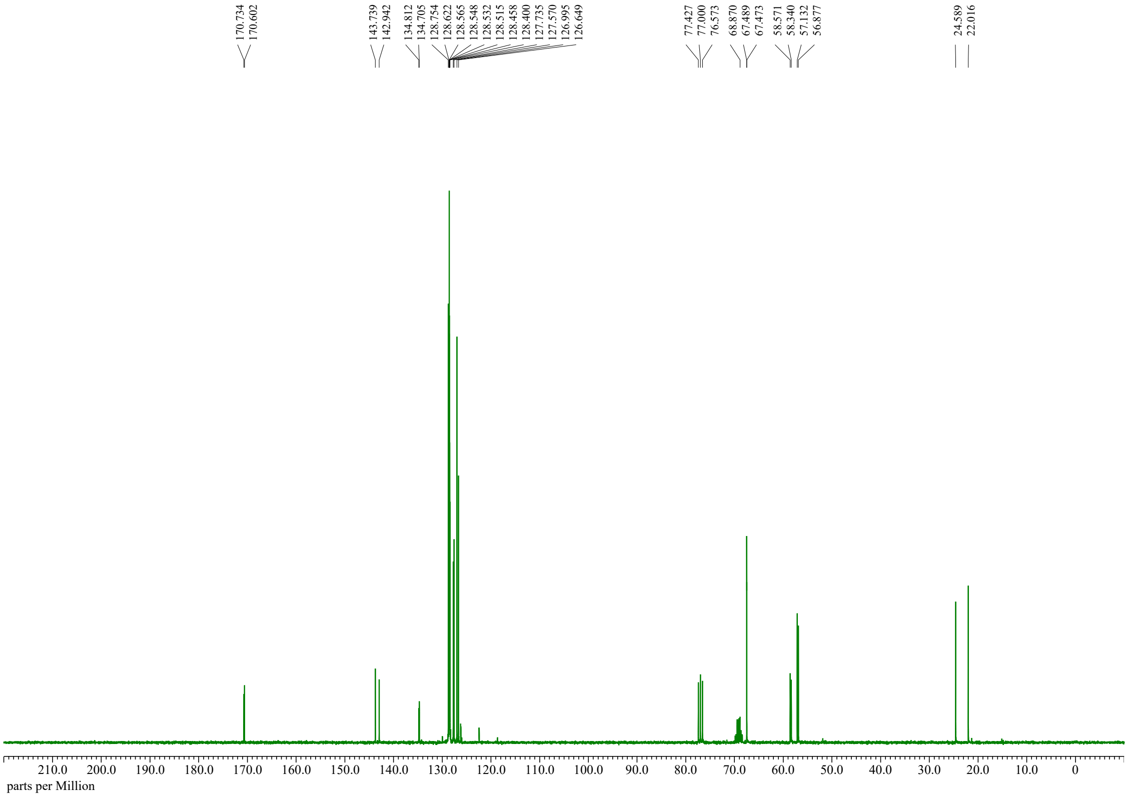

Benzyl (2*R**,3*S**)-2,3-*anti*-2-[(1*R**)-1-phenylethylamino]-4,4,4-trifluoro-3-hydroxybutanoate (**(2*R**,3*S**,2'*R**)-3bd**)

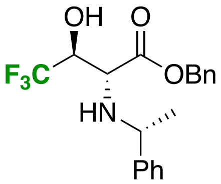

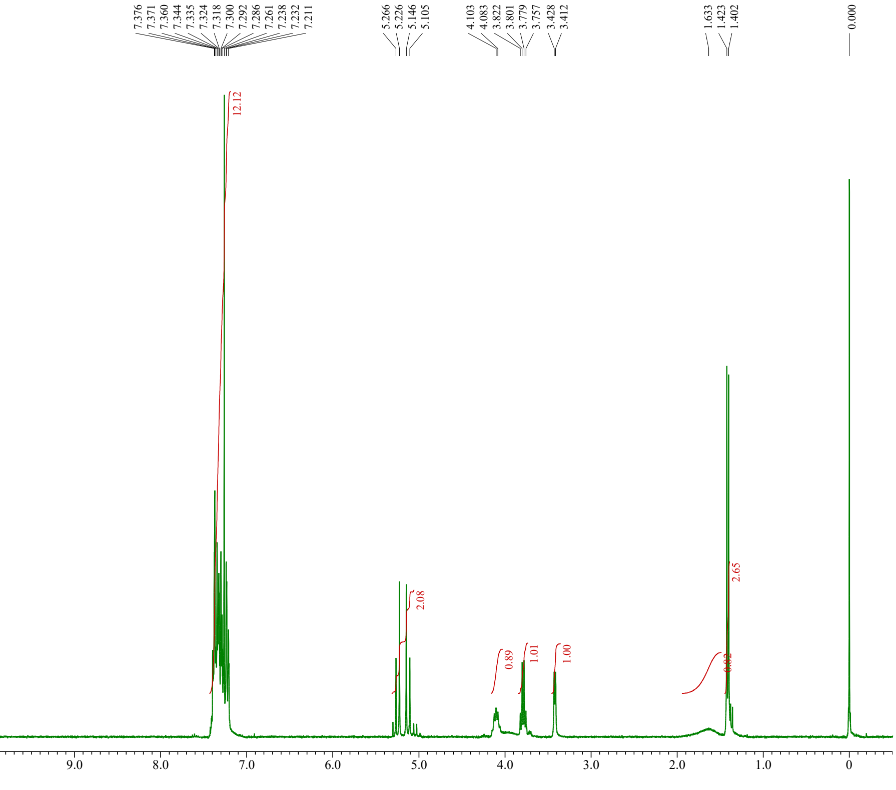

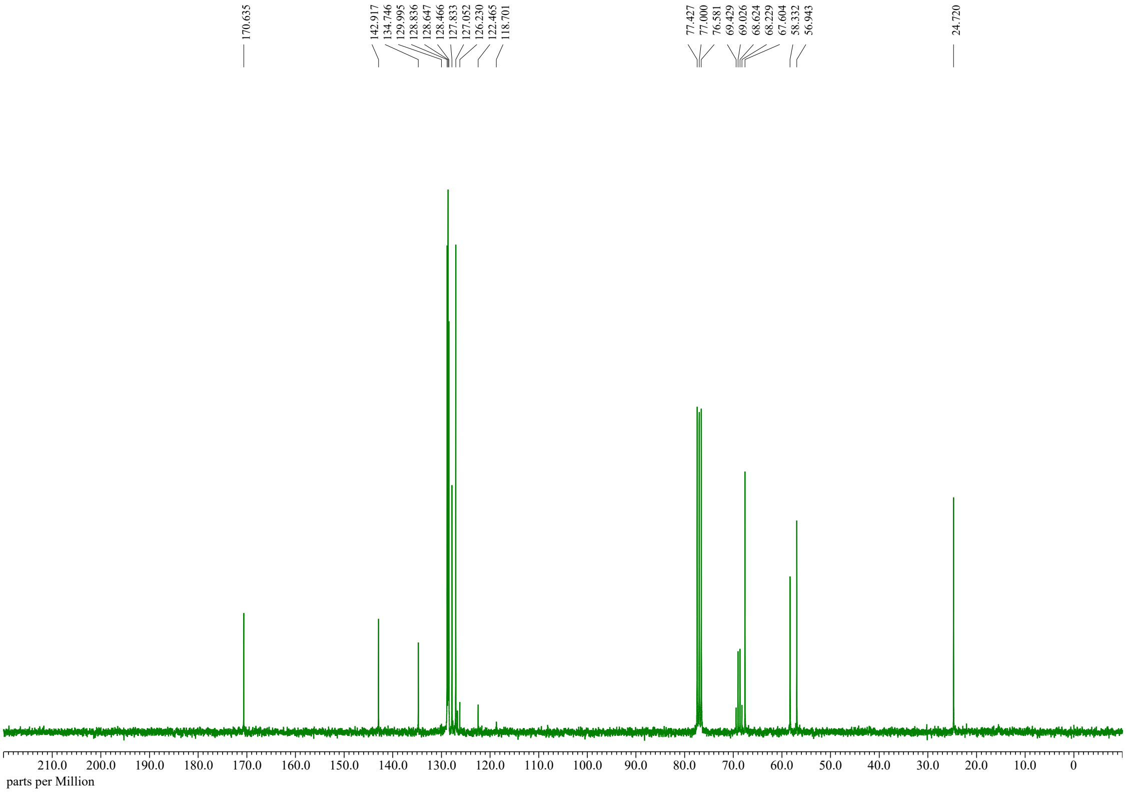

Benzyl 2,3-*anti*-2-[{(*S*)-1-(benzyloxycarbonyl)-2-methylbutyl}amino]-4,4,4-trifluoro-3-hydroxybutanoate (**3be**)

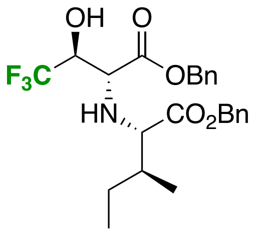

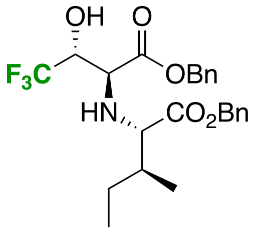

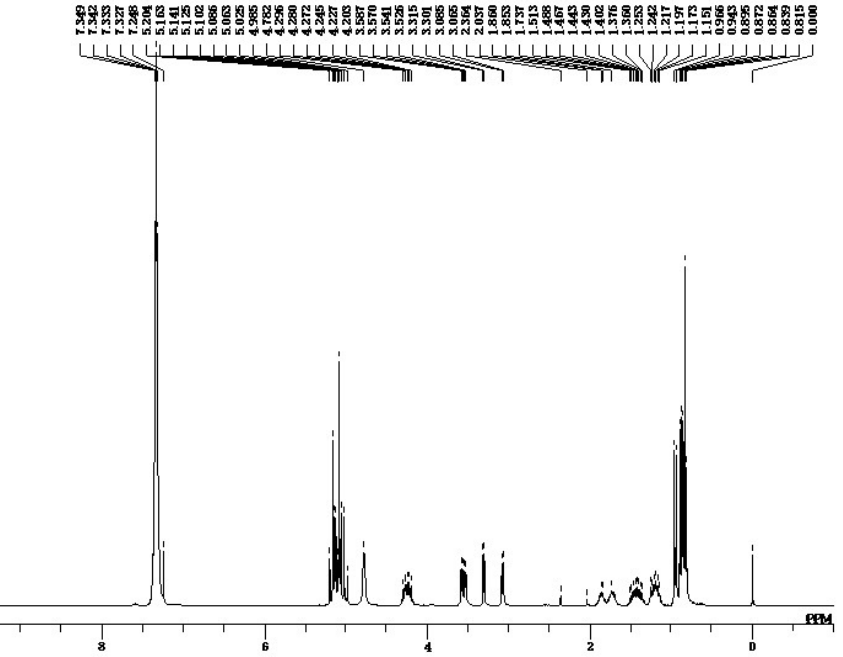

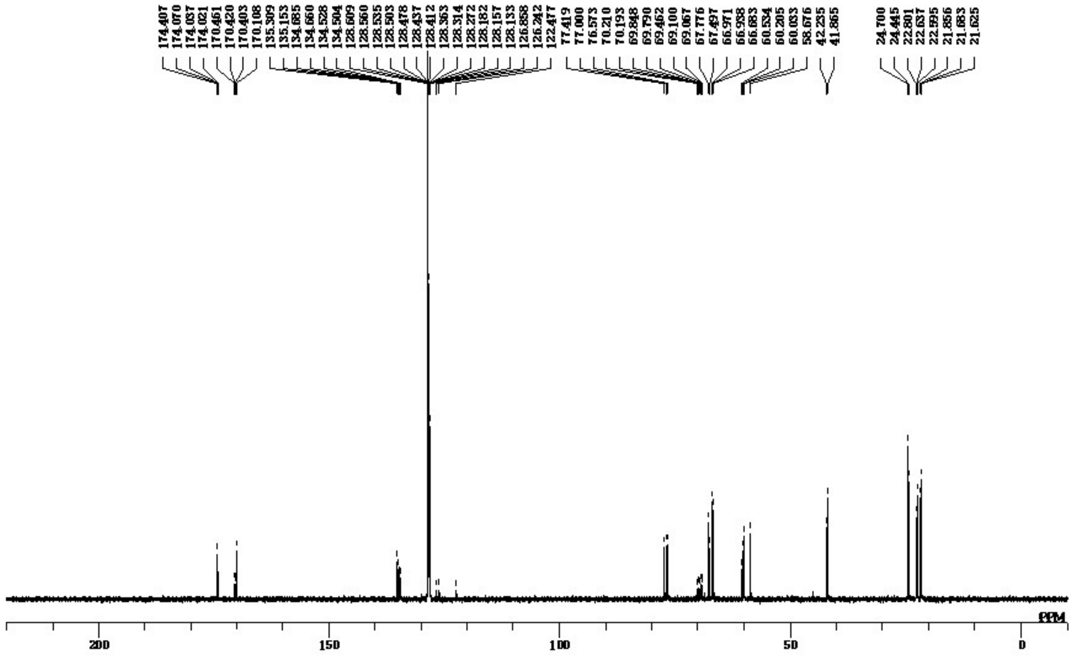

Benzyl 2,3-*anti*-2-(*N*,*N*-diethylamino)-4,4,4-trifluoro-3-hydroxybutanoate (**3bf**)

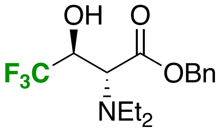

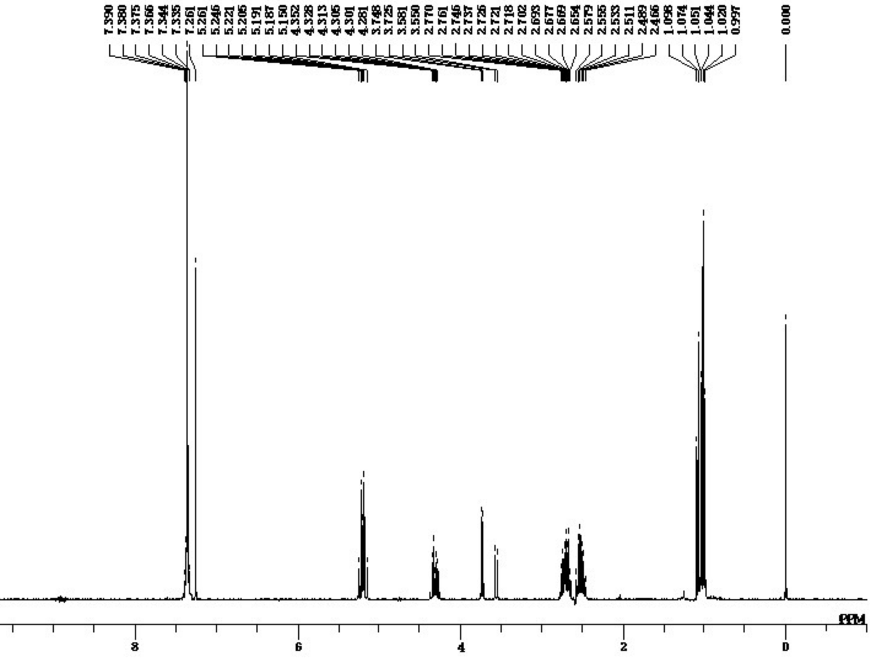

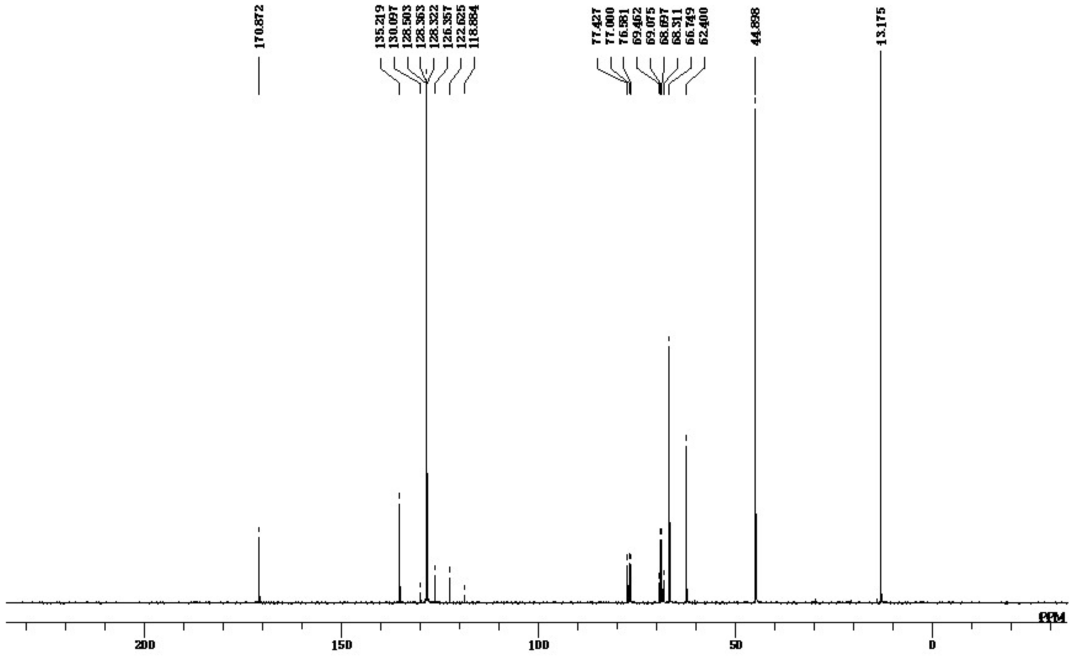

Benzyl 2,3-*anti*-2-(pyrrolidin-1-yl)-4,4,4-trifluoro-3-hydroxybutanoate (**3bg**)

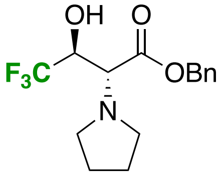

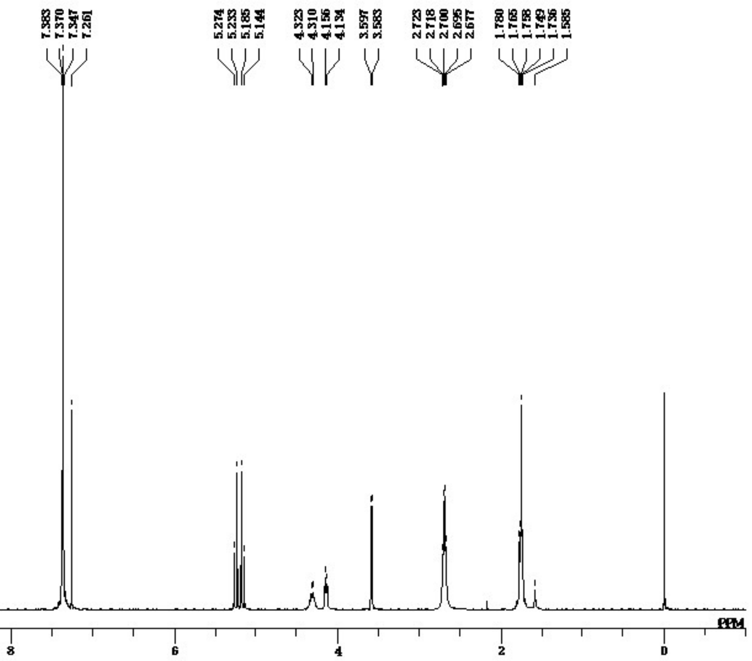

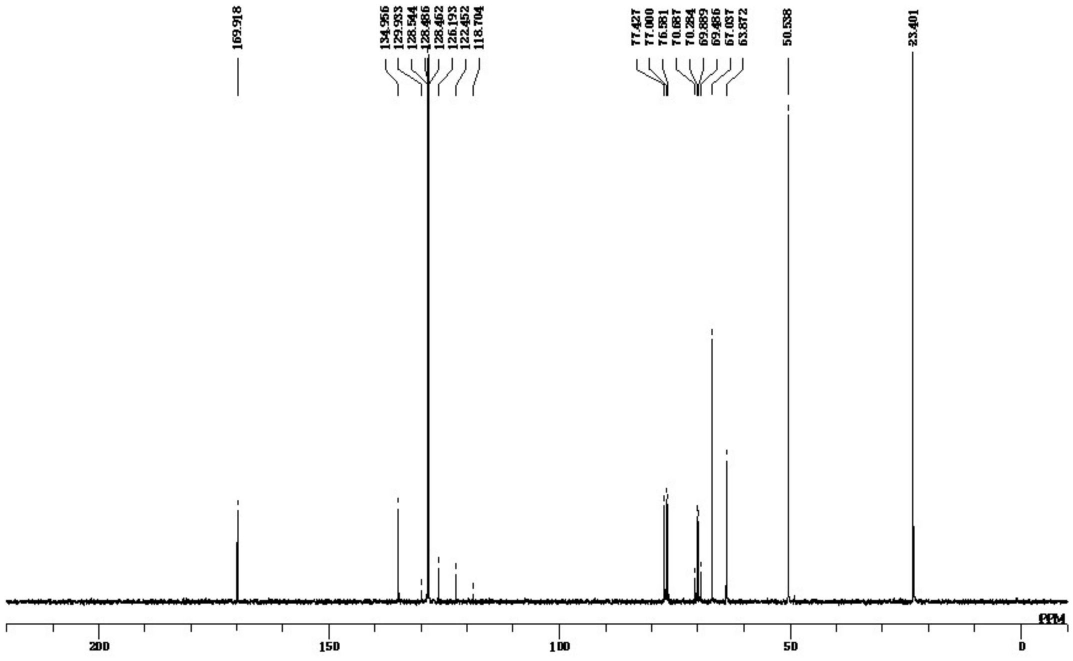

Benzyl 2,3-*anti*-4,4,4-trifluoro-3-hydroxy-2-(phenylmethylsulfenyl)butanoate (**4ba**)

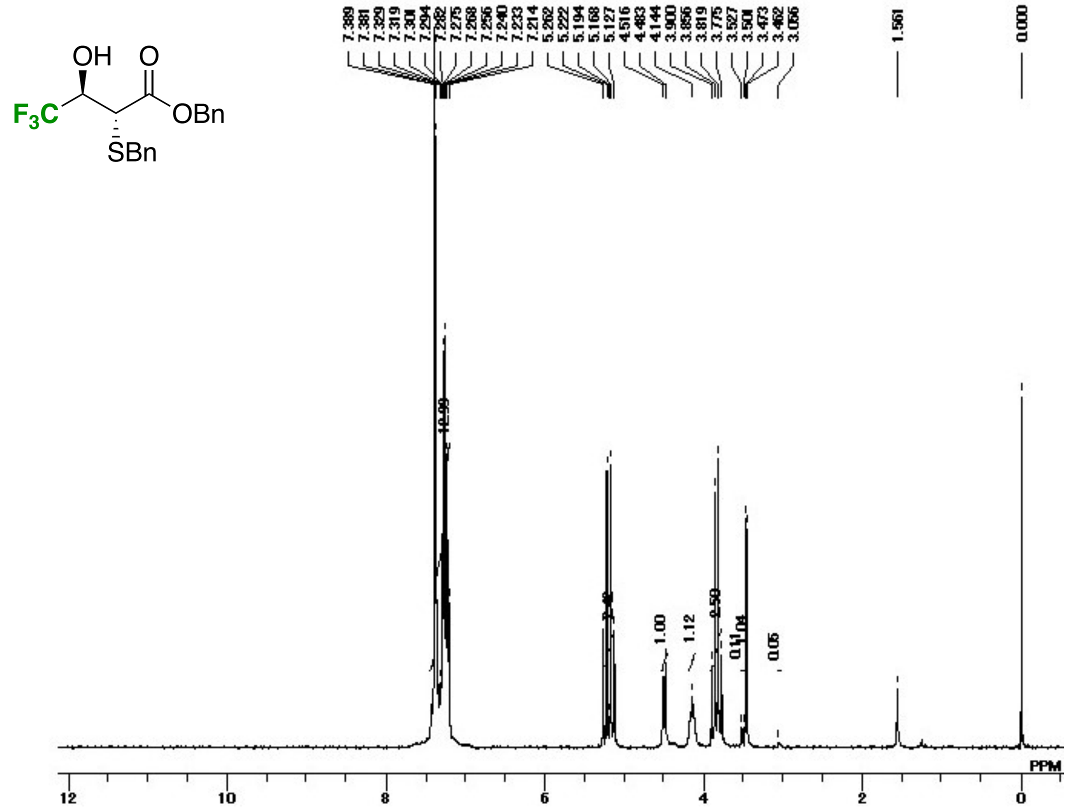

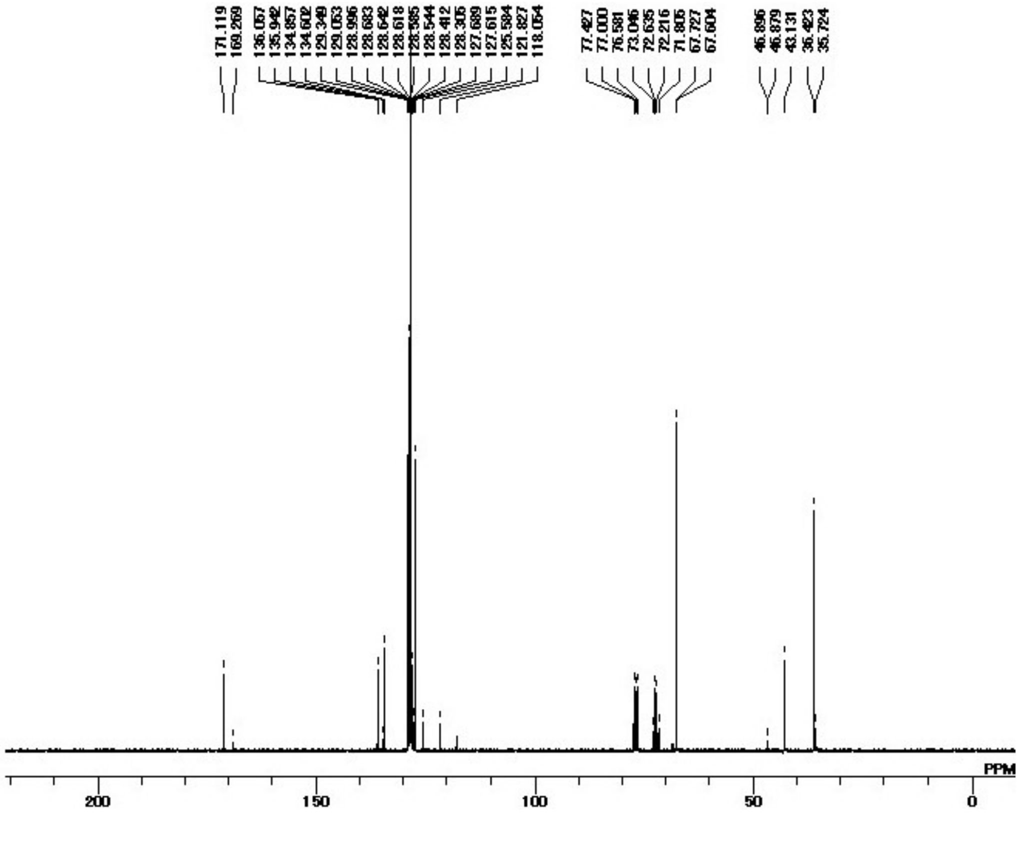

Benzyl 2,3-*anti*-4,4-difluoro-3-hydroxy-2-(phenylmethylsulfenyl)butanoate (**4ca**)

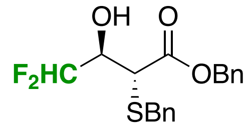

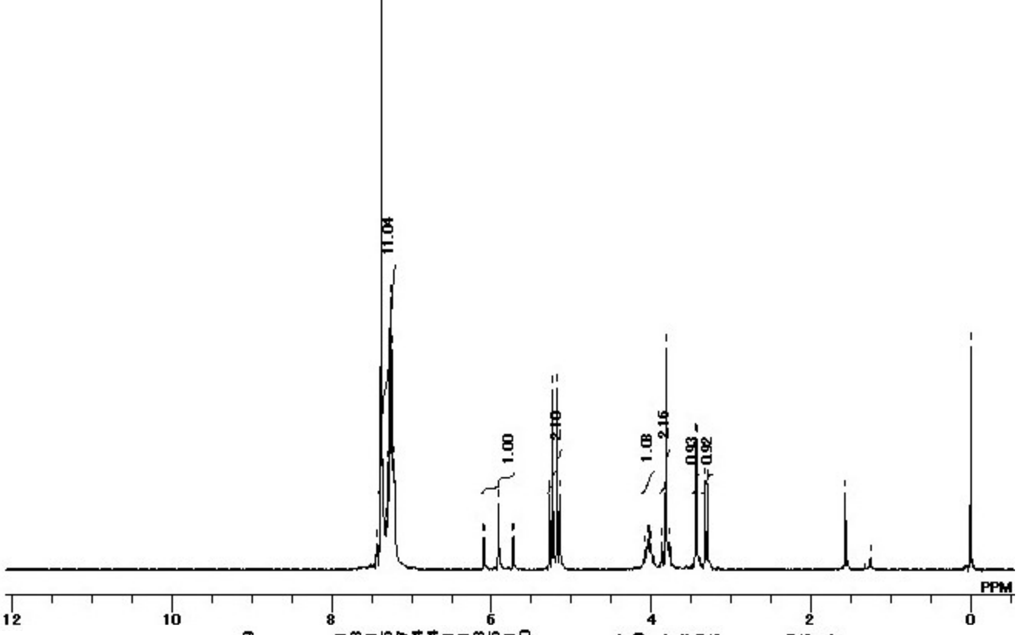

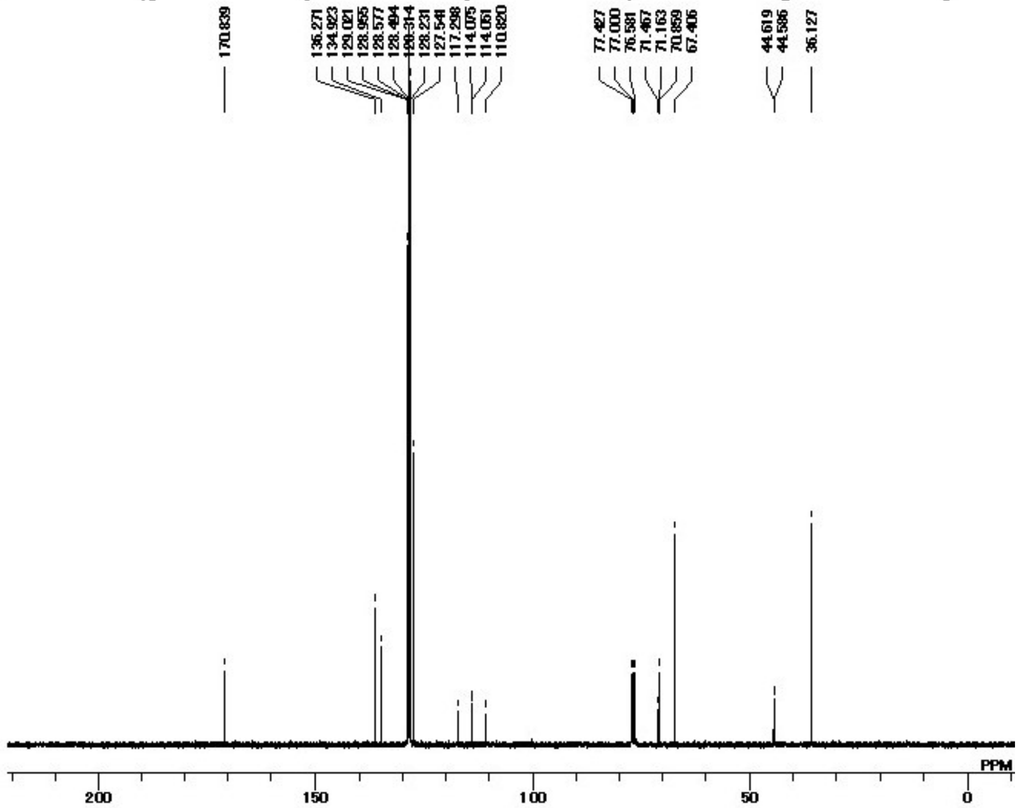

Benzyl 2,3-*anti*-4-chloro-4,4-difluoro-3-hydroxy-2-(phenylmethylsulfenyl)butanoate (**4da**)

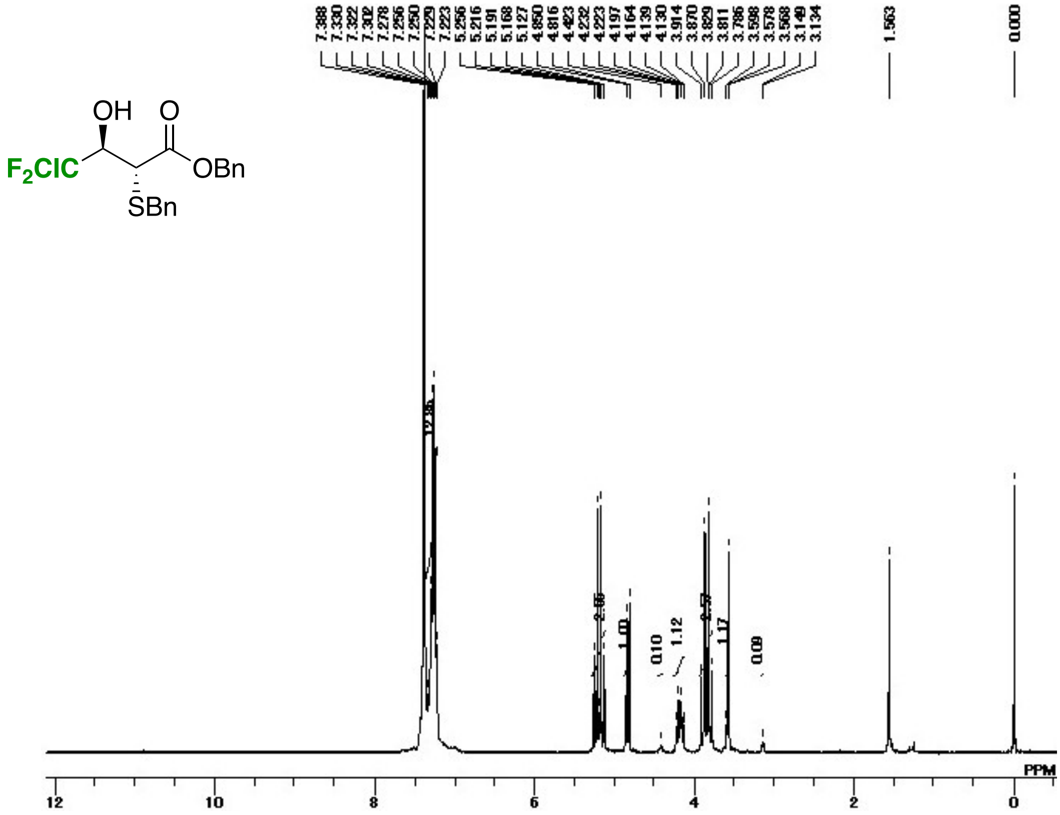

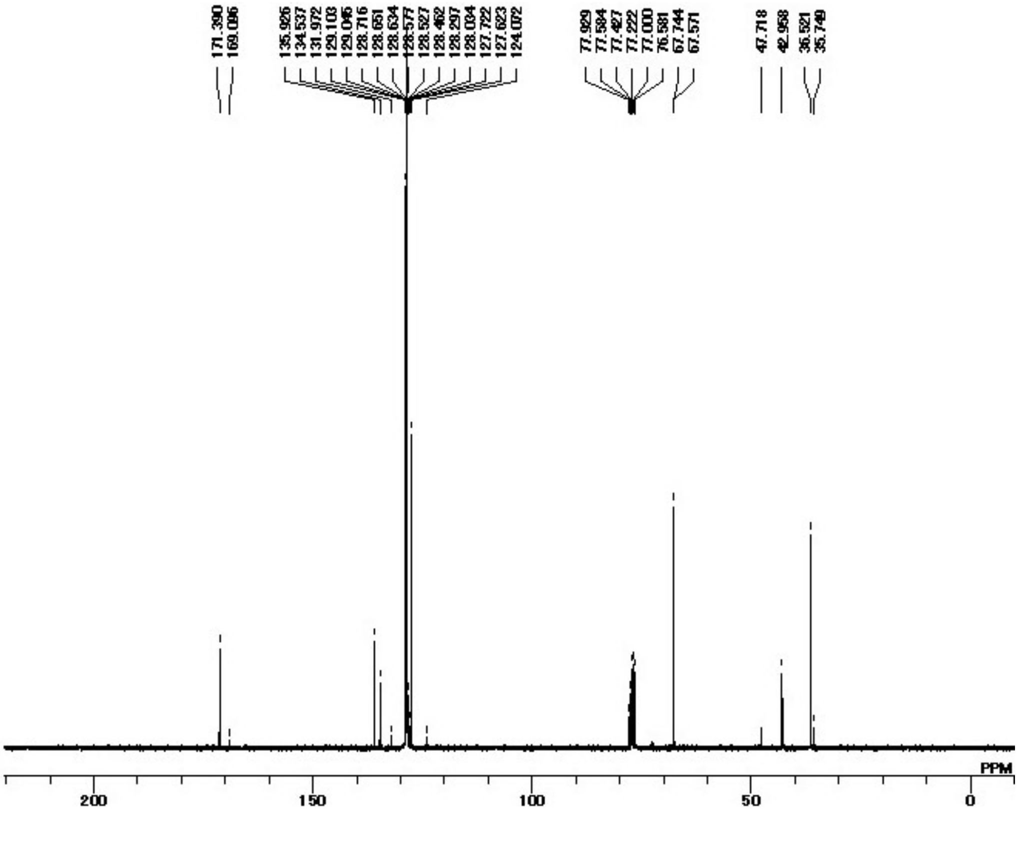

Benzyl 2,3-*anti*-4,4,5,5,5-pentafluoro-3-hydroxy-2-(phenylmethylsulfenyl)butanoate (**4ea**)

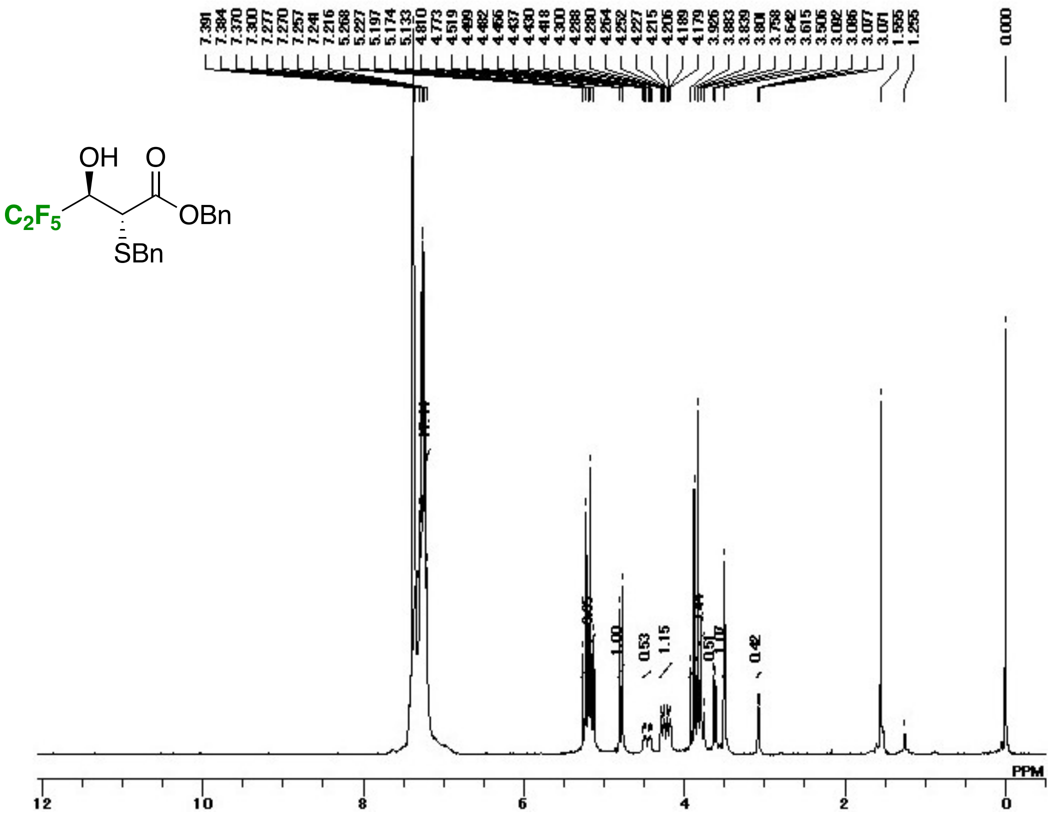

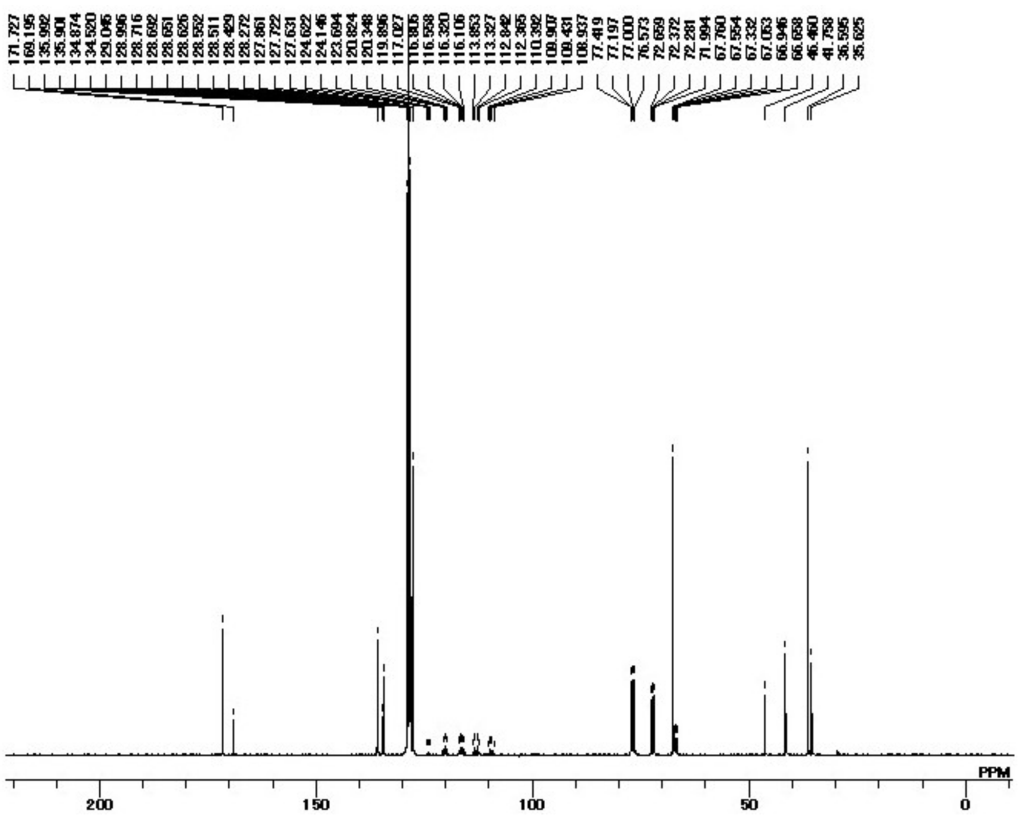

Benzyl 2,3-*anti*-2-(*n*-decylsulfenyl)-4,4,4-trifluoro-3-hydroxybutanoate (**4bb**)

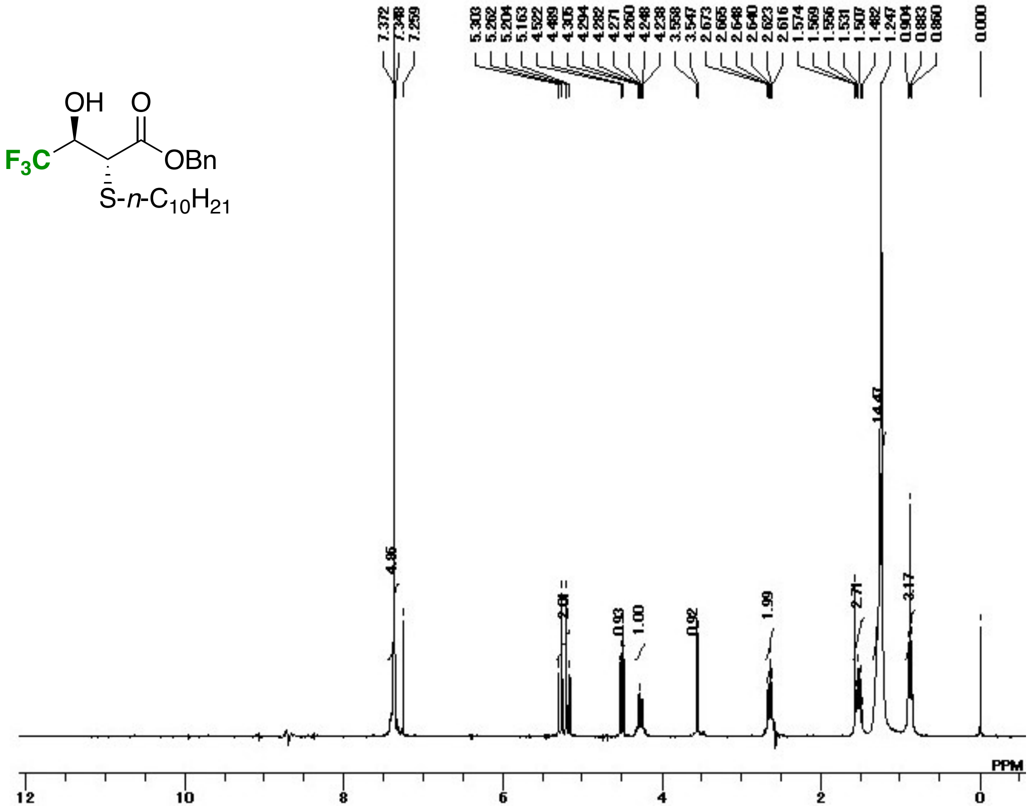

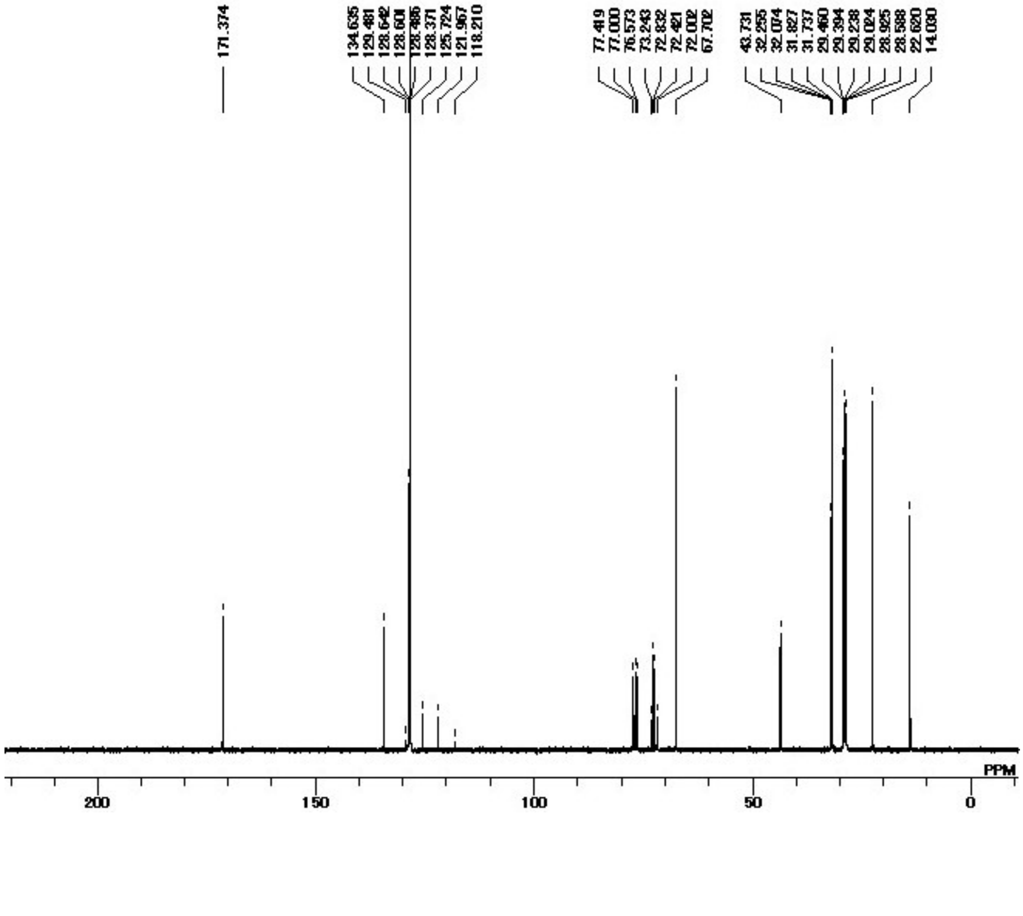

Benzyl 2,3-*anti*-4,4,4-trifluoro-3-hydroxy-2-(phenylsulfenyl)butanoate (**4bc**)

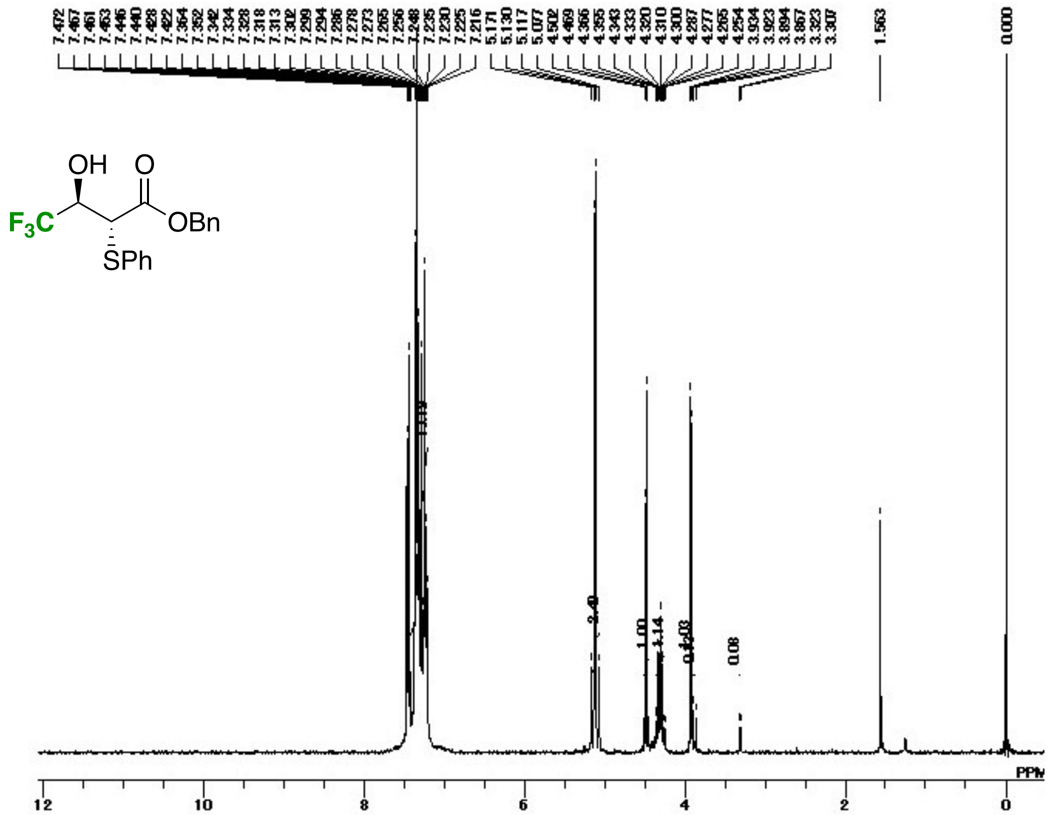

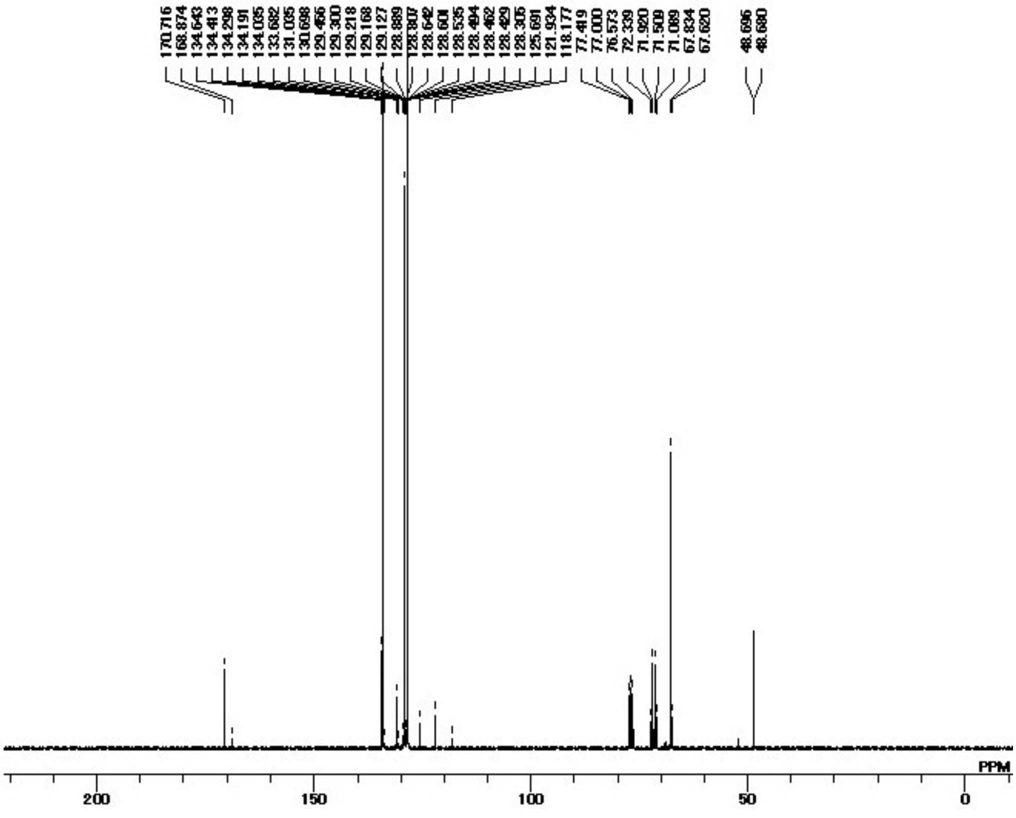

Benzyl 2,3-*anti*-4,4,4-trifluoro-3-hydroxy-2-{(methoxycarbonyl)methylsulfenyl)}-butanoate (**4bd**)

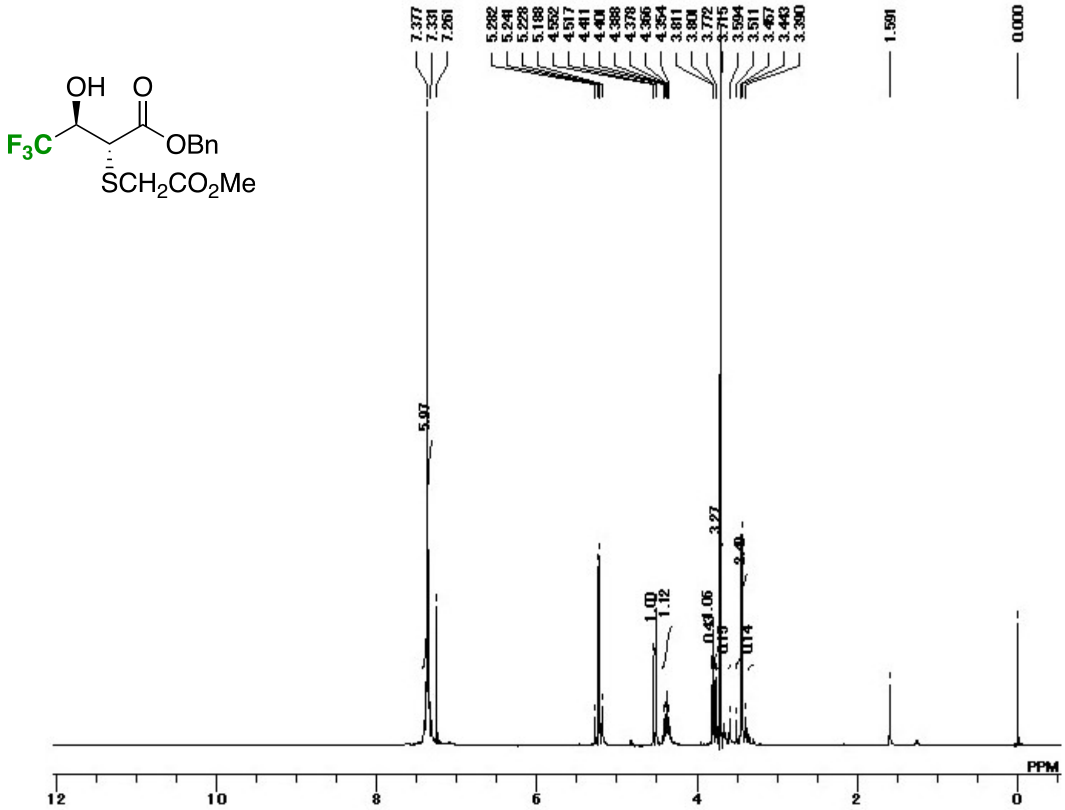

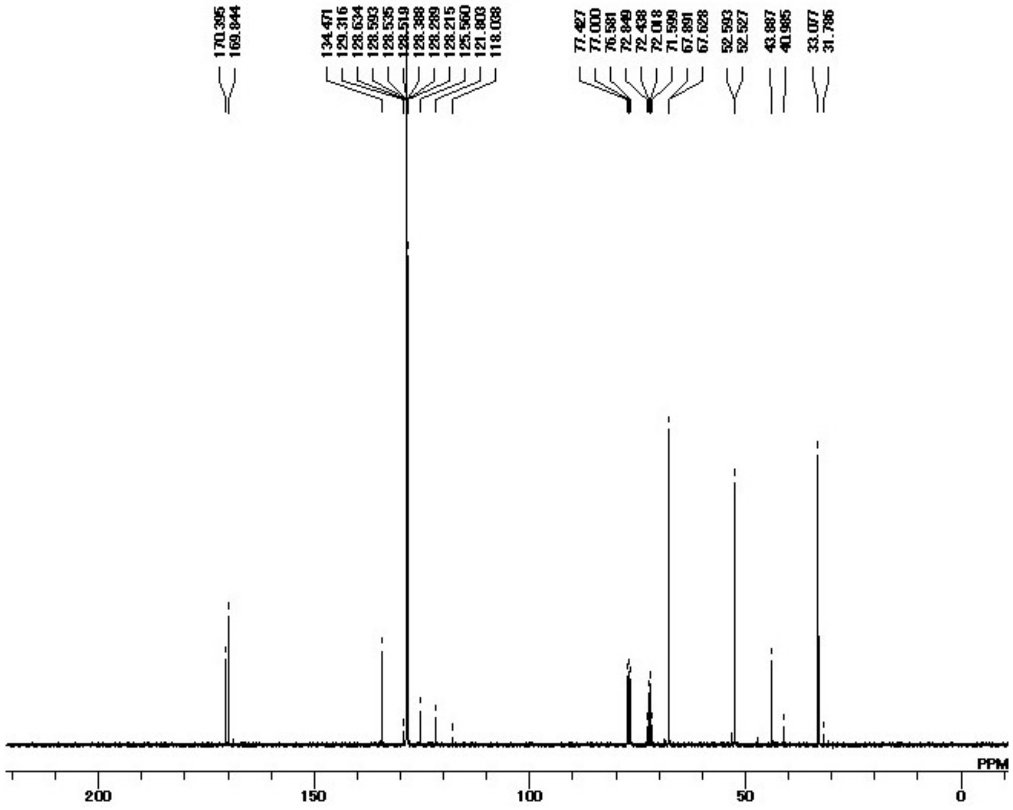

Benzyl 2,3-*anti*-2-chloro-4,4,4-trifluoro-3-hydroxybutanoate (**5ba**)

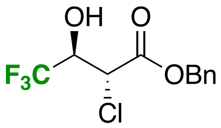

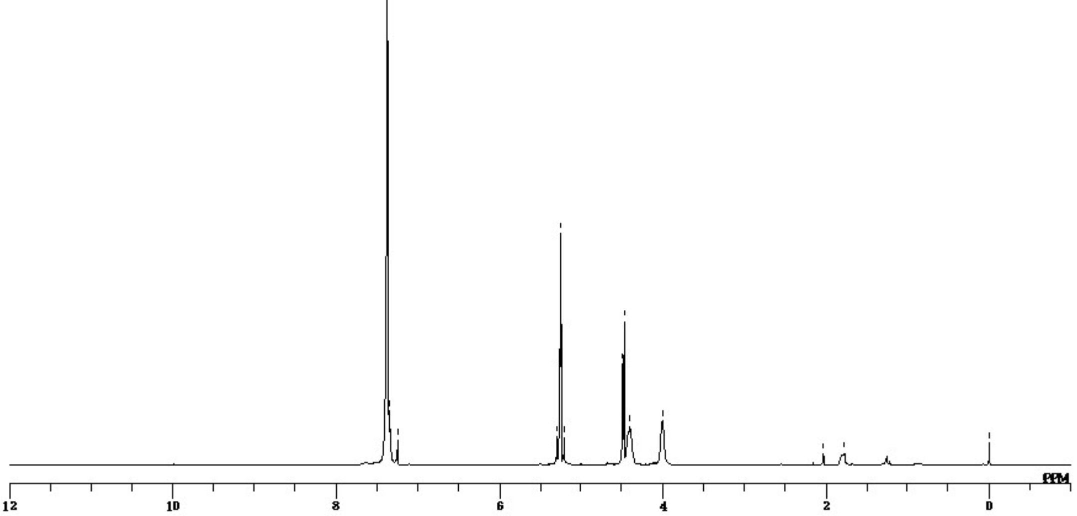

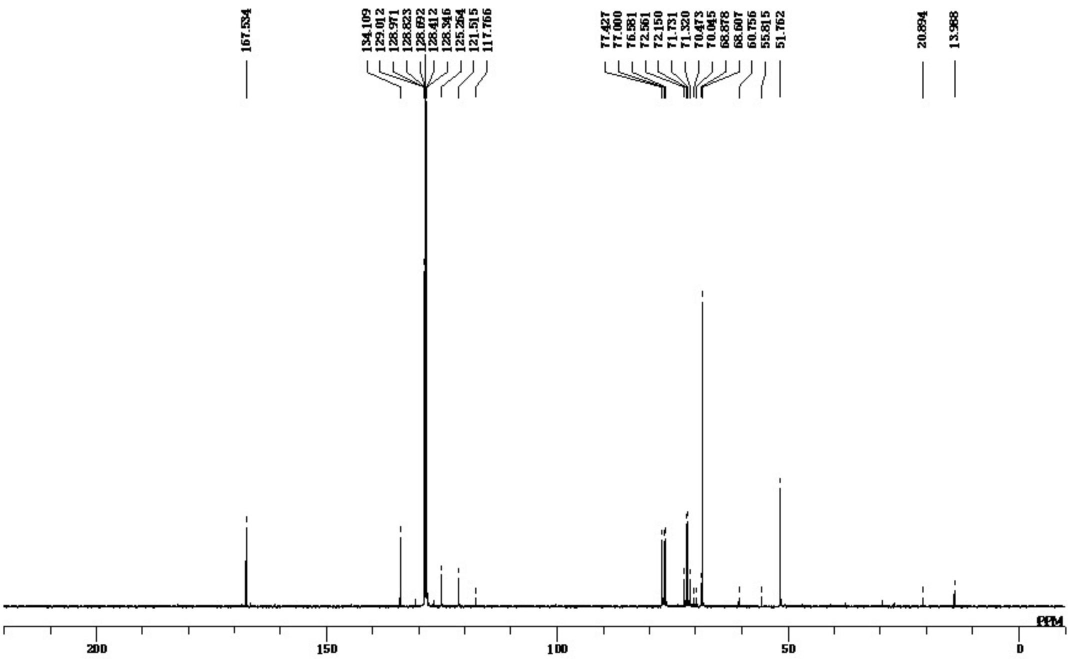

Benzyl 2,3-*anti*-2-bromo-4,4,4-trifluoro-3-hydroxybutanoate (**5bb**)

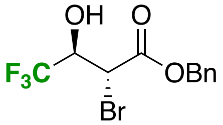

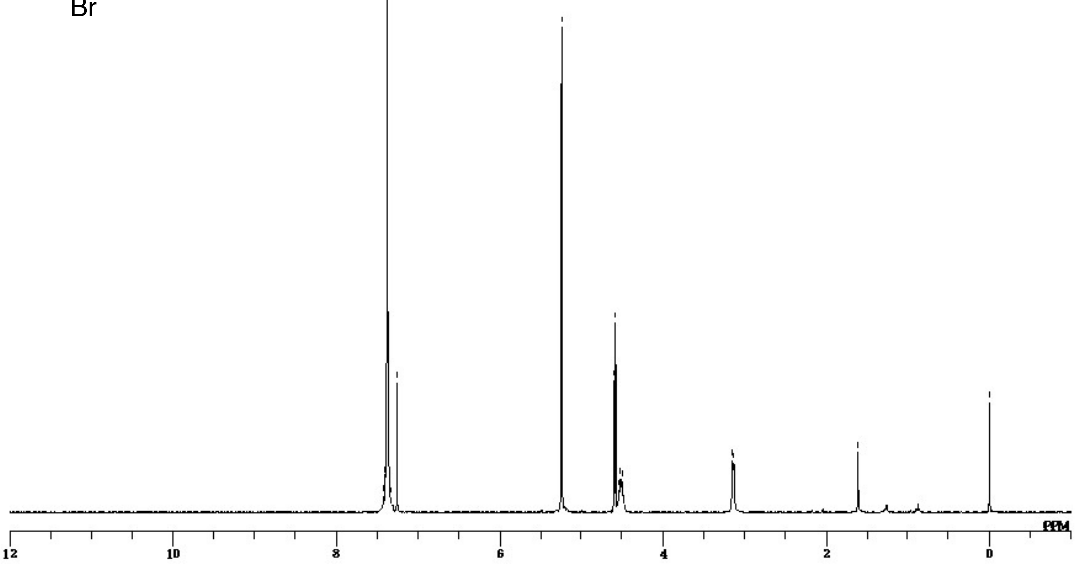

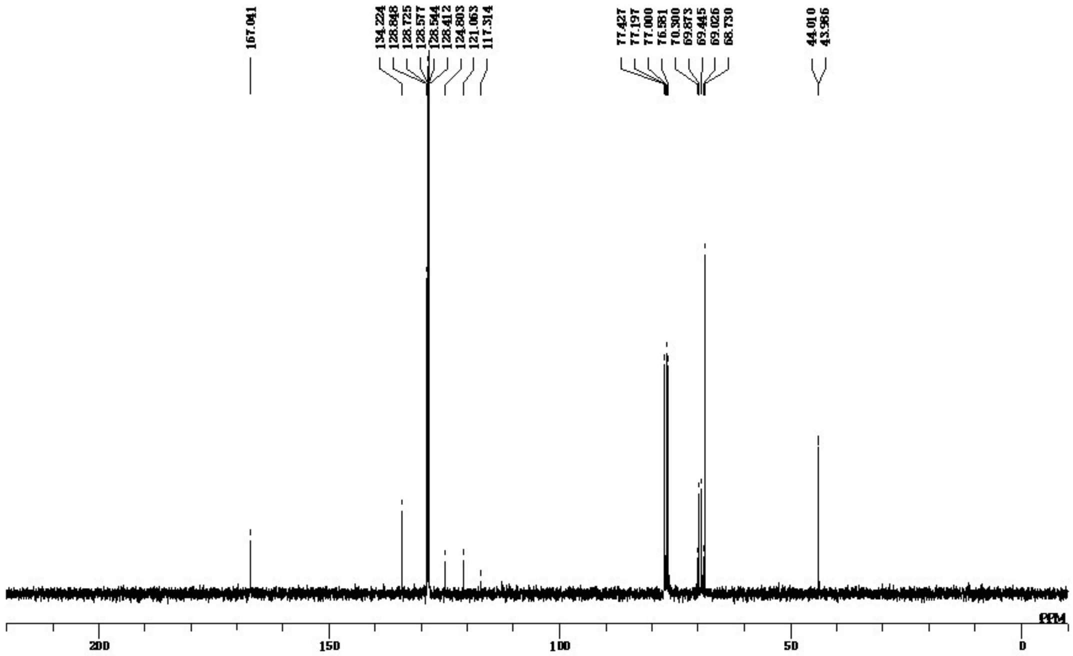

Benzyl 2,3-*anti*-4,4,4-trifluoro-3-hydroxy-2-iodobutanoate (**5bc**)

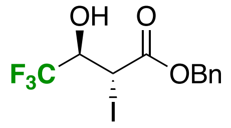

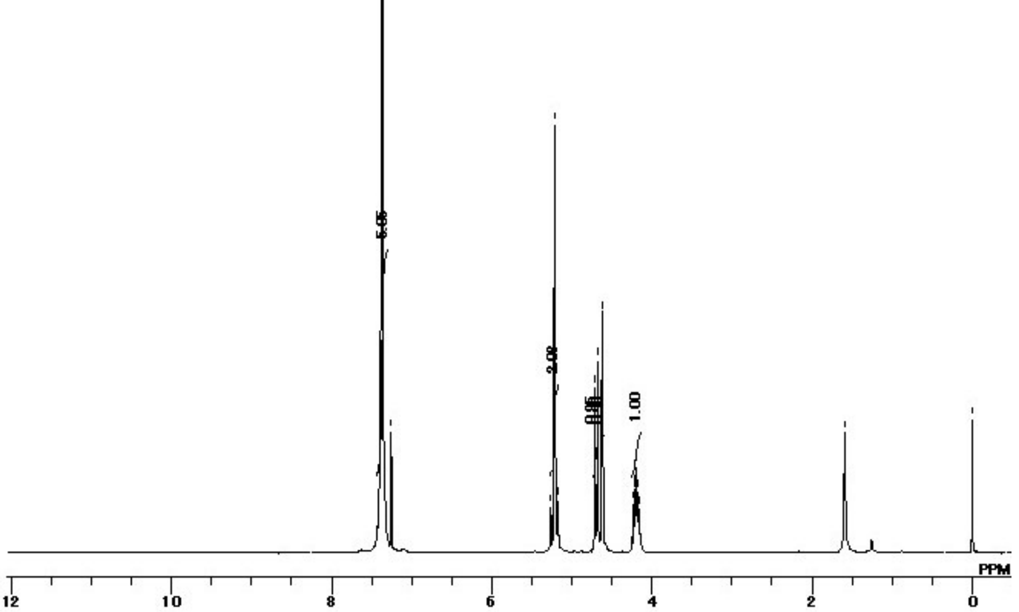

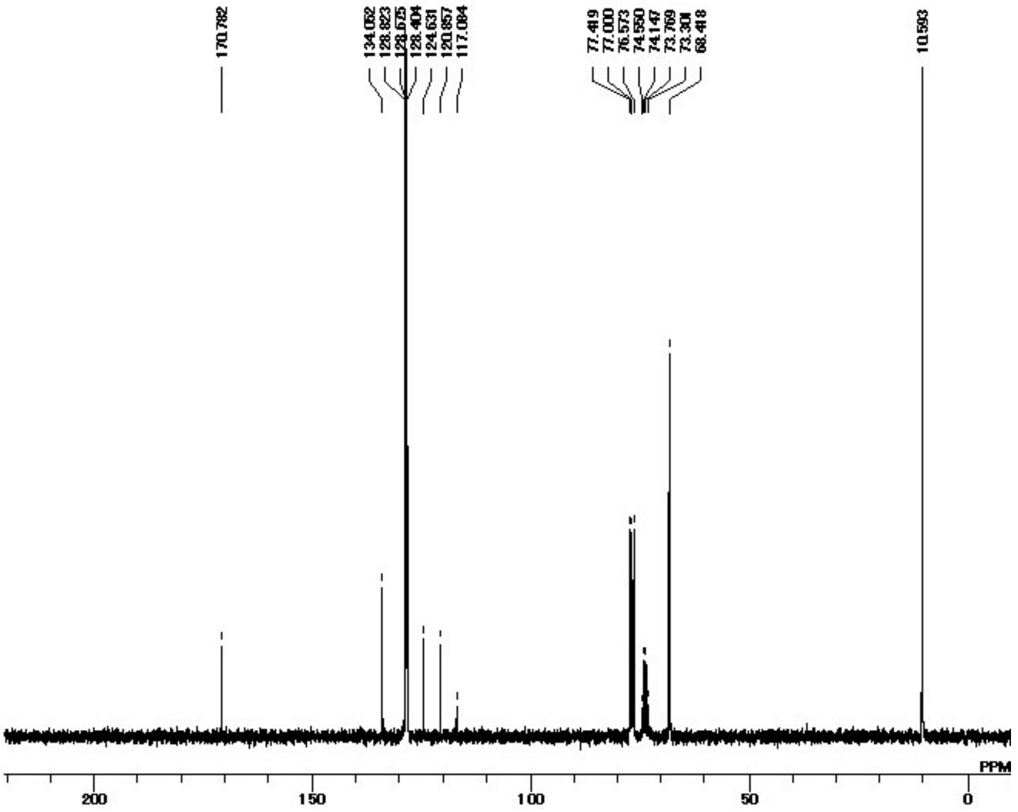

4-Benzyl 3-ethyl *anti*,*syn*-tetrahydro-2-oxo-4-(trifluoromethyl)furandicarboxylate (***anti*,*syn*-7a**)

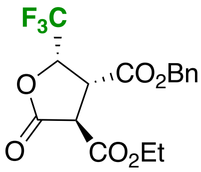

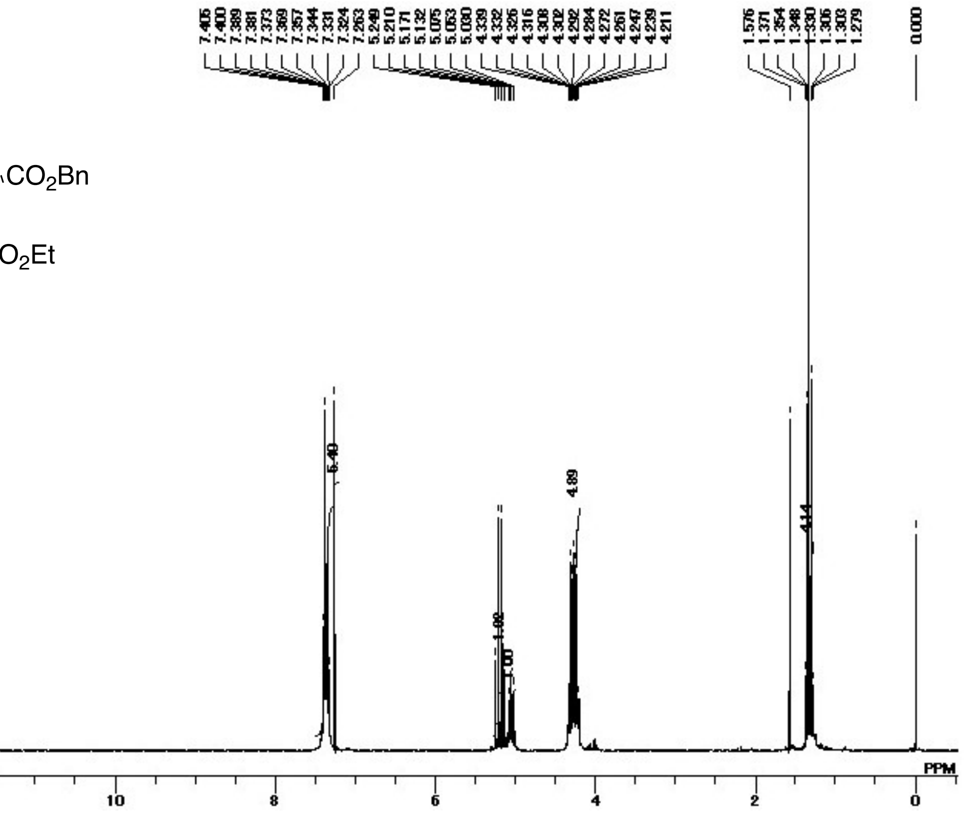

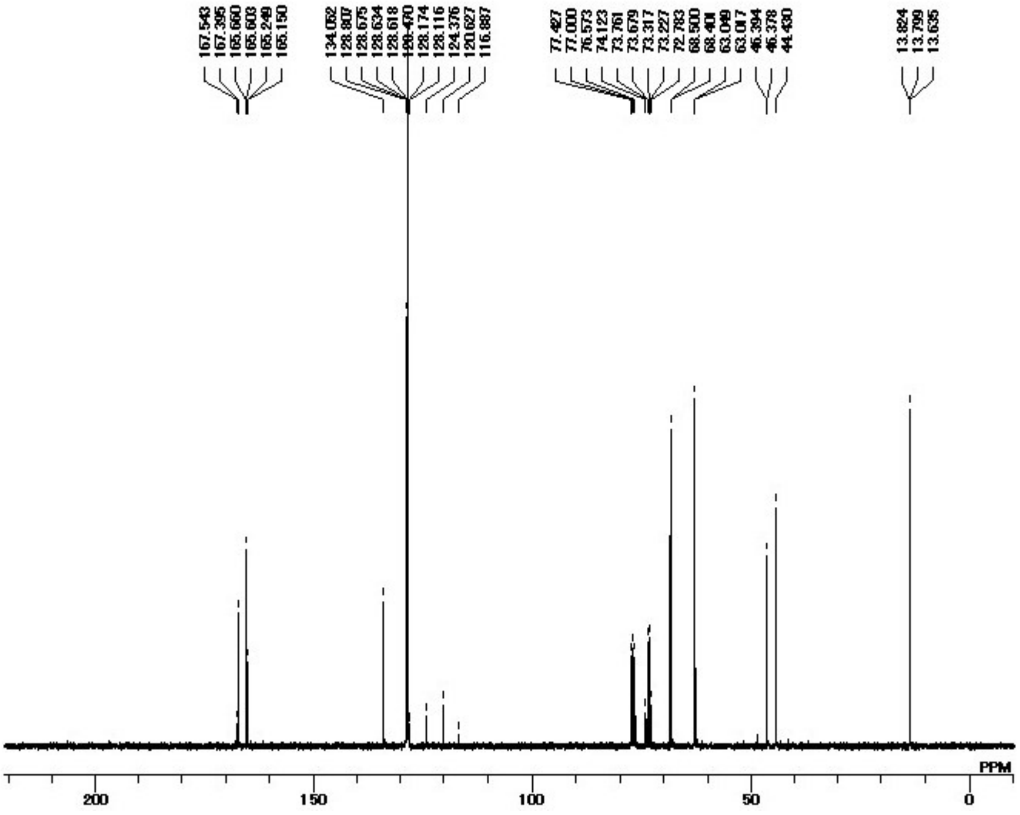

3,4-Diethyl *anti*,*syn*-tetrahydro-2-oxo-4-(trifluoromethyl)furandicarboxylate (***anti*,*syn*-7b**)

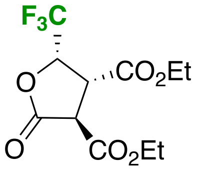

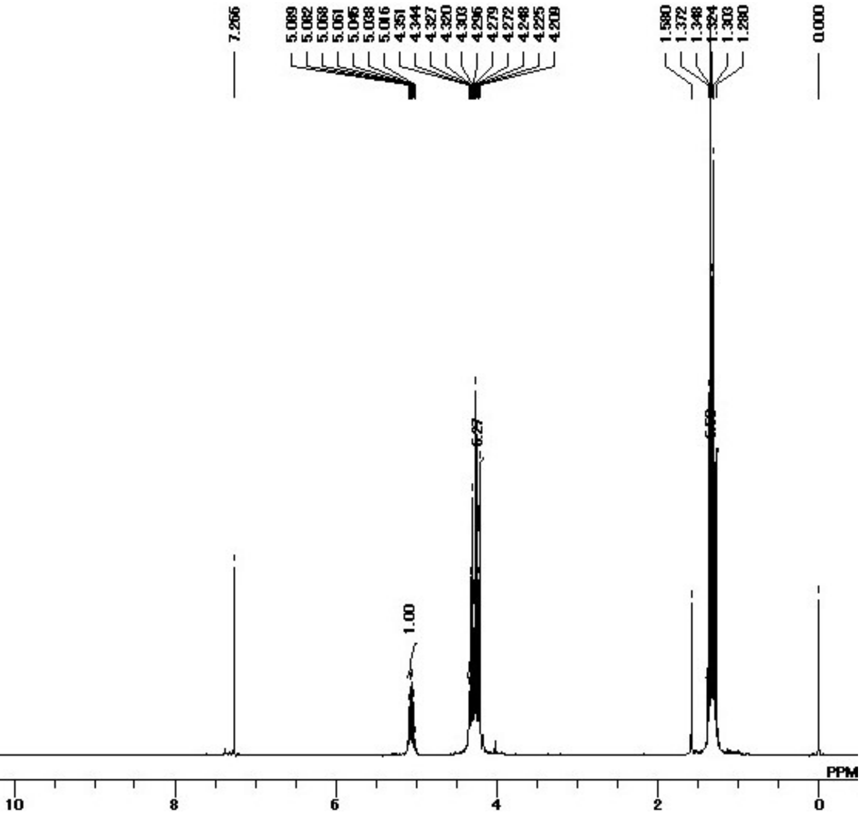

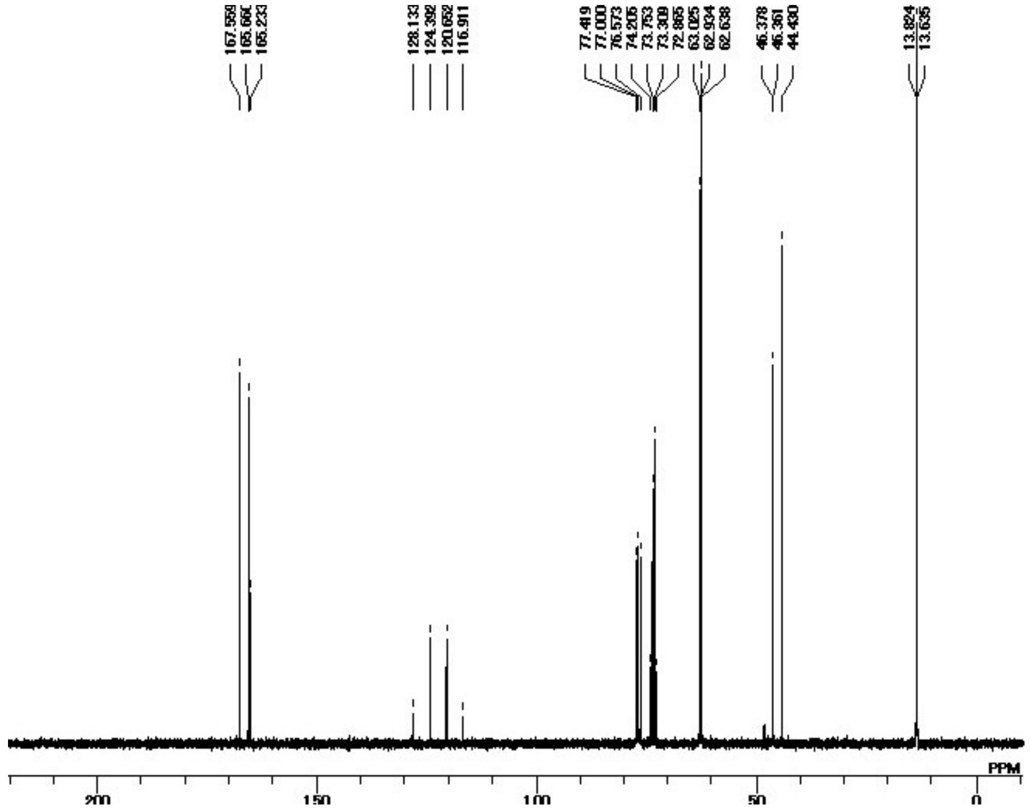

4-Benzyl 3-ethyl *syn*,*syn*-tetrahydro-2-oxo-4-(trifluoromethyl)furandicarboxylate (***syn*,*syn*-7a**)

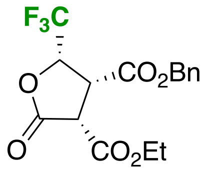

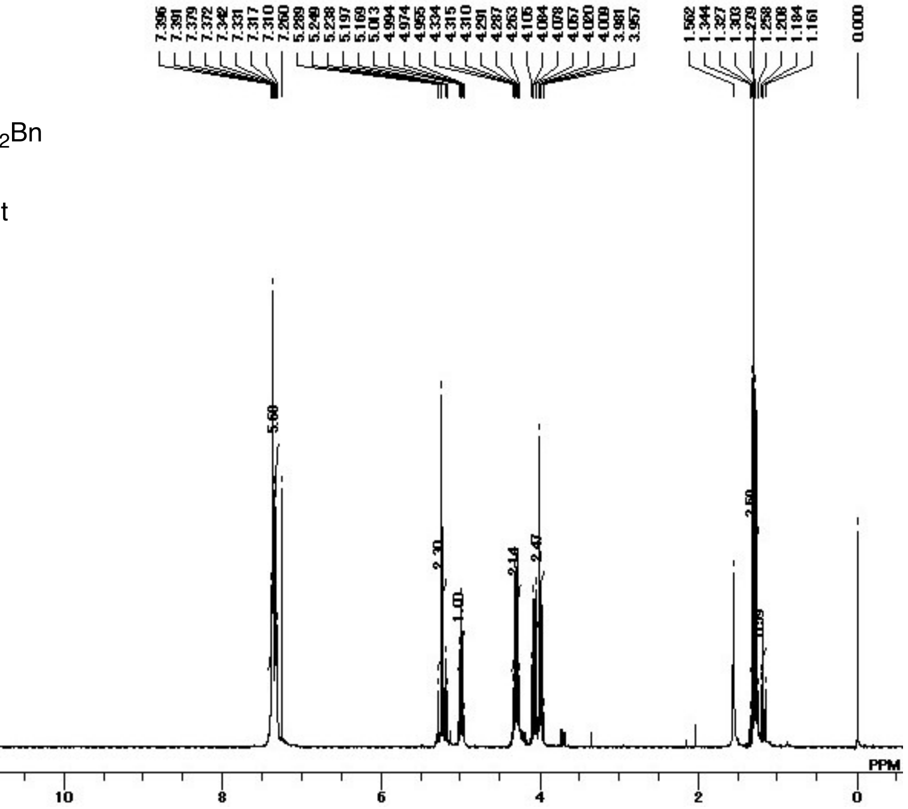

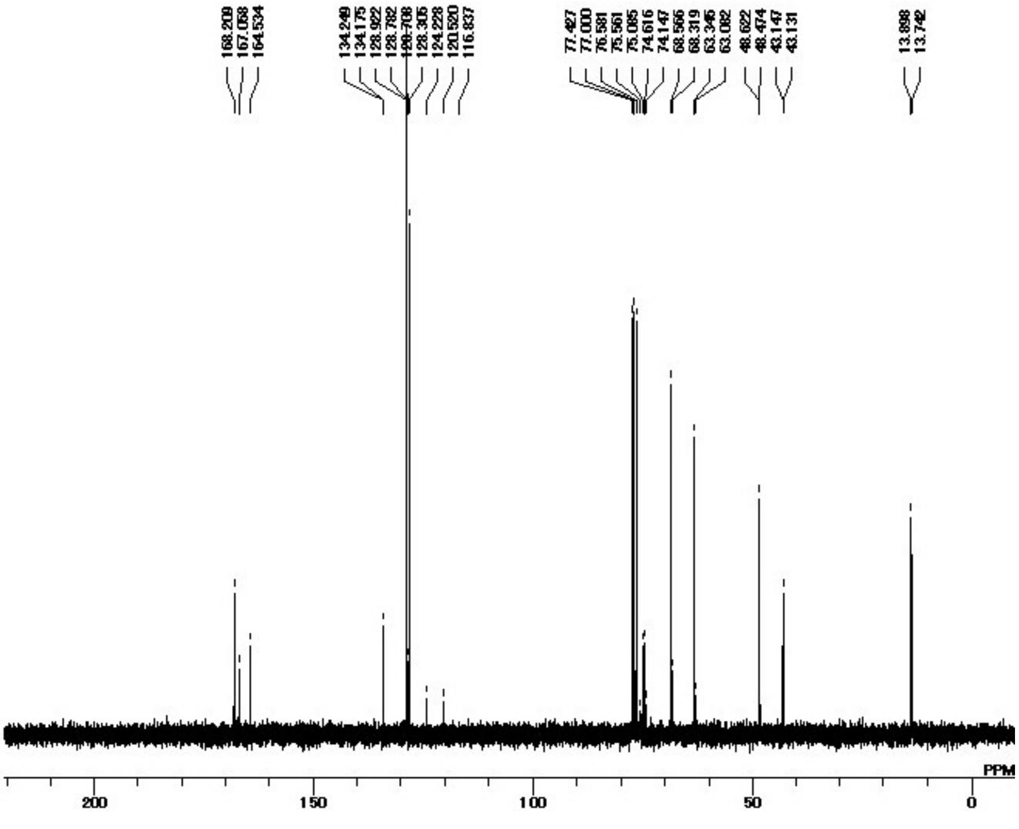

3,4-Dibenzyl *anti*,*syn*-tetrahydro-2-oxo-4-(trifluoromethyl)furandicarboxylate (***anti,syn*-7c**)

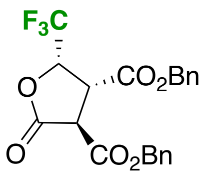

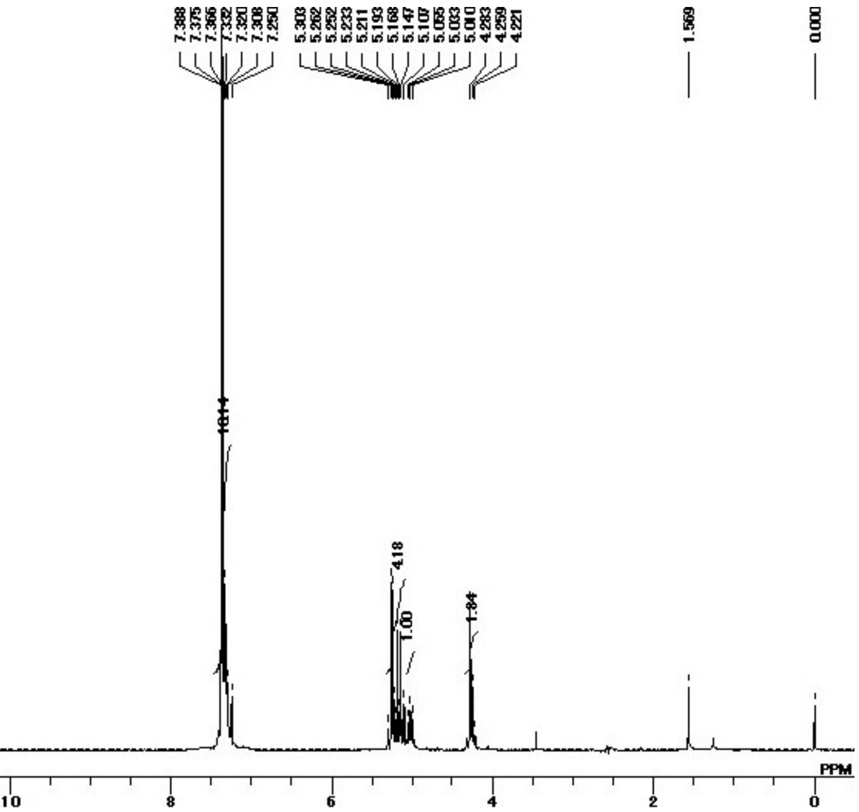

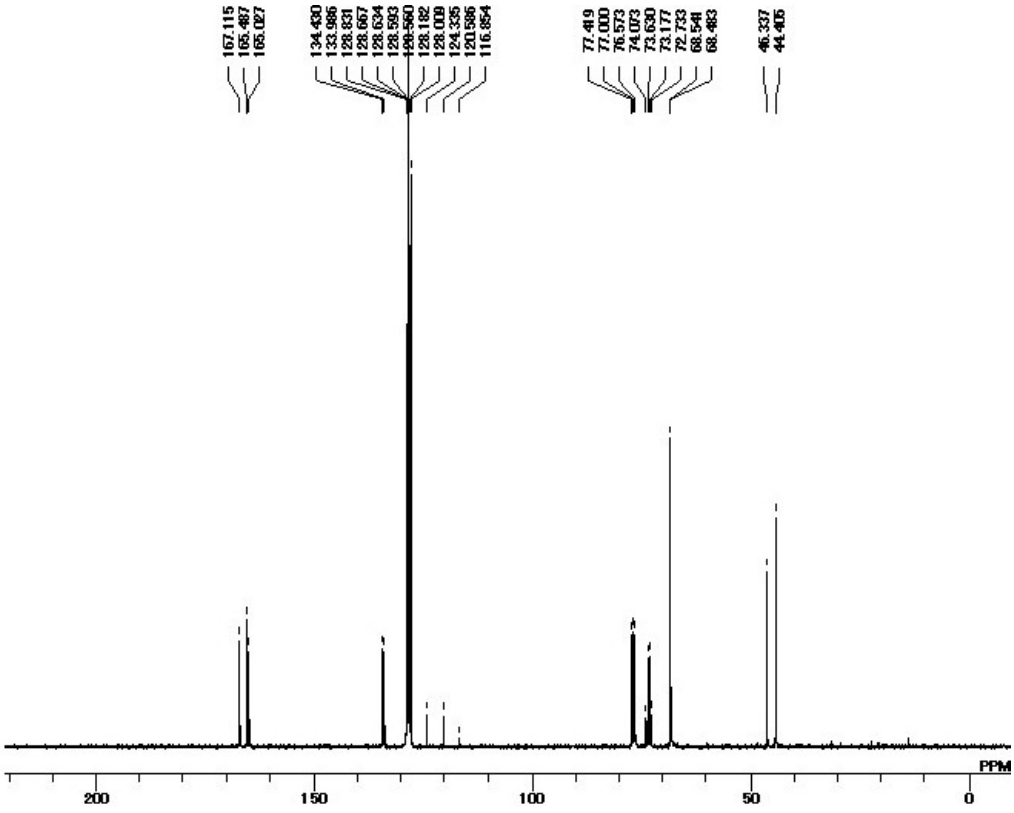

Benzyl *anti*-5-amino-4-cyano-2-(trifluoromethyl)-2,3-dihydrofuran-3-carboxylate (***syn*-7d**)

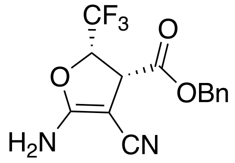

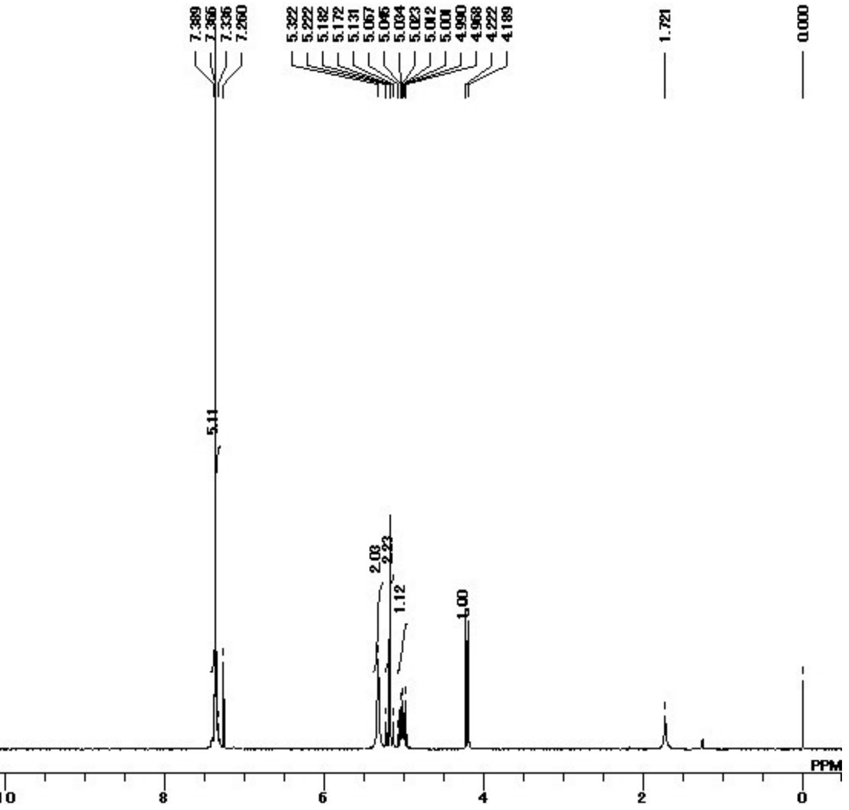

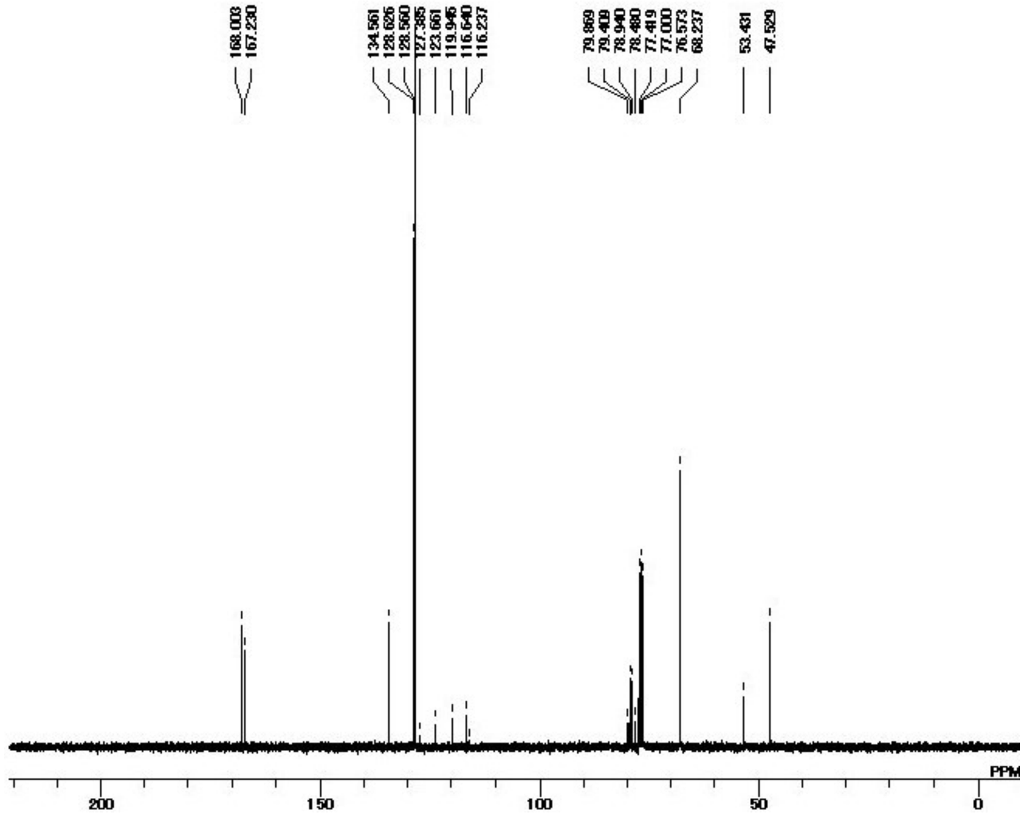

3-Benzyl-4-ethyl *anti*-5-amino-2-(trifluoromethyl)-2,3-dihydrofurandicarboxylate (***syn*-7e**)

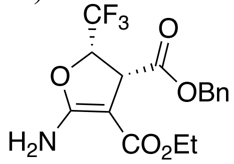

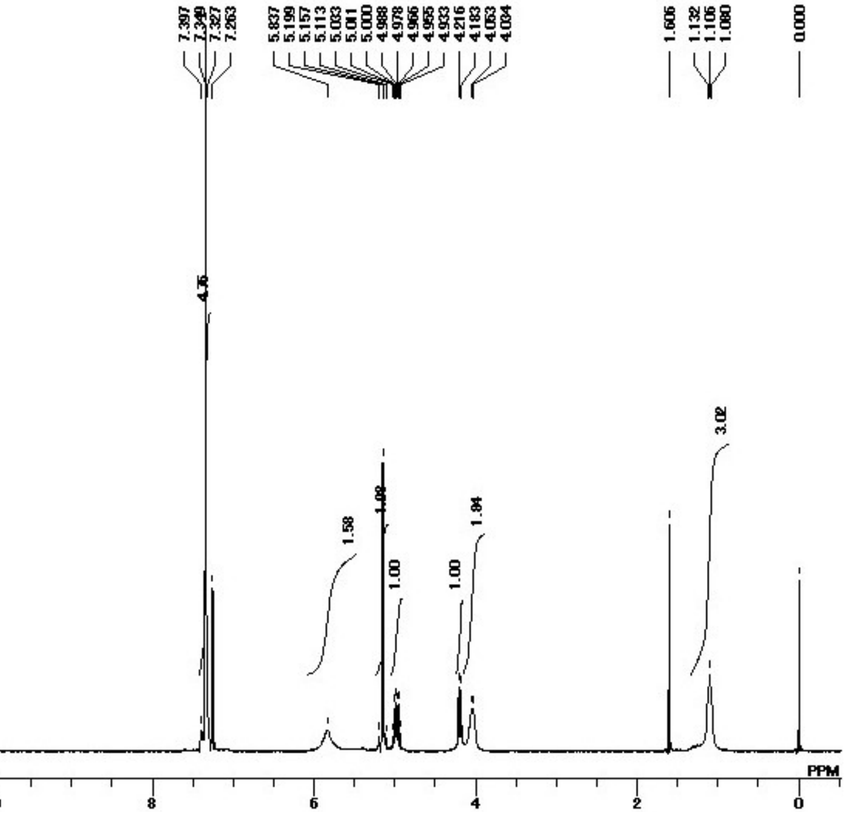

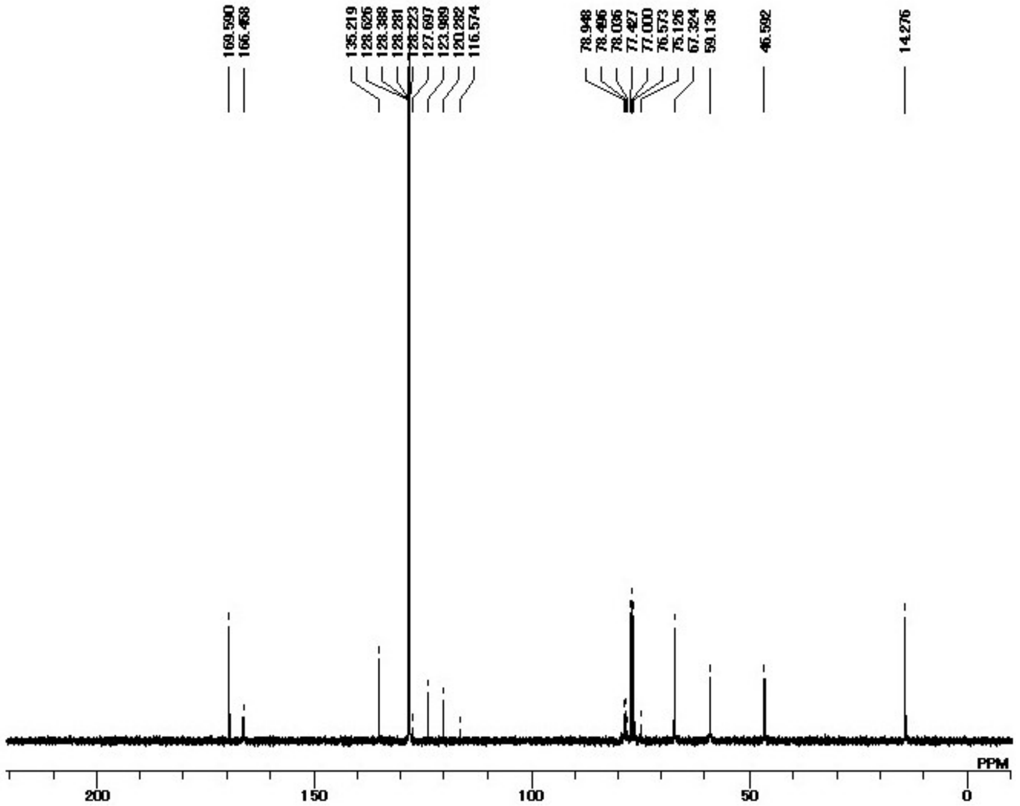

3-Ethyl *anti*,*syn*-tetrahydro-2-oxo-4-(trifluoromethyl)furandicarboxylate (***anti*,*syn*-8a**)

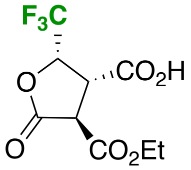

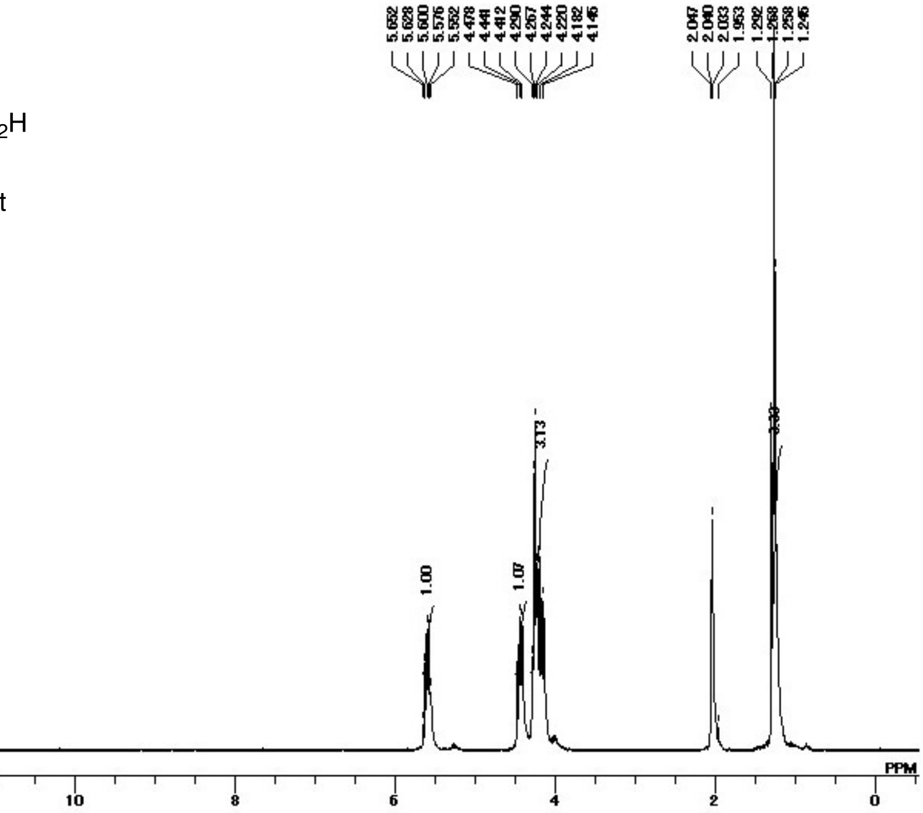

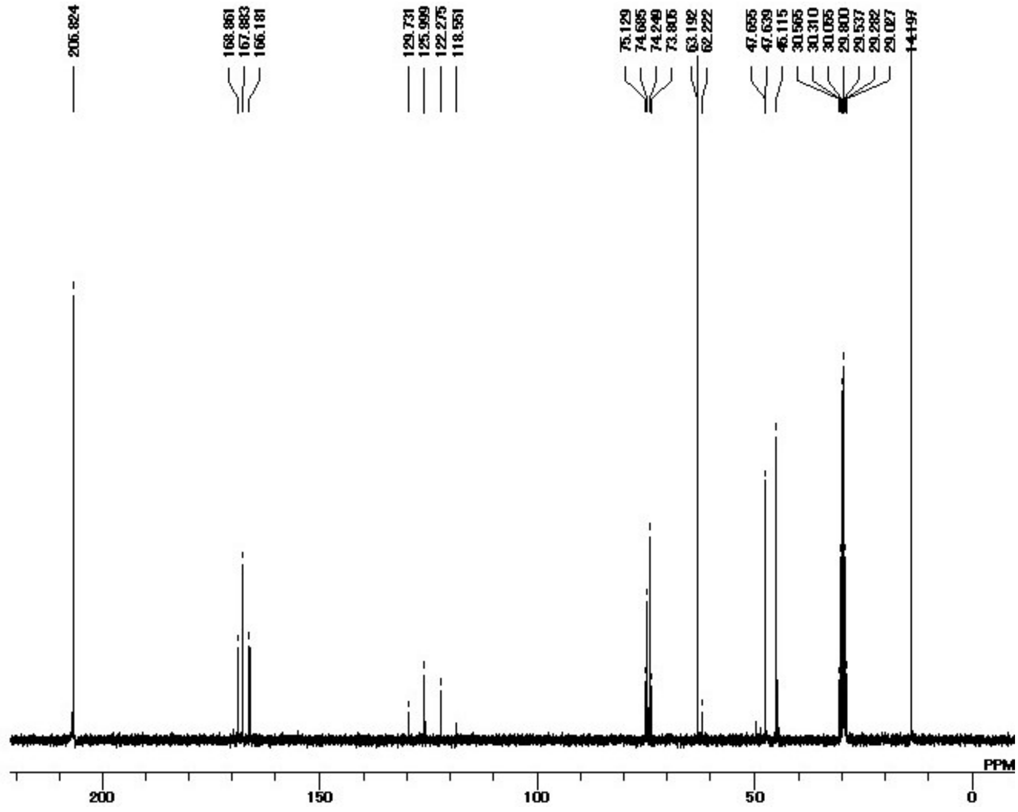

Benzyl 2,3-*anti*-2-{cyano(ethoxycarbonyl)methyl}-4,4,4-trifluoro-3-hydroxybutanoate (***anti*-9e**)

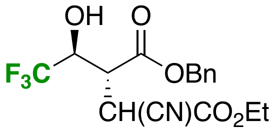

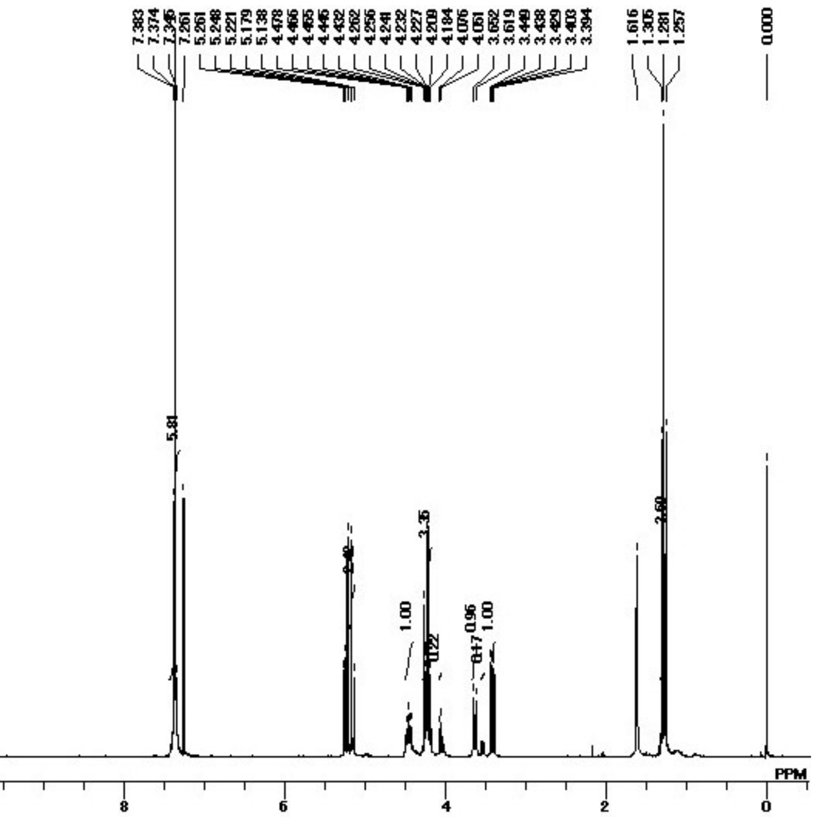

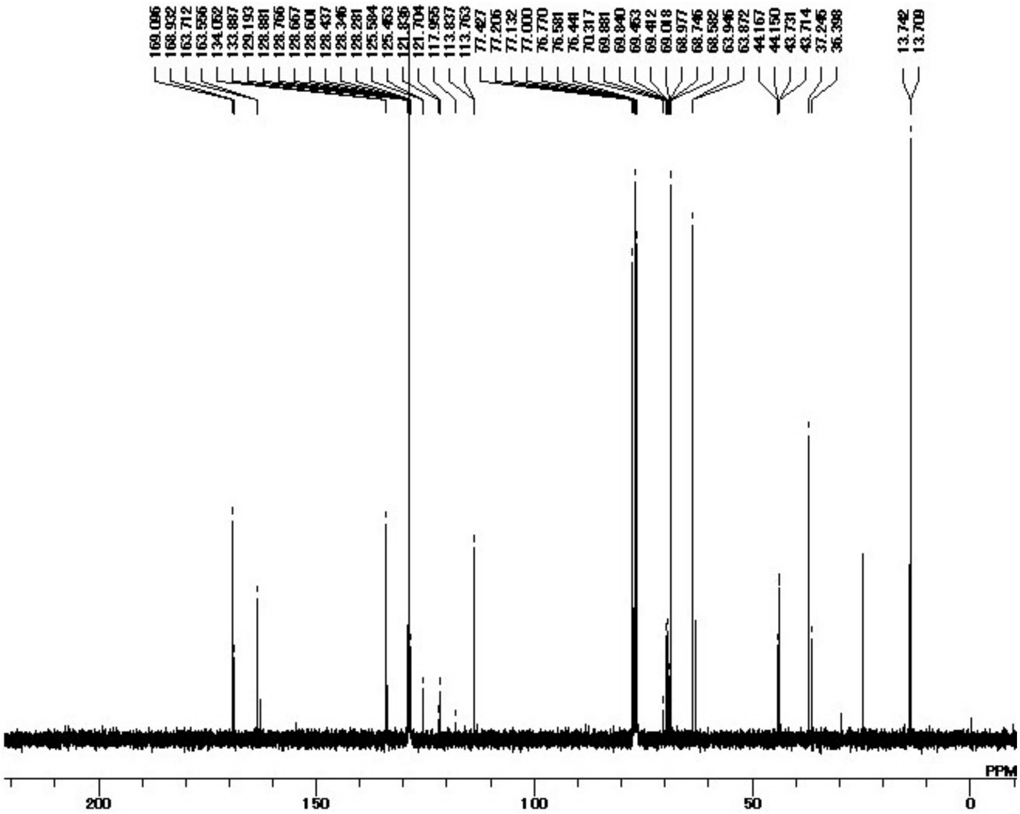

Benzyl 2,3-*anti*-4,4,4-trifluoro-2,3-dihydroxybutanoate (***anti*-10a**).

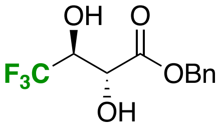

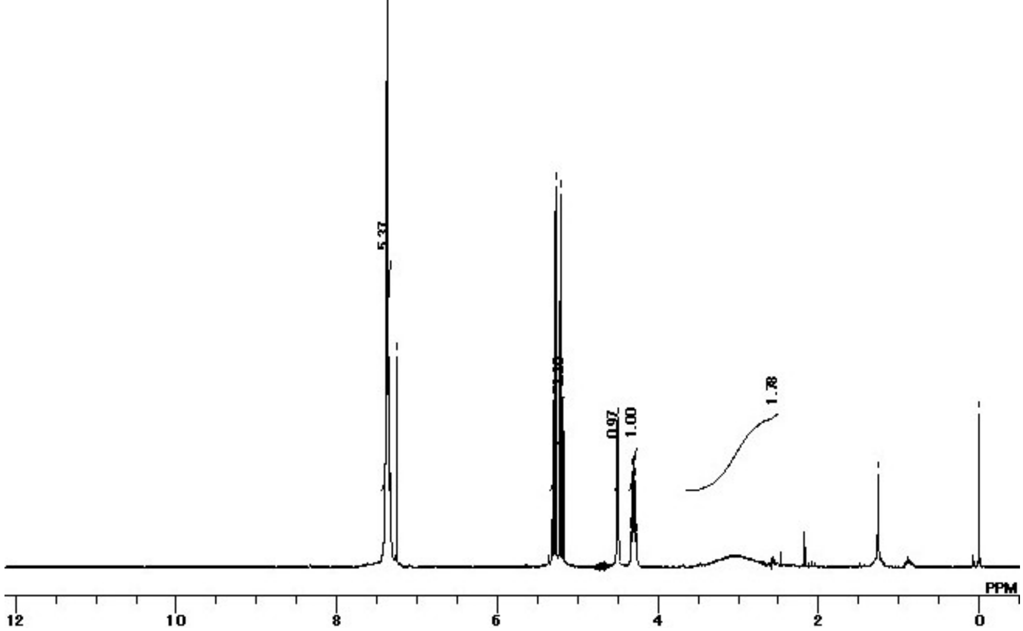

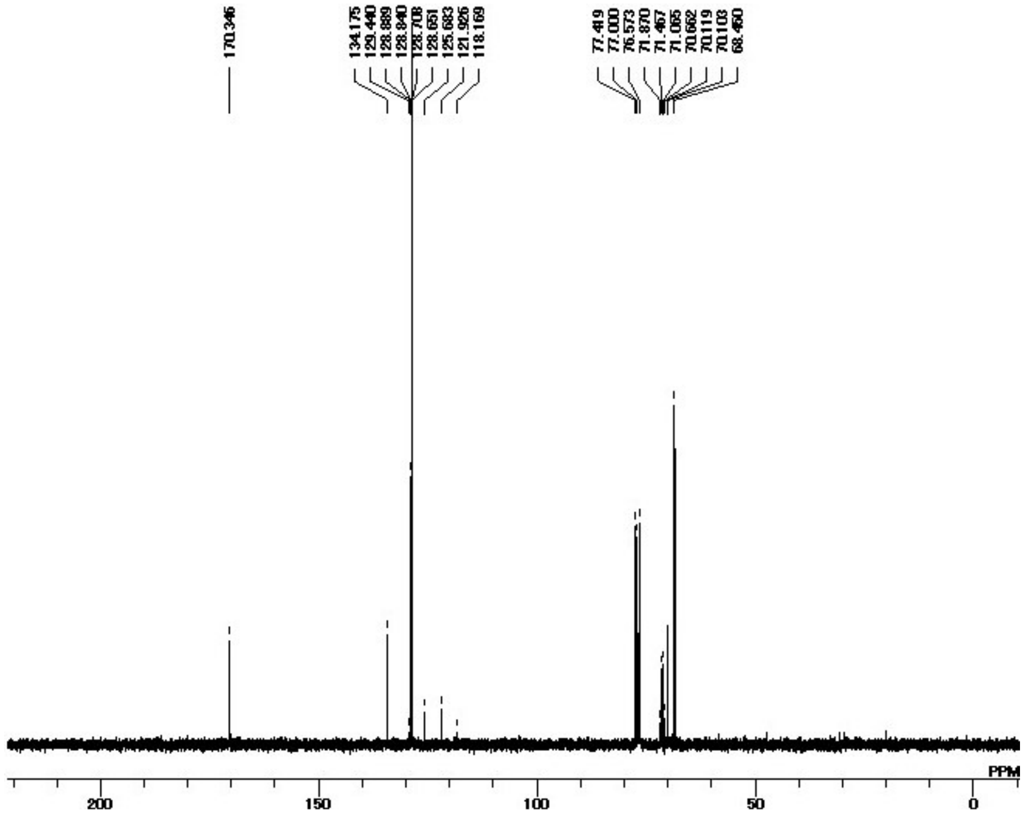

1,1,1-Trifluoro-2-hydroxytetradecan-4-one (**11a**)

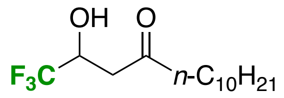

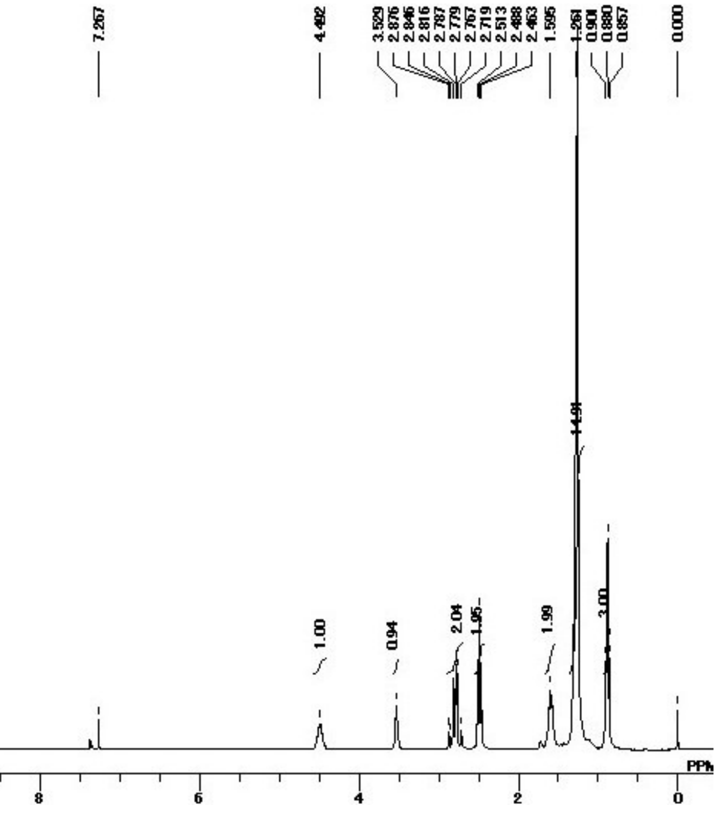

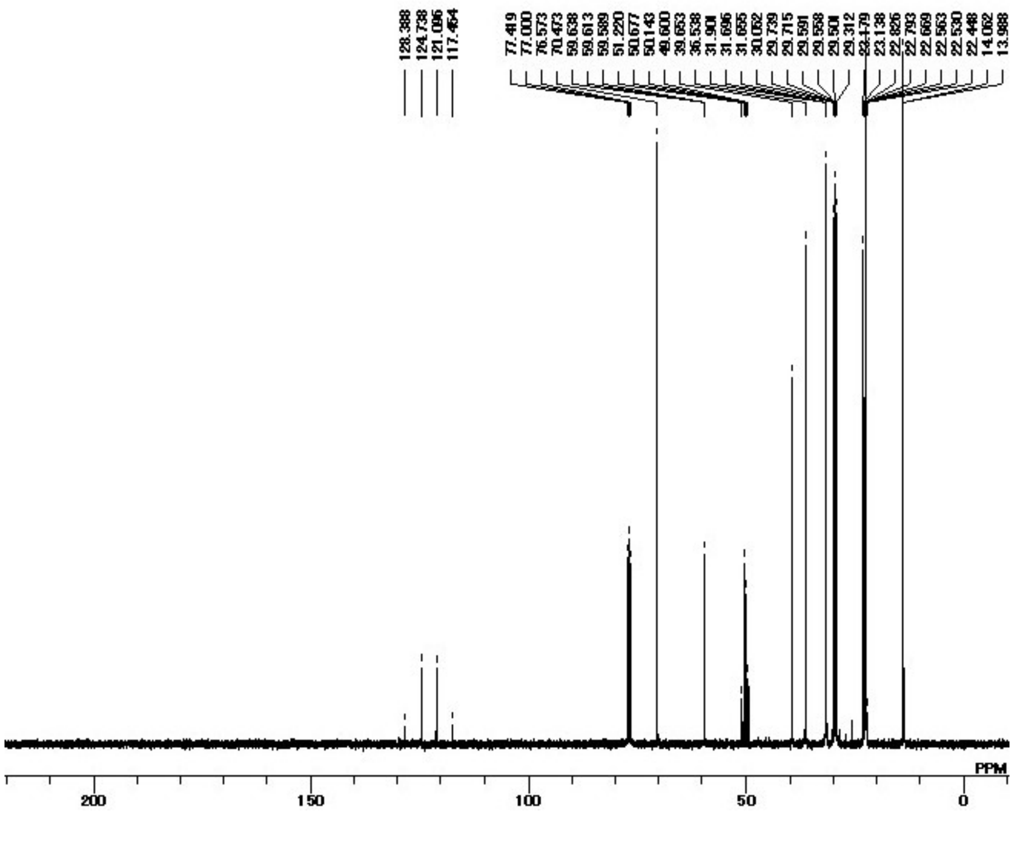

(*E*)-4-Decyl-2,3-epoxy-4,4,4-trifluorotetradecan-4-ol (**12a**)

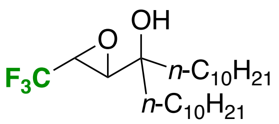

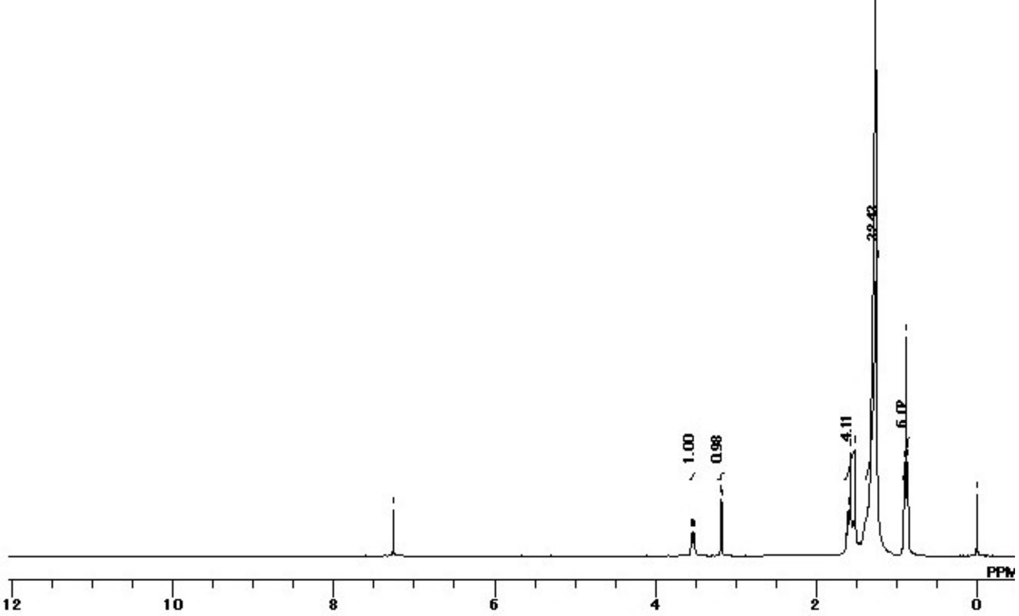

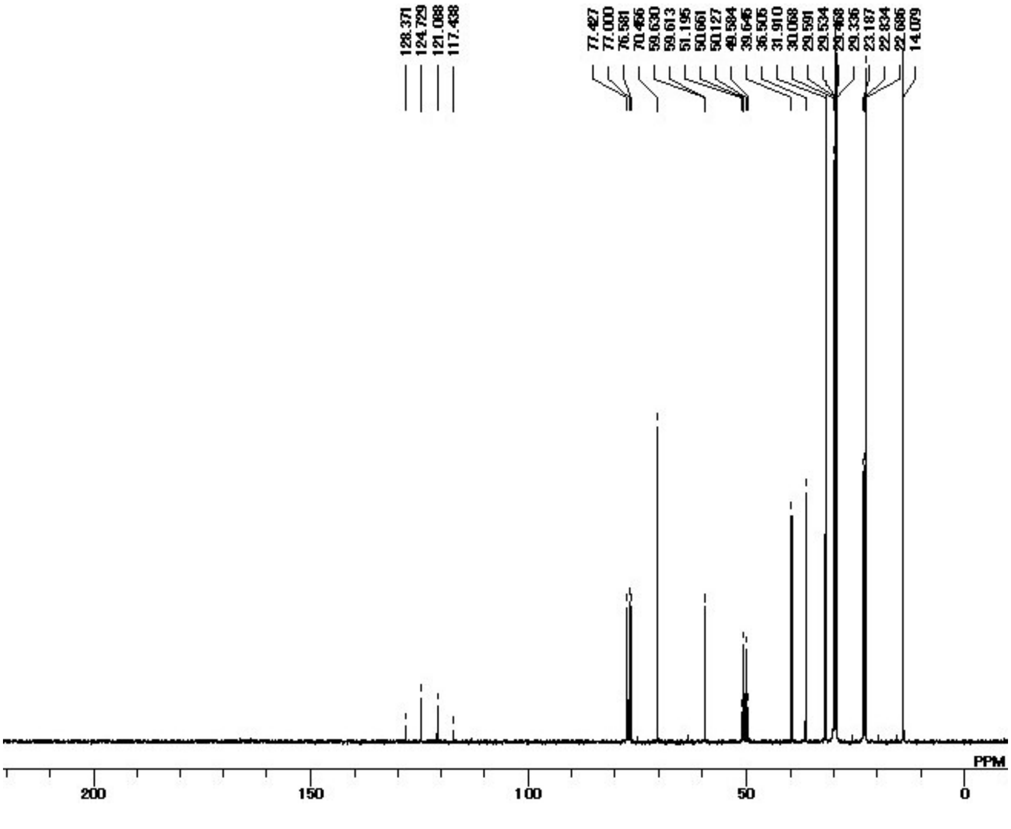

(*E*)-2,3-Epoxy-4,4,4-trifluoro-1,1-bis(4-methoxyphenyl)butan-1-ol (**12e**)

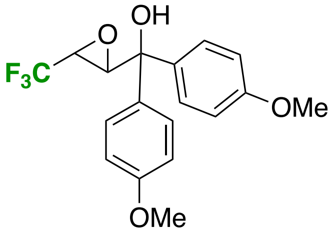

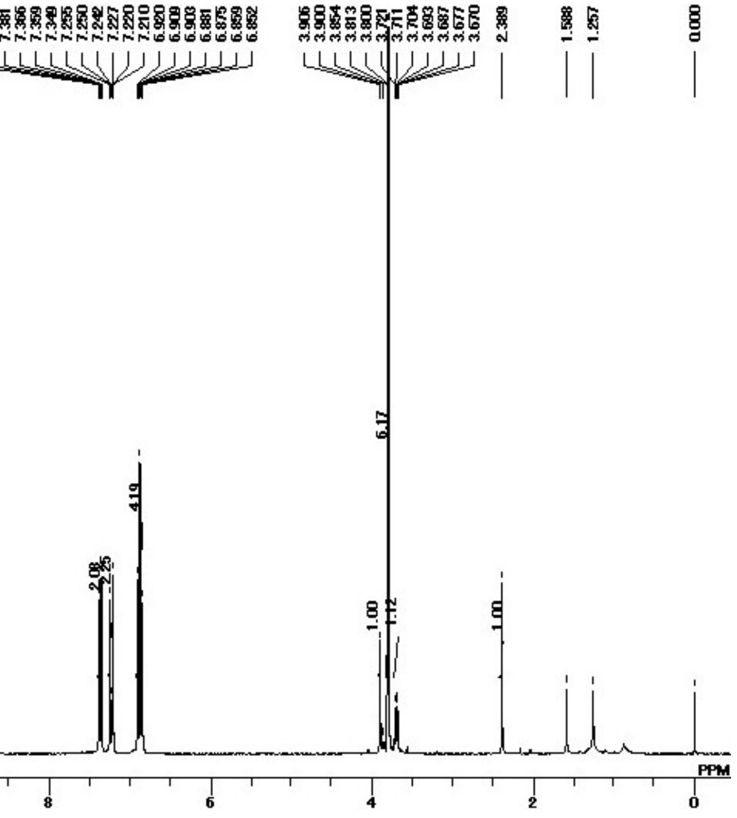

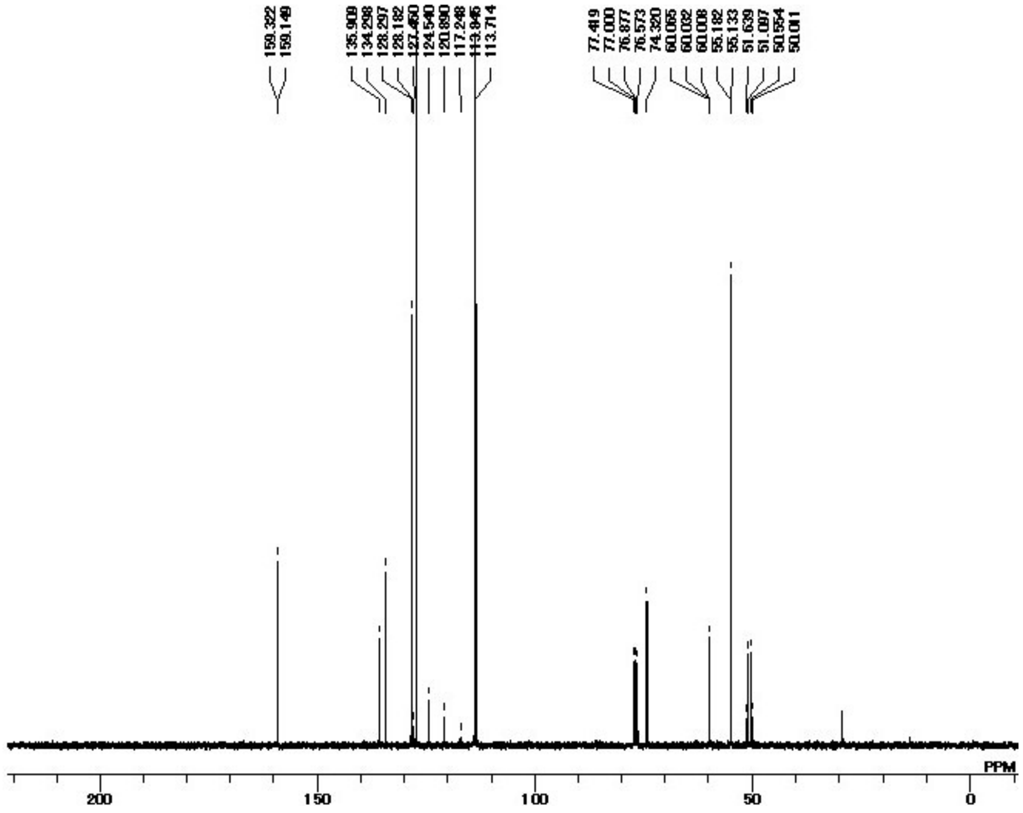

2,3-Epoxy-1,1,1-trifluoro-4-*n*-hexyltetradecan-4-ol (**12f**)

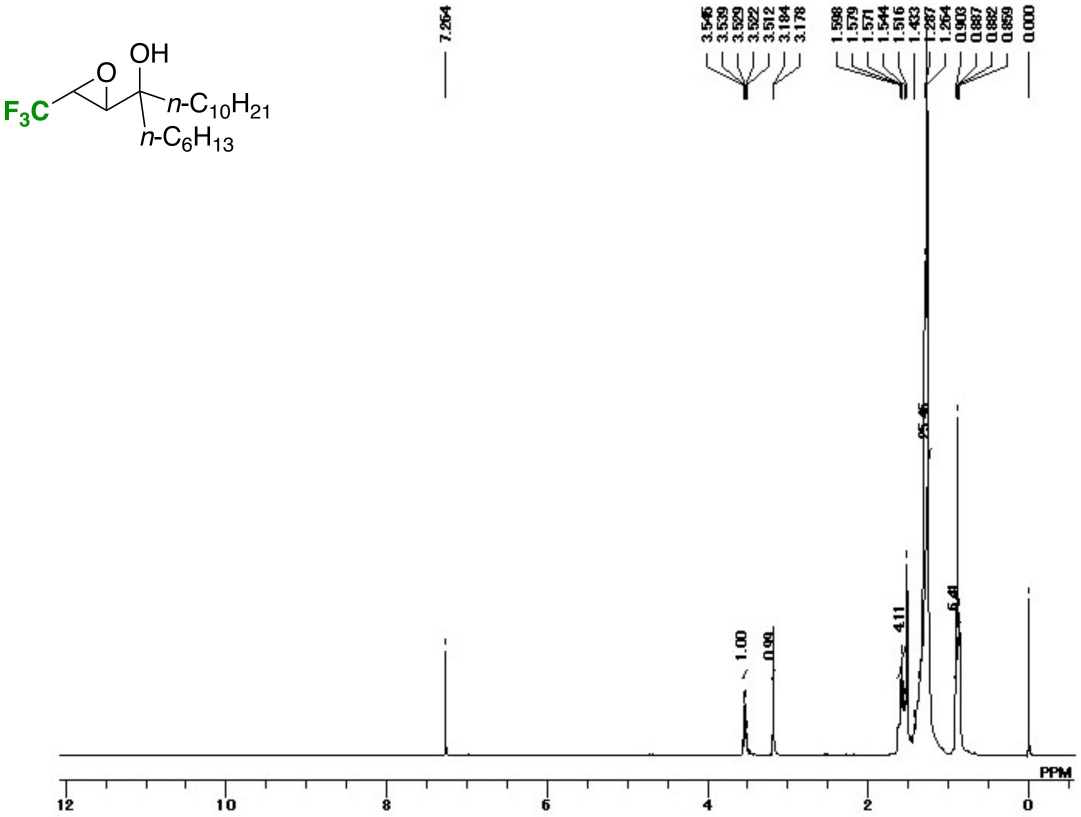

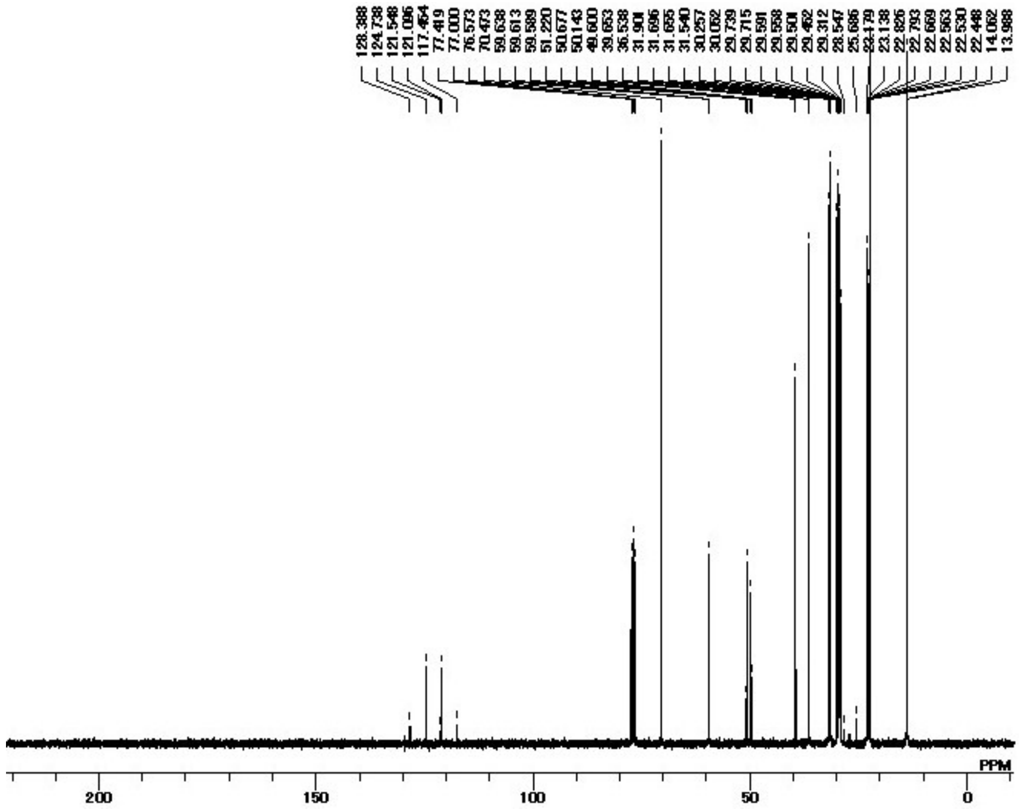

1,1,1-Trifluoro-3-deutero-2-hydroxytetradecan-4-one (**11a-D**)

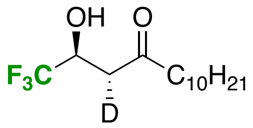

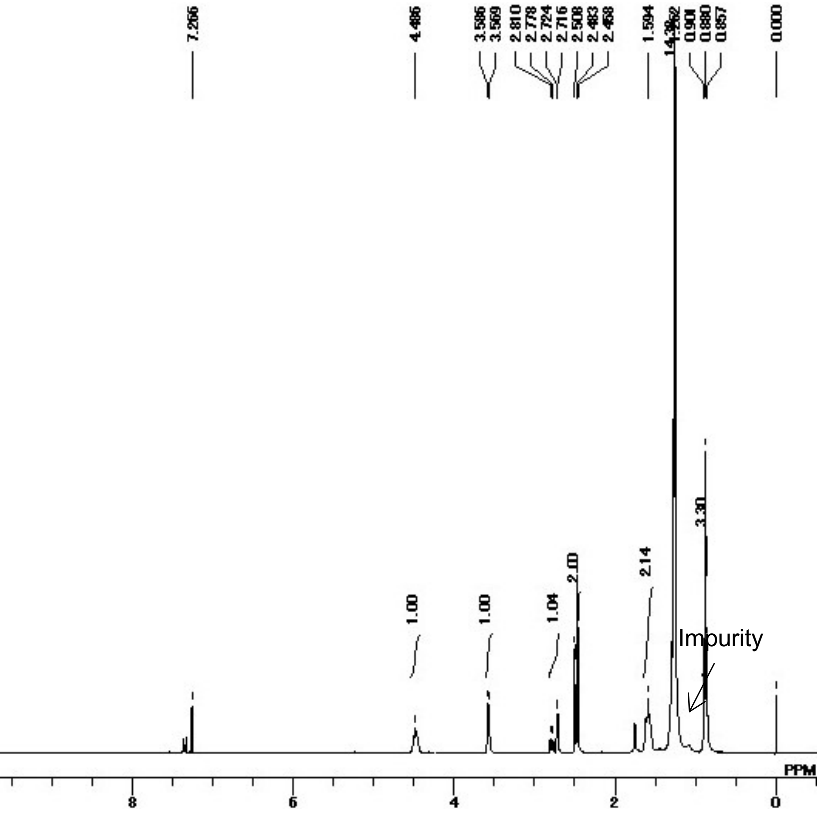

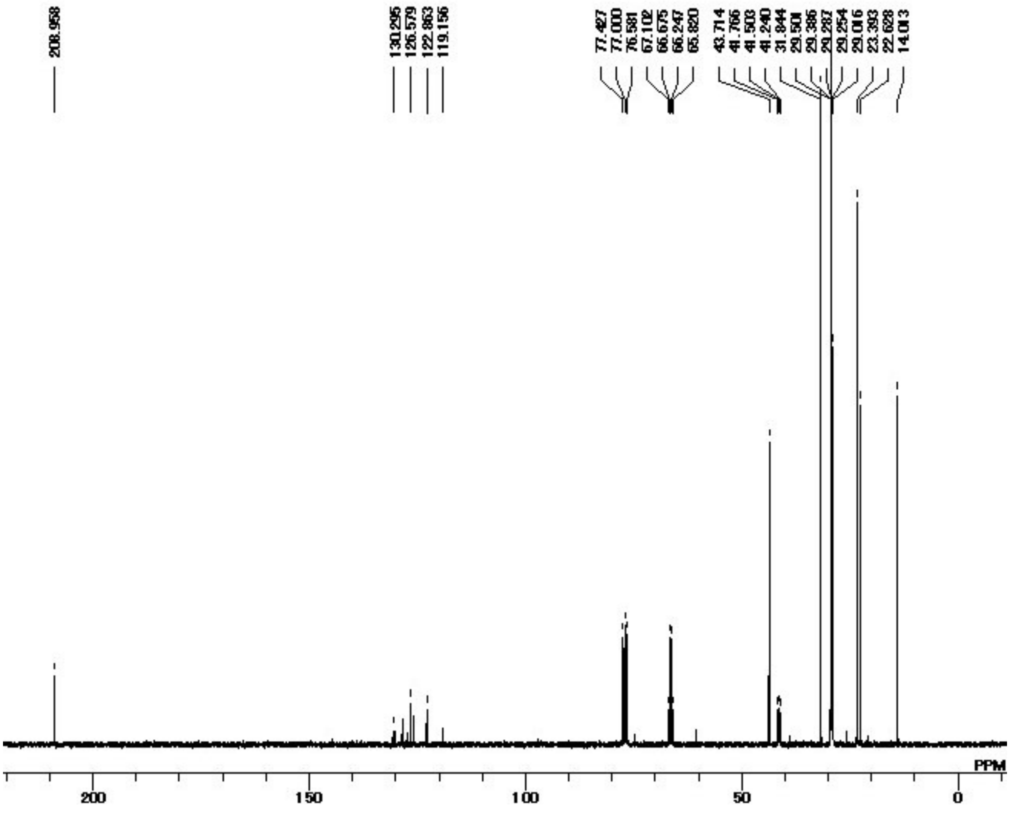

(*E*)-2,3-Epoxy-4,4,4-trifluorotetradecan-4-one (**13a**)

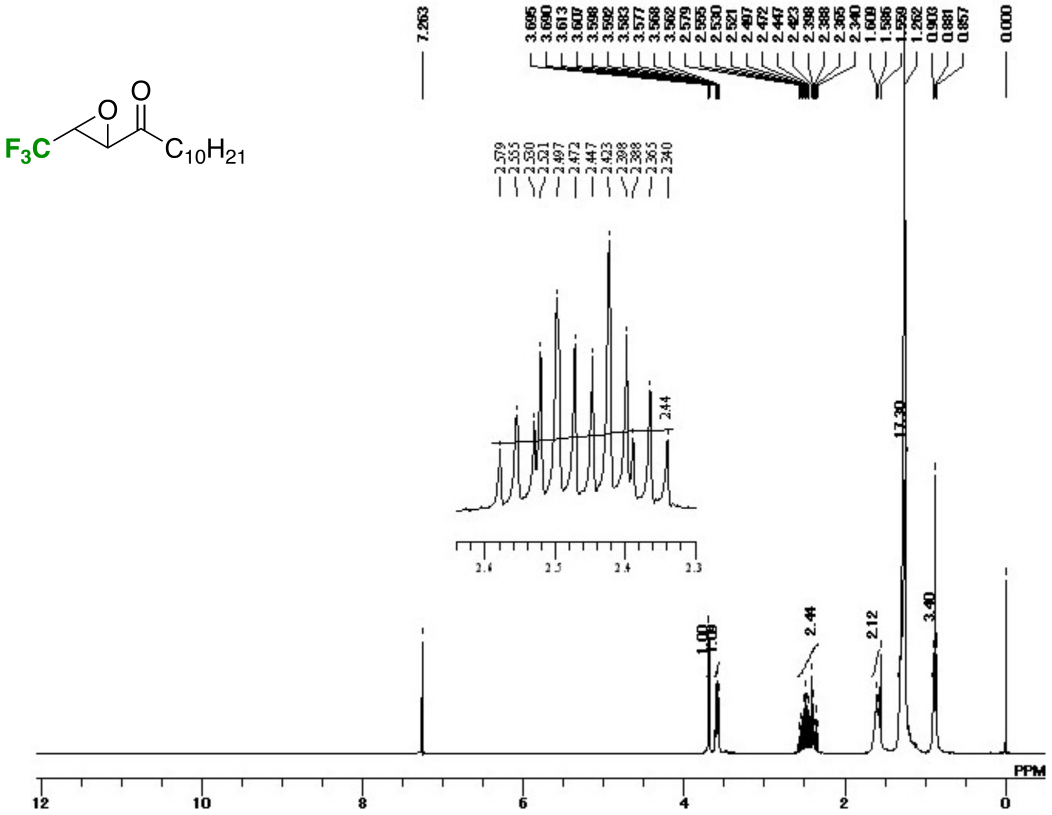

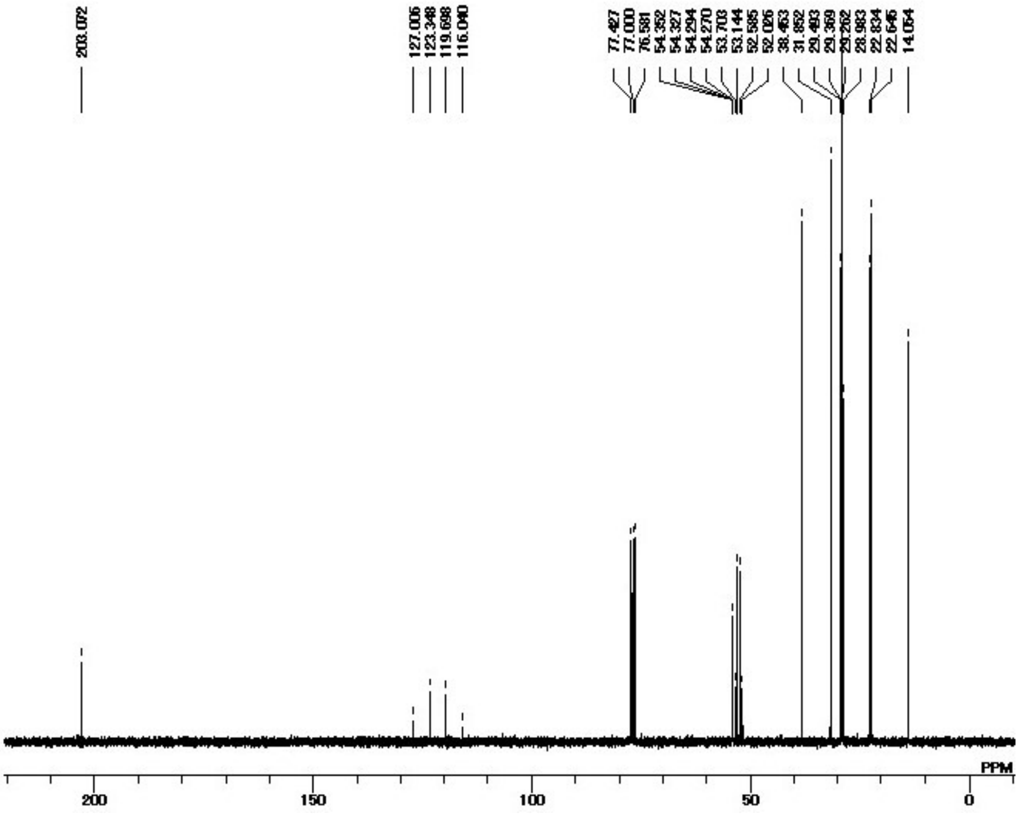

Crystallographic Data

Benzyl (2*R,3*S**)-2,3-*anti*-2-((2'*R**)-1-phenylethylamino)-4,4,4-trifluoro-3-hydroxy-butanoate ((2*R**,3*S**,2'*R**)-3bd, minor isomer)**

Bond precision:	C-C = 0.0019 A	Wavelength = 0.71073	
Cell:	a = 16.9751(6)	b = 11.0427(4)	c = 19.9654(7)
	α = 90	β = 93.329(3)	γ = 90
Temperature:	173 K		

	Calculated	Reported
Volume	3736.2(2)	3736.2(2)
Space group	C 2/c	C 1 2/c 1
Hall group	-C 2yc	-C 2yc
Moiety formula	C19 H20 F3 N O3	C19 H20 F3 N O3
Sum formula	C19 H20 F3 N O3	C19 H20 F3 N O3
Mr	367.36	367.36
Dx,g cm-3	1.306	1.306
Z	8	8
Mu (mm-1)	0.108	0.108
F000	1536.0	1536.0
F000'	1537.01	
h,k,lmax	22,14,25	21,14,25
Nref	4285	4279
Tmin,Tmax	0.961,0.971	0.962,1.000
Tmin'	0.954	

Reported T Limits:	Tmin = 0.962	Tmax = 1.000
AbsCorr	MULTI-SCAN	
Data completeness	0.999	
Theta(max)	27.483	
R(reflections)	0.0390 (3601)	
wR2(reflections)	0.1012 (4279)	
S	1.075	
Npar	240	

Benzyl 2,3-*anti*-4,4,4-trifluoro-3-hydroxy-2-(phenylmethylsulfenyl)butanoate (4ba)

Bond precision:	C-C = 0.0027 A	Wavelength = 0.71073	
Cell:	a = 8.8481(2)	b = 10.3154(3)	c = 11.0154(3)
	α = 65.382(3)	β = 77.591(2)	γ = 74.638(3)
Temperature:	173 K		

	Calculated	Reported
Volume	875.06(5)	875.06(5)
Space group	P -1	P -1
Hall group	-P 1	-P 1
Moiety formula	C18 H17 F3 O3 S	C18 H17 F3 O3 S
Sum formula	C18 H17 F3 O3 S	C18 H17 F3 O3 S
Mr	370.38	370.38
Dx,g cm-3	1.406	1.406
Z	2	2
Mu (mm-1)	0.229	0.229
F000	384.0	384.0
F000'	384.50	
h,k,lmax	11,13,14	11,13,14
Nref	4018	4012
Tmin,Tmax	0.940,0.954	0.929,1.000
Tmin'	0.940	

Reported T Limits	Tmin = 0.929	Tmax = 1.000
AbsCorr	MULTI-SCAN	
Data completeness	0.999	
Theta(max)	27.484	
R(reflections)	0.0381 (3356)	
wR2(reflections)	0.1015 (4012)	
S	1.085	
Npar	227	

3-Ethyl *anti,syn*-tetrahydro-2-oxo-4-(trifluoromethyl)furandicarboxylate (*anti,syn*-8a)

Bond precision:	C-C = 0.0051 A	Wavelength = 0.71073	
Cell:	a = 5.7515(8)	b = 18.893(3)	c = 10.1628(16)
	α = 90	β = 91.906(13)	γ = 90
Temperature:	213 K		

	Calculated	Reported
Volume	1103.7(3)	1103.7(3)
Space group	P 21/c	P 21/c
Hall group	-P 2ybc	-P 2ybc
Moiety formula	C9 H9 F3 O6	C9 H9 F3 O6
Sum formula	C9 H9 F3 O6	C9 H9 F3 O6
Mr	270.16	270.16
Dx,g cm-3	1.626	1.626
Z	4	4
Mu (mm-1)	0.166	0.166
F000	552.0	552.0
F000'	552.53	
h,k,lmax	6,22,12	6,22,12
Nref	2039	2038
Tmin,Tmax	0.963,0.982	0.308,1.000
Tmin'	0.963	

Reported T Limits	Tmin = 0.308	Tmax = 1.000
AbsCorr	MULTI-SCAN	
Data completeness	1.000	
Theta(max)	25.498	
R(reflections)	0.0699 (1474)	
wR2(reflections)	0.2227 (2038)	
S	1.082	
Npar	165	

References

1. Brendan, M.; O'Leary, B. M.; Szabo, T.; Sventrup, N.; Schalley, C. A.; Lutzen, A.; Schafer, M.; Rebek Jr, J. *J. Am. Chem. Soc*. **2001**, *123*, 11519–11533. doi:10.1021/ja011651d
2. Yamazaki, T.; Mano, N.; Hikage, R.; Kaneko, T.; Kawasaki-Takasuka, T.; Yamada, S. *Tetrahedron* **2015**, *71*, 8059–8066. doi:10.1016/j.tet.2015.08.048
3. Tamura, K.; Yamazaki, T.; Kitazume, T.; Kubota, T. *J. Fluorine Chem.* **2005**, *126*, 918–930. doi:10.1016/j.jfluchem.2005.04.001
4. Yajima, T.; Jahan, I.; Tonoi, T.; Shinmen, M.; Nishikawa, A.; Yamaguchi, K.; Sekine, I.; Nagano, H. *Tetrahedron* **2012**, *68*, 6856–6861. doi:10.1016/j.tet.2012.06.028
5. Rueping, M.; Albert, M.; Seebach, D. *Helv. Chim. Acta* **2004**, *87*, 2473–2486. doi:10.1002/hlca.200490222
6. Sakavuyi, K.; Petersen, K. S. *Tetrahedron Lett.* **2013**, *54*, 6129–6132. doi:10.1016/j.tetlet.2013.08.132
7. Betz, P.; Krueger, A. *ChemPhysChem* **2012**, *13*, 2578–2584. doi:10.1002/cphc.201101050
8. Xiong, H.-Y.; Yang, Z.-Y.; Chen, Z.; Zeng, J.-L.; Nie, J.; Ma, J.-A. *Chem. Eur. J.* **2014**, *20*, 8325–8329. doi:10.1002/chem.201403073
9. Funabiki, K.; Matsunaga, K.; Nojiri, M.; Hashimoto, W.; Yamamoto, H.; Shibata, K.; Matsui, M. *J. Org. Chem.* **2003**, *68*, 2853–2860. doi:10.1021/jo026697j
10. von dem Bussche-Hünnefeld, C.; Seebach, D. *Chem. Ber.* **1992**, *125*, 1273–1281. doi:10.1002/cber.19921250538
11. Marrec, O.; Borrini, J.; Billard, T.; Langlois, B. R. *Synlett* **2009**, 1241–1244. doi:10.1055/s-0029-1216748
12. Yamazaki, T.; Ichige, T.; Kitazume, T. *Org. Lett.* **2004**, *6*, 4073–4076. doi:10.1021/ol048229x
